# Reassessment of the risks of climate change for terrestrial ecosystems

**DOI:** 10.1038/s41559-024-02333-8

**Published:** 2024-02-26

**Authors:** Timo Conradi, Urs Eggli, Holger Kreft, Andreas H. Schweiger, Patrick Weigelt, Steven I. Higgins

**Affiliations:** 1 Plant Ecology, University of Bayreuth, Bayreuth, Germany; 2 Sukkulenten-Sammlung Zürich, Grün Stadt Zürich, Zürich, Switzerland; 3 Biodiversity, Macroecology & Biogeography, University of Göttingen, Göttingen, Germany; 4 Centre of Biodiversity and Sustainable Land Use (CBL), University of Göttingen, Göttingen, Germany; 5 Campus-Institute Data Science, Göttingen, Germany; 6 Institute of Landscape and Plant Ecology, Department of Plant Ecology, University of Hohenheim, Stuttgart, Germany

**Keywords:** Climate-change ecology, Biogeography

## Abstract

Forecasting the risks of climate change for species and ecosystems is necessary for developing targeted conservation strategies. Previous risk assessments mapped the exposure of the global land surface to changes in climate. However, this procedure is unlikely to robustly identify priority areas for conservation actions because nonlinear physiological responses and colimitation processes ensure that ecological changes will not map perfectly to the forecast climatic changes. Here, we combine ecophysiological growth models of 135,153 vascular plant species and plant growth-form information to transform ambient and future climatologies into phytoclimates, which describe the ability of climates to support the plant growth forms that characterize terrestrial ecosystems. We forecast that 33% to 68% of the global land surface will experience a significant change in phytoclimate by 2070 under representative concentration pathways RCP 2.6 and RCP 8.5, respectively. Phytoclimates without present-day analogue are forecast to emerge on 0.3–2.2% of the land surface and 0.1–1.3% of currently realized phytoclimates are forecast to disappear. Notably, the geographic pattern of change, disappearance and novelty of phytoclimates differs markedly from the pattern of analogous trends in climates detected by previous studies, thereby defining new priorities for conservation actions and highlighting the limits of using untransformed climate change exposure indices in ecological risk assessments. Our findings suggest that a profound transformation of the biosphere is underway and emphasize the need for a timely adaptation of biodiversity management practices.

## Abstract (web summary)

Combining ecophysiological growth models of >135,000 vascular plant species and information on plant growth form, the authors show that 33–68% of the global land surface will experience a significant change in the next 50 years in how climate supports the plant growth forms that define terrestrial ecosystems.

## Main

Global circulation models (GCM) forecast strong climatic changes throughout the twenty-first century under all but the most optimistic greenhouse gas emission scenarios^1^. The anticipated climatic changes are expected to force both continuous and abrupt changes in the distribution of ecosystems and species^2,3^. One implication is that ecosystem managers may have to shift their focus from targeting predefined baseline states to managing ecosystem change along trajectories forced by climatic change^4–6^. However, the strength of climatic forcing and the direction of the new trajectories are uncertain, making it difficult for ecosystem managers to define and implement targeted actions^7,8^. Not only are climates changing but it is also likely that climate states without present-day analogues (hereafter, novel climates) will emerge and that some of the existing climate states will disappear (hereafter, disappearing climates)^9,10^. It has been suggested that disappearing climates may increase the risk of losing species and some types of ecosystems, whereas novel climates may lead to the formation of novel ecosystems^9,11,12^. Since the functioning of novel ecosystems is by definition unknown, their emergence would further enhance the risk of ecosystem management failure^9,12,13^. It is thus a research priority to identify regions where climate change is likely to force strong ecological change, so that ecosystem managers can implement timely and targeted actions.

To identify regions with elevated ecological risks from climate change, previous works analysed the exposure of the global land surface to potentially dangerous climatic changes^10^. This includes analyses of the exposure of ecosystems to strong climatic changes and globally novel climates, as well as the disappearance of existing climate states^9,14^. In these studies, exposure was calculated as the Euclidean distance between ambient and future climates, standardized by the historical variability in climate variables. Another widely used climate change exposure metric is climate change velocity^15–17^, which quantifies the displacement rate of climate states and a more recent species-focused study identified where and when animal species will be exposed to temperature and precipitation conditions outside their realized niches^3^.

However, risk assessments based on climate change exposure indices oversimplify an organism’s perception of climate exposure. Previous climate change exposure work did not consider that physiological and ecological responses to changing climatic factors are often nonlinear and colimited by several climatic factors^18–21^ and that hierarchies of colimiting climatic factors are expected to reorganize as climate change progresses^22,23^. This means that the risk of a one unit increase in a climatic factor is not comparable across locations with different baseline values and is contingent on concomitant changes in colimiting climatic factors. Neither this baseline effect nor the colimitation effect are accounted for by climate change exposure studies. Moreover, individual species and growth forms exhibit contrasting climatic preferences^24^ and are therefore likely to respond differently to projected climatic changes. The implication is that the ecological response to altered climatic conditions is unlikely to map perfectly to climate change exposure indices^9,25^, which makes the ecological interpretation of climate change exposure indices problematic.

Problems with the ecological interpretation of exposure indices could be addressed by using process-rich ecosystem simulation models^26,27^. Such models are explicitly designed to model the impacts of climatic changes on ecosystems. However, ecosystem simulation models are hampered by process and parameter uncertainty^26–28^, evidenced by large discrepancies between outputs of different models forced with the same climate data^29^. The implication is that current approaches to understand ecological climate change risks and impacts trade-off the certainty with which we can make predictions with the ability to ecologically interpret those predictions. Exposure indices have more tractable prediction uncertainty, yet are difficult to interpret ecologically, whereas the opposite is true for ecosystem models.

**Fig. 1:**
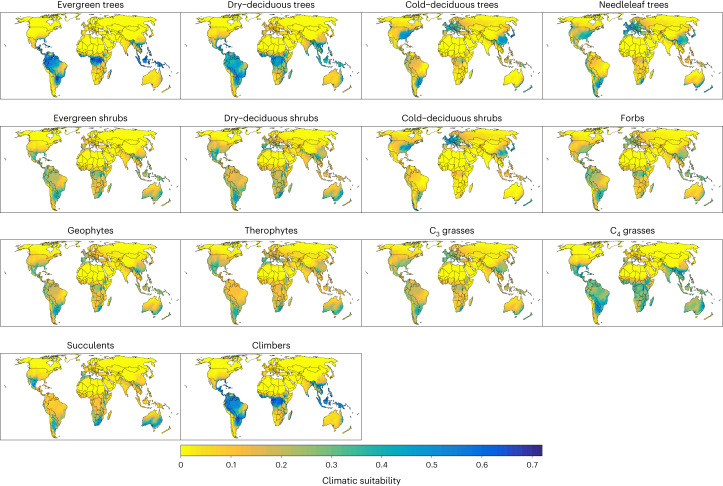
Ambient climatic suitability for major plant growth forms. Suitability is expressed as the proportion of plant species of a growth form for which the climate of the cells is suitable according to an ecophysiological plant growth model. The median number of modelled species per growth form was 5,249, with a minimum of 439 (needleleaf trees) and a maximum of 24,853 (evergreen shrubs) species. The total number of modelled species was 135,153.

To reduce this trade-off, we computed a phytoclimatic transform of ambient (mean of 1979–2013) and future (mean of 2061–2080; henceforth 2070) climatologies. The phytoclimatic transform expresses the climate of a grid cell in terms of its suitability for species of 14 plant growth forms that define terrestrial ecosystems. The transformation was based on an existing protocol^24^ (Supplementary Fig. 1) and involved (1) parameterizing an ecophysiological plant growth model forced with monthly climate data for 135,153 vascular plant species, (2) using the fitted species models to identify climatically suitable grid cells for each species, (3) calculating the proportion of species of each growth form for which a grid cell is climatically suitable and using this proportion as an index of the climatic suitability of a grid cell for a growth form (Fig. 1). We refer to the vector of the 14 growth-form suitabilities of a grid cell as its phytoclimate. That is, rather than describing the climate of a location by climate variables such as mean annual temperature or annual rainfall, we describe the climate by its ability to support species of different types of plants that ecologists use to define terrestrial ecosystems. This provides a means to understand which structural changes in ecosystems the future climatic states will promote. For instance, should the climatic suitability of a grid cell for cold-deciduous trees change from 0.2 to 0.3, this would mean that the climate can now accommodate an extra 10% of species of this growth form in that grid cell, which raises the potential for species of this growth form to become more frequent.

The premise of this analysis is that we can characterize changes in the climatic forcing of ecosystems by analysing shifts in climatic suitability for the growth forms that define ecosystems. Previous work showed that the growth-form suitability values are useful predictors of ecosystem states^30^, suggesting that shifts in growth-form suitability may be useful indicators of the changing climatic forcing. It should be emphasized that the growth-form suitability may be quite different from growth-form abundances because other ecological processes such as biotic interactions, dispersal or disturbances, determine whether physiological suitability can predict abundance. Our analysis therefore provides a physiologically informed assessment of where and how the climatic drivers of ecosystem assembly are changing.

The plant growth model used here describes how the uptake and allocation of carbon and nitrogen of an individual plant is colimited by monthly temperature, soil moisture, solar radiation and atmospheric CO_2_ concentrations^24,31,32^. We used species distribution data from the BIEN database (https://bien.nceas.ucsb.edu/bien/) to find the physiological parameters of the plant growth model that best explain the observed distribution of a species. For each of the 135,153 species, the parameterized model is then run forward using a monthly gridded climatology to simulate the biomass accumulation of the species in the cells of a global grid and the simulated biomass values are used as the linear predictor in a logistic regression model that predicts whether grid cells are climatically suitable for that species or not. That is, we use data on the observed distribution of species to estimate the parameters of a model that articulates a simplified physiological niche of a plant species^32^. The performance of the model in transferability tests^33^ indicates that this physiological niche characterization is predictive of where a plant species could grow.

**Fig. 2:**
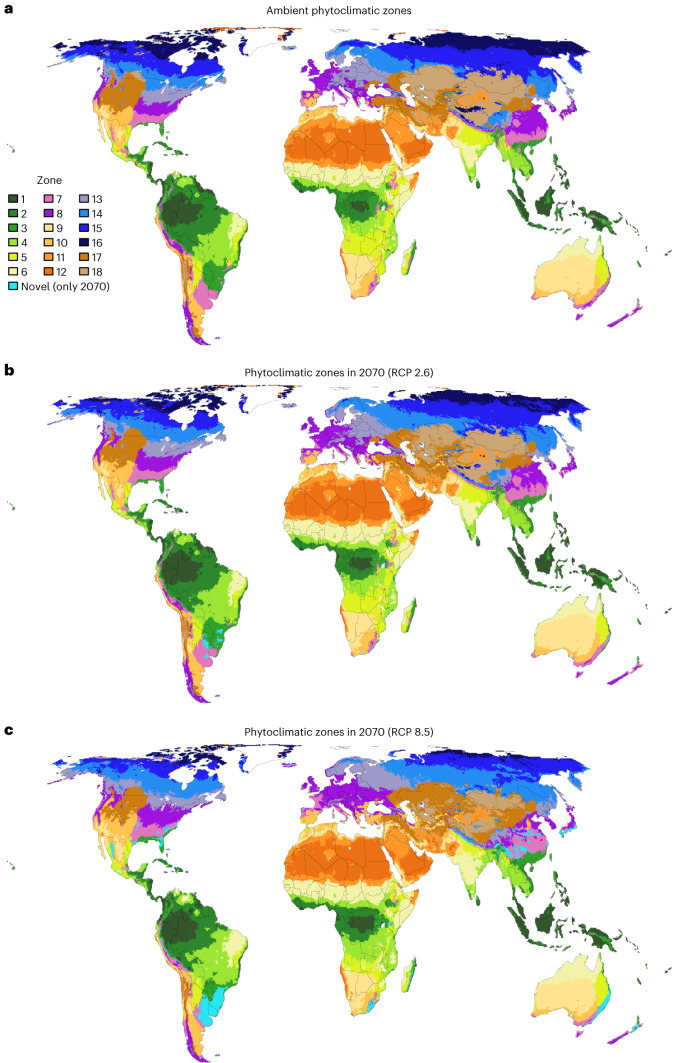
Phytoclimatic zones of the Earth and their shifts by 2070. Phytoclimatic zones have internally similar climatic suitability for different plant growth forms. **a**, Ambient phytoclimatic zones, derived from an unsupervised classification of grid cells by their climatic suitability for 14 plant growth forms (Fig. 1). **b**,**c**, The arrangement of phytoclimatic zones in 2070 under RCP 2.6 (**b**) and RCP 8.5 (**c**). The median climatic suitabilities for each growth form across five future climatologies were used to determine the future phytoclimatic zone of the grid cells for each RCP. Cells shown as ‘novel’ are projected to have growth-form suitability realizations without present-day analogue. Extended Data Fig. 5 shows shifts in phytoclimatic zones for different combinations of RCP and GCM.

The growth-form suitability surfaces in Fig. 1 represent a summary of estimated physiological niches of 135,153 plant species grouped by growth form. We aggregate this phytoclimatic transform of the climate by identifying groups of cells with similar growth-form suitability using unsupervised classification. The geographical projection of these groups reveals phytoclimatic zones of the Earth (Fig. 2a) and provides a plant growth-form centric classification of the climates of the Earth. Supplementary Table 1 and Supplementary Fig. 2 provide overviews of the mean growth-form suitabilities of the ambient phytoclimatic zones. Supplementary Fig. 3 shows that the phytoclimatic zones have distinct climatic characteristics.

The phytoclimatic zones depicted in Fig. 2a represent areas where the climate supports similar plant types, making the concept closely related to biomes. Indeed, previous work suggested that phytoclimatic zones could be used to define biomes^24^. Yet, biomes are mostly defined as regions where specific combinations of plant growth forms have developed in response to the regional climate^34,35^. That is, biomes implicitly consider additional ecological processes that shape the ecosystem structure observed in the field, whereas phytoclimatic zones consider only how the climate influences the physiological performance of plant types. This means that phytoclimatic zones (for example, Fig. 2) are more closely affiliated with bioclimatic zones^36,37^ than with biomes.

**Extended Data Fig. 1:**
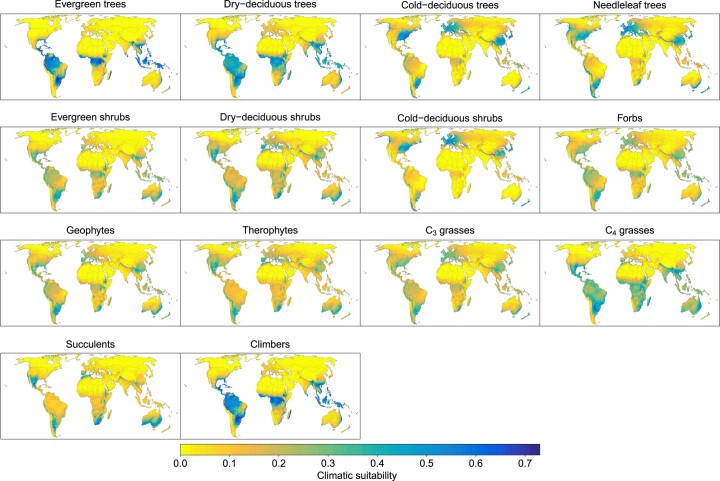
Climatic suitability for major plant growth forms in 2070 under RCP 2.6. Suitability is expressed as the proportion of plant species of a growth form for which the cells’ climate is suitable according to an ecophysiological plant growth model. The values are means across climatologies projected by five Global Circulation Models. Note that the range of the legend is slightly larger than that in Fig. 1 in the main text.

**Extended Data Fig. 2:**
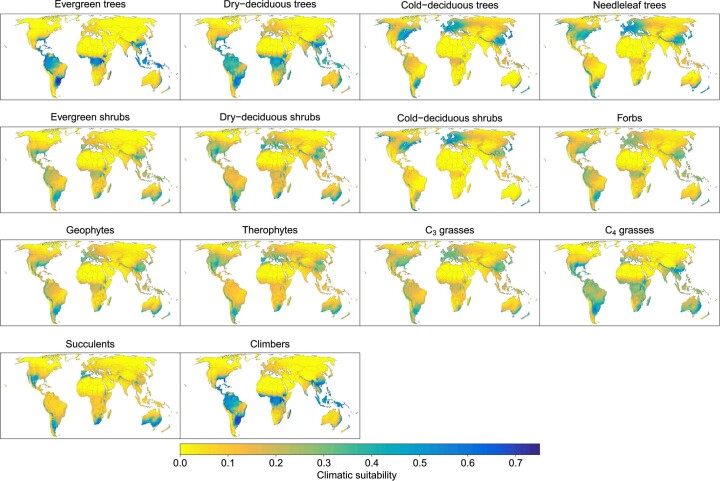
Climatic suitability for major plant growth forms in 2070 under RCP 8.5. Suitability is expressed as the proportion of plant species of a growth form for which the cells’ climate is suitable according to an ecophysiological plant growth model. The values are means across climatologies projected by five Global Circulation Models. Note that the range of the legend is slightly larger than that in Fig. 1 in the main text.

The phytoclimatic transformation was then applied to climatologies projected for 2070 under the representative concentration pathways RCP 2.6 and RCP 8.5, which describe a reduced and a high emission scenario, respectively. Specifically, we forced the fitted species growth models with the future climatologies to project climatic suitability maps for each species and grouped species by growth form to calculate growth-form suitability maps for 2070 (Extended Data Figs. 1 and 2).

Analogous to work on climate change exposure^9^, we used the ambient and future growth-form suitability maps to calculate three multivariate Euclidean distances that summarize components of ecological risk: (1) The local phytoclimatic change each grid cell will be exposed to, estimated as the distance between the ambient and future growth-form suitabilities of each grid cell. This index summarizes the changing climatic constraints on the plant growth forms in each cell. (2) The novelty of the future phytoclimatic state of each grid cell relative to ambient phytoclimatic states, estimated as the minimum distance between the future growth-form suitabilities of a grid cell and that of an ambient grid cell. A novel phytoclimate is thus a climate that constrains plant growth forms differently to any ambient climate state. (3) The disappearance of the ambient phytoclimatic state of each grid cell, estimated as the minimum distance between the ambient growth-form suitabilities of a grid cell and those of a future grid cell. The disappearing phytoclimate index is thereby a measure of how distinct the ambient phytoclimatic state of a grid cell is relative to future phytoclimatic states. A disappearing phytoclimate indicates disappearance of a way in which an ambient climate constrains plant growth forms.

To interpret which values of the three indices indicate high risks, we use the Euclidean distances between the phytoclimatic zones shown in Fig. 2a as a reference. Specifically, we computed the centroids of each zone in Euclidean growth-form suitability space and calculated the pairwise Euclidean distances between these centroids. The 5th percentile of these intercentroid distances was used as a threshold value to identify ecologically significant risk equivalent to a shift between some of the phytoclimatic zones. Lastly, the future phytoclimatic zone of grid cells was projected by assigning grid cells to the zone of the closest ambient grid cell in Euclidean growth-form suitability space; grid cells that were further than the threshold distance from ambient phytoclimatic states were designated as novel.

## Results

**Fig. 3:**
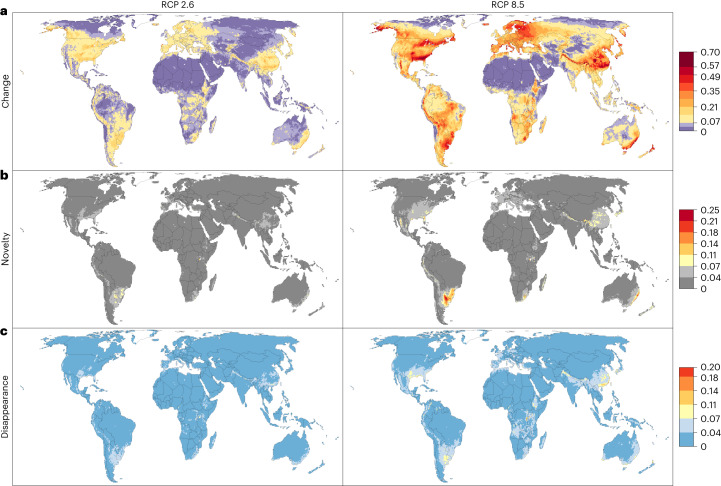
Change, novelty and disappearance of phytoclimates by 2070 under RCP 2.6 and RCP 8.5. The phytoclimate of the grid cells is the suitability of the local climate for 14 plant growth forms that characterize the structure of terrestrial ecosystems. **a**, Local change in phytoclimate, expressed as the Euclidean distance between the ambient and future phytoclimates of a cell. **b**, Novelty of the projected phytoclimate in 2070, expressed as the Euclidean distance of the future phytoclimate of a cell to its closest analogue in the global pool of ambient phytoclimates. **c**, Risk of disappearance of the existing phytoclimate, expressed as the Euclidean distance of the ambient phytoclimate of a cell to its closest analogue in the global pool of future phytoclimates. In **a**–**c**, RCP 2.6 is shown in the left column and RCP 8.5 in the right column; the colour bars are scaled so that yellow to red colours indicate significant values of change, novelty and disappearance, respectively (see main text for the definition of the threshold value and Supplementary Figs. 4–6 for the sensitivity of the results to varying the threshold value). Values are medians across future climatologies generated by five different GCMs for each RCP.

**Extended Data Fig. 3:**
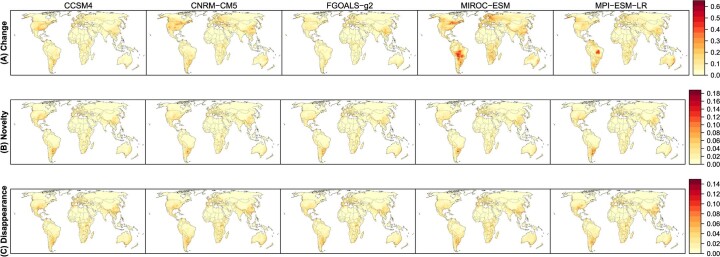
(A) Change, (B) novelty and (C) disappearance of phytoclimates by 2070 under RCP 2.6 for five different Global Circulation Models. The phytoclimate of the cells is the suitability of the local climate for 14 plant growth forms that characterize the structure of terrestrial ecosystems. (**a**) Local change in phytoclimate. (**b**) Novelty of the projected phytoclimate in 2070, expressed as the Euclidean distance of a cell’s future phytoclimate to its closest ambient analogue. (**c**) Risk of disappearance of the existing phytoclimate, expressed as the Euclidean distance of a cell’s ambient phytoclimate to its closest future analogue.

**Extended Data Fig. 4:**
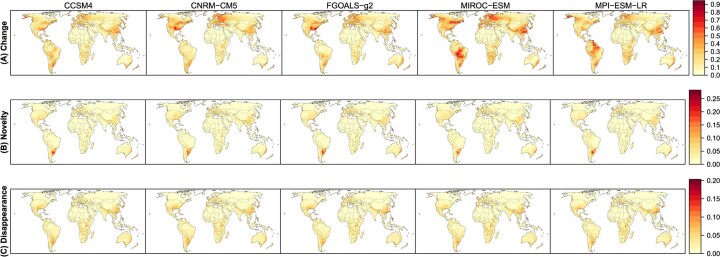
(A) Change, (B) novelty and (C) disappearance of phytoclimates by 2070 under RCP 8.5 for five different Global Circulation Models. The phytoclimate of the cells is the suitability of the local climate for 14 plant growth forms that characterize the structure of terrestrial ecosystems. (**a**) Local change in phytoclimate. (**b**) Novelty of the projected phytoclimate in 2070, expressed as the Euclidean distance of a cell’s future phytoclimate to its closest ambient analogue. (**c**) Risk of disappearance of the existing phytoclimate, expressed as the Euclidean distance of a cell’s ambient phytoclimate to its closest future analogue.

Our analysis predicts substantial change in phytoclimates by 2070 (Fig. 3a). If anthropogenic emissions follow RCP 2.6, 33% of the Earth’s terrestrial surface (excluding the currently ice-covered parts of Greenland and Antarctica) will experience an ecologically significant change in the extent to which the climate can support different plant growth forms. The fraction of land with significant change in phytoclimate increases to 68% if emissions follow RCP 8.5. When calculating the median change value across five projections that used climatologies from different GCMs, the most pronounced phytoclimatic changes are likely to occur in the mountain regions of south China, the Himalayas, northwestern Russia, the Baltic countries, Scandinavia, the southeastern and northeastern United States, Alaska, central Mexico, the tropical Andes, southeastern South America, southeastern Australia and northern New Zealand. Projections based on each GCM are shown in Extended Data Figs. 3a and 4a.

**Extended Data Fig. 5:**
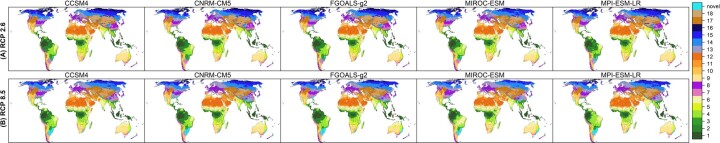
Projected distribution of phytoclimatic zones in 2070 under different climate change scenarios. Shown is the projected distribution of phytoclimatic zones in 2070 under (A) RCP 2.6 and (B) RCP 8.5 when using climate data from five different Global Circulation Models.

**Extended Data Fig. 6:**
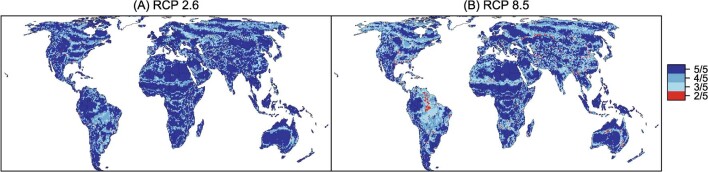
Agreement on phytoclimatic zone in 2070 between five projections that used 2070 climate data from different Global Circulation Models. A value of 5/5 means that all five projections made the same 2070 phytoclimatic zone prediction. A value of 2/5 means that only two of five projections made the same 2070 phytoclimatic zone prediction.

The changes in local phytoclimates translate into widespread shifts of phytoclimatic zones (Fig. 2b,c). Temperate, boreal and polar regions are most strongly affected by these shifts (Supplementary Fig. 7). Under RCP 8.5, large parts of zones 13 and 14, which currently support cool-temperate and hemiboreal ecosystems, will shift polewards, as will zone 15, which currently supports boreal ecosystems. Zone 16, which currently supports tundra ecosystems, will be reduced by 72% as it cannot shift further north. This prediction is consistent with observations of more frequent recruitment of shrubs and trees in tundra^38–41^ and increasing tree density at the forest–tundra ecotone^42^. Zone 18, which supports ecosystems of very continental cool-temperate regions will lose 54% of its current extent, mostly to zone 17, which favours less cold-limited continental cool-temperate ecosystems (Supplementary Fig. 3) and is generally more suitable for most growth forms (Supplementary Fig. 2). Tropical phytoclimatic zones are overall less likely to change in position and extent. However, some regions will be exposed to changes in phytoclimatic zones that may force strong structural changes in ecosystems. For instance, we project that the southeastern and eastern parts of the Amazon will shift to a phytoclimatic zone which supports savanna and some types of drier forest ecosystems (phytoclimatic zone 4 in Fig. 2). This projection is consistent with analyses that have used hydrological thresholds to project future changes in South American forests and savannas^43^. Phytoclimatic zone 4 is predicted to advance into the Amazon irrespective of GCM, albeit to very different degrees (Extended Data Fig. 5), suggesting that the future phytoclimatic status of the Amazon region is highly uncertain (Extended Data Fig. 6).

**Extended Data Fig. 7:**
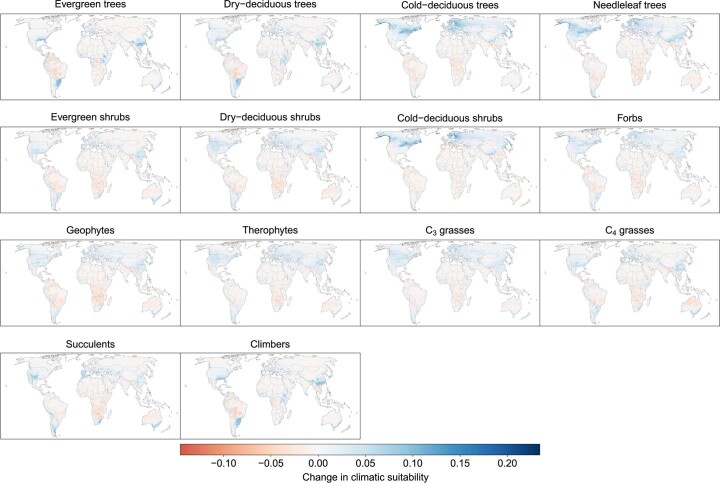
Change in climatic suitability of grid cells for plant growth forms between ambient and 2070 climatologies under RCP 2.6. Change is expressed as change in proportion of species of a growth form that can grow in a cell according to our simulations. Values are medians across five projections that used 2070 climate data from different Global Circulation Models.

**Extended Data Fig. 8:**
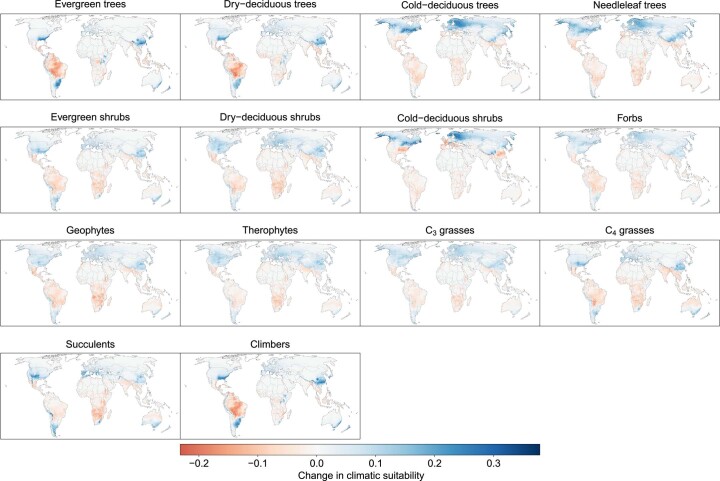
Change in climatic suitability of grid cells for plant growth forms between ambient and 2070 climatologies under RCP 8.5. Change is expressed as change in proportion of species of a growth form that can grow in a cell according to our simulations. Values are medians across five projections that used 2070 climate data from different Global Circulation Models.

Despite the strong changes in local phytoclimates and substantial shifts in phytoclimatic zones, most of the future phytoclimates will have a present-day analogue. Specifically, only 0.3% (RCP 2.6) to 2.2% (RCP 8.5) of land cells will exhibit novel phytoclimates (Fig. 2b,c). The highest novelty indices are forecast in southeastern South America and Australia (Fig. 3b). The novel phytoclimates are forecast to primarily emerge in what at present are mesic subtropical climates typical of the eastern sides of continents but they are also likely in tropical and subtropical mountain ranges such as the Andes, the Himalayas and the Sierra Madre Occidental. Most grid cells in the South American and Australian novelty centres belong to the ambient phytoclimatic zone 7, which is characterized by relatively high suitability for all growth forms (Supplementary Fig. 2). These cells are predicted to exhibit a strongly increased climatic suitability for evergreen trees, dry-deciduous trees and climbers by 2070 but reduced suitability for needleleaf trees, cold-deciduous shrubs and cold-deciduous trees (Extended Data Figs. 7 and 8). The emerging phytoclimatic novelty in these cells may be induced by slightly increasing rainfall combined with similar temperature seasonality, albeit with hotter summers and milder winters. It is likely that this combination reduces the cold-limitation of the numerous species of woody growth forms with preference for mesic tropical and subtropical climates in our analysis, whilst the elevated temperatures disfavour many of the needleleaf and cold-deciduous woody species with preference for temperate conditions (Fig. 1).

Previous analyses of climatic variables found that the highest climatic novelty will emerge in the warmest of the ambient climates in the tropics^9,10^. Our analyses of phytoclimates suggest that these novel tropical climates are not functionally novel. For instance, although parts of the Sahara will be exposed to novel climates^9,10^, we predict that these novel climates will remain highly unsuitable for all plant growth forms, which means that the novel climates are not novel from a plant functional perspective. In the wet tropics where decreases in rainfall and higher temperatures are projected, the novel climates may promote more drought-adapted species, yielding growth-form suitability combinations (phytoclimates) already observed elsewhere. For example, the Amazon and parts of the Cerrado are novel climate regions^9,10^, yet we predict that phytoclimates currently realized in the Cerrado are advancing in the Amazon and are themselves partially replaced by phytoclimates currently realized in the Caatinga (Fig. 2c), meaning that no novel phytoclimates emerge in this region.

Only 0.1% (RPC 2.6) to 1.3% (RPC 8.5) of ambient phytoclimates are projected to disappear by 2070 (Fig. 3d). As with novelty, the disappearance of phytoclimates is forecast to occur primarily in the mesic subtropical climates on the eastern sides of the continents and there was good agreement between the five projections per RCP on where novel and disappearing phytoclimates are located (Extended Data Figs. 3b,c and 4b,c). Disappearing phytoclimates were seldom in the same grid cells that were predicted to support novel phytoclimates. For instance, using the threshold applied in Fig. 3, only 25% of the grid cells with a disappearing phytoclimate were projected to be operating under a novel phytoclimate in 2070, whereas 75% of the cells with disappearing phytoclimates will be operating under a phytoclimate that is found elsewhere today.

The spatial resolution of this analysis can hide details of the geography of changing phytoclimates. While we fitted the species growth models using climatic forcing data with 1 km spatial resolution, our global analysis of phytoclimates used a grid with 25 km spatial resolution. This aggregation removes the (phyto)climatic heterogeneity within the 25 km cells, preventing the detection of finer phytoclimatic patterns in topographic heterogeneous areas such as mountain ranges. For example, the alpine tundra of the European Alps is not visible on our map of phytoclimatic zones (Fig. 2a). A higher resolution would allow the presence and dynamics of such micro-phytoclimates to be projected.

Moreover, while we explored uncertainty originating from RCP and GCM (Extended Data Figs. 3–6), we did not explore uncertainty originating from the model used to estimate climatic suitability for the individual plant species. Our growth-form suitability projections are therefore partly contingent on the assumptions of the growth model used to estimate species-level suitability. While in principle any suitability or species distribution model could be used in the workflow, in practice, many existing alternative models may be inappropriate for this task. First, correlative suitability models have limited transferability^33,44^, producing implausible growth-form suitability surfaces^24^, whereas the process-based TTR-SDM used here was successful in model transferability tests^33^ and produces plausible suitability surfaces^24,30^. Second, alternative models do not consider how elevated atmospheric CO_2_ concentrations may influence photosynthesis and thereby species ranges. Third, sensitivity analyses (Supplementary Fig. 8) show that robust estimates of growth-form suitability surfaces require of the order of hundreds of species distribution models, meaning that only process-based models which can be calibrated for many species are suitable for this task. On another level, uncertainty in the species distribution model needs to be evaluated in the context of other sources of uncertainty, such as uncertainty in the occurrence data, the assignment of species to growth-form classes and the climatic forcing data used to calibrate the models and to project them into the future.

## Discussion

Global greenhouse gas emissions are currently tracking the RCP 8.5 scenario^45^. Under this scenario, our analysis predicts that almost the entire vegetated land surface will be subject to substantial changes in how climate supports the different types of plants that define terrestrial ecosystems (Fig. 3a). This is likely to impact on ecosystem structure, functioning and dynamics. For example, successional trajectories may no longer follow the usual sequences^21,46,47^ and some undesired growth forms may increase in abundance^48^, complicating ecosystem restoration and management. The prospect of changes in ecosystem structure and functioning supports growing calls for ecosystem managers to realign their goals and practices^4–6^. For instance, conservation management in protected areas often seeks to retard change by removing invading species but, if climatic forcing threatens the baseline states, actions that enhance the ability of ecosystems to track climatic forcing may be more effective^5,6^. Tracking climatic forcing would require that managers plan actions that ensure that locally declining species can reach suitable locations elsewhere, for instance species translocations^49^ or enhancing the permeability of agricultural landscapes^50^. Management actions that aim to retard change need to be carefully planned since such actions, if not sustained, will be a waste of resources^51^. Similarly, in ecological restoration it is common to use predefined baseline states as target points^52^ but these targets will become increasingly unattainable as climate deviates from domains that allow the targeted baseline states^47^. Our analysis can be used as a guide to identify locations where future climates will not support current ecosystem states and where managers could consider switching from preservation to managing the trajectories of change^5,6^. Our projections of shifts in phytoclimatic zones (Fig. 2) may serve to reduce uncertainty about these trajectories because they show which type of phytoclimate is expected in a locale in the future and where experience with managing ecosystems under such a phytoclimate may already exist.

Our analysis predicts how the climatic forcing of ecosystems is changing but other factors, not considered in our analysis, will influence the extent and rate at which ecosystems follow the trajectories of climatic forcing. In particular, disturbances or dieback of late-successional vegetation can accelerate change in ecosystems^21^. Other factors, such as dispersal limitation^53^, priority effects of resident communities^54^, persistence of declining remnant populations^55^ or microclimatic buffering by tree canopies^56^ can cause ecosystems to lag behind the climatic forcing, leading to a disequilibrium between climate forcing and ecosystem state. An ecosystem in strong disequilibrium with climate is, however, at high risk of sudden and potentially undesired transitions when the barriers to change have been overcome^32^, underlining that it may be prudent to proactively manage the change in ecosystems to reduce risks associated with mismatches between climate forcing and ecosystem states. This could be achieved by various means including assisted migration, increasing landscape connectivity^49,50,57^ and rewilding^58,59^. All of these require authentic shifts in management paradigms, which may be warranted given that most of the land surface will, according to our analysis, be subject to fundamental changes in climatic forcing.

Novel phytoclimates also have implications for ecosystem management. Novelty in our analysis emerges when the climate supports plant growth forms in unprecedented ways. The emergence of novel phytoclimates confronts biodiversity managers with deeper uncertainty on how ecosystems respond to management actions^13^ and how the structure and function of the ecosystems may change. That is, where novel phytoclimates emerge, managers cannot rely on experience gained by managers elsewhere. The palynological record indicates that no-analogue climates in the last late-glacial period supported vegetation formations without a present-day analogue^60^, suggesting that the novel phytoclimates of the future may, as they have done in the past, give rise to novel ecosystems.

The interpretation of disappearing phytoclimates is that some of the ways in which climate currently supports plant growth forms will not be realized in the future. As these phytoclimates disappear, there is potential for the ecosystem types that form under these phytoclimates to disappear. It follows that regions in which we project disappearing phytoclimates are high-risk areas for biodiversity loss because it is unlikely that these ecosystem states can assemble or be restored elsewhere. We found a coherent pattern in the distribution of disappearing and novel phytoclimates, suggesting priority areas for conservation monitoring and action.

The hotspots of phytoclimatic change, novel phytoclimate emergence and phytoclimate disappearance predicted in this study (Fig. 3) differ clearly from regions of analogous climatic changes identified in previous global studies^9,10^. These studies used climate system variables of broad ecological relevance that were standardized against their interannual variability in the time period covered by the ambient climatology. This standardization emphasizes changes in variables that are large relative to their historical variability because such changes are assumed to have stronger ecological effects and tend to upweight changes in temperature over changes in precipitation since the interannual variability of temperature is often smaller^9^. Our approach, by contrast, uses an ecophysiological growth model to transform monthly climate variables into estimates of climatic suitability for individual species and, by grouping species into growth forms, the climatic suitability for the plant growth forms defining terrestrial ecosystems. That is, the ecological effect of temporal changes in climate variables is prescribed by the fitted plant growth models and summarized in the future growth-form suitability surfaces. This phytoclimatic transformation explains why tropical regions, which are predicted to produce novel climates due to warmer temperatures^9,10^, are not predicted to produce novel phytoclimates as warming proceeds. Specifically, as warming proceeds, the suitability for all growth forms is forecast to decline in the equatorial regions (Extended Data Figs. 7 and 8) and the phytoclimate in these regions will become similar to phytoclimatic zones supporting more drought-adapted and seasonal ecosystems which already exist elsewhere (Fig. 2b,c).

Global greenhouse gas emissions are currently tracking RCP 8.5 predictions^45^ under which severe climatic changes are forecast^1^. Our analysis suggests that these climatic changes are likely to force a profound transformation of the biosphere, ensuring that substantial adaptation measures will be necessary for biodiversity conservation, agriculture and forestry. Our findings, however, also show that changes to the biosphere would be considerably milder under RCP 2.6, which supports the view that cutting greenhouse gas emissions would fundamentally reduce climate change risks for biodiversity, ecosystem functioning and agricultural production.

## Methods

### Environmental data

The plant growth model (described below) is forced with data on monthly minimum, mean and maximum temperature, monthly soil moisture, monthly solar radiation and atmospheric CO_2_ concentrations. Nitrogen uptake is simulated as a function of temperature, soil moisture and soil nitrogen content^32^. In this application of the model we assumed the same soil nitrogen content in all grid cells, which means that plant nitrogen uptake in the model is influenced by temperature and soil moisture only. This is appropriate because this analysis aims to estimate the climatic suitability of geolocations. Ambient monthly temperature data (averages for the period 1979–2013) were downloaded from the CHELSA database^61^ v.1.2. Solar radiation data were downloaded from the Global Aridity and PET Database v.1 (https://csidotinfo.wordpress.com/data/global-aridity-and-pet-database/). We developed a soil moisture model that is similar to that of the Global Aridity and PET Database and predicts monthly soil moisture on the basis of monthly values of precipitation, solar radiation, minimum, mean and maximum temperature and soil field capacity and wilting point, using a Hargreaves-type model of monthly potential evapotranspiration. Ambient monthly precipitation data (averages for the years 1979–2013) were taken from CHELSA v.1.2 and soil field capacity and wilting point data from the Global Gridded Surfaces of Selected Soil Characteristics (IGBP-DIS) dataset^62^. The modelled soil moisture values reflect soil moisture available for evapotranspiration and are not influenced by the vegetation of a grid cell. For model fitting, we assumed an ambient atmospheric CO_2_ concentration of 338 ppm (ref. ^63^). All environmental data were resampled to 1 km resolution if necessary and projected to the equal area World Eckert IV projection, which was used in all analyses and maps.

We downloaded ten downscaled Coupled Model Intercomparison Project Phase 5 (CMIP5) temperature and precipitation climatologies for 2070 (averages for the period 2061–2080) projected by five CMIP5 GCMs under two emission scenarios (RCP 2.6 and 8.5) from CHELSA v.1.2. The five GCMs were: CCSM4 (ref. ^64^), CNRM-CM5 (ref. ^65^), FGOALS-g2 (ref. ^66^), MIROC-ESM (ref. ^67^) and MPI-ESM-LR (ref. ^68^). These models were chosen to represent a wide range of uncertainty in climate change projections originating from different GCMs^69^. Future monthly soil moisture was predicted with our soil moisture model for each of the ten climatologies for 2070. Solar radiation in 2070 was assumed to be the same as today. We focus on RCP 2.6 and RCP 8.5 because the former represents an optimistic emissions reduction scenario whereas the latter represents a pessimistic scenario that assumes continued growth in emissions^70^ and is the scenario the world is currently tracking most closely^45^. We assumed atmospheric CO_2_ concentrations in 2070 of 438 ppm and 677 ppm for RCP 2.6 and RCP 8.5, respectively^63^.

We classified the grid cells of a global map into 20 environmental zones based on the ambient monthly environmental forcing data used by the plant growth model (Supplementary Fig. 9). We used the clara algorithm in the cluster package in R to classify the cells and optimized the separation of cells into the 20 clara clusters by means of a discriminant analysis of principal components (DAPC)^71^ computed from the environmental data. The resulting environmental zones were used later for generating stratified samples of species presence and pseudo-absence records that were used to fit the plant growth model.

### Species distribution and growth-form data

We downloaded distribution data of all vascular plant species in the BIEN database v.4.1 (http://bien.nceas.ucsb.edu/bien/), using the BIEN R package^72^. We used the non-public version of BIEN, which contains sensitive occurrence data of endangered species not included in the public BIEN version and was made available to us upon request.

Most occurrence records in BIEN come from herbarium collections (see Acknowledgments section for collections used in this analysis), ecological plots and surveys^73,74^ as well as from plant trait observations. BIEN also includes data from NeoTropTree (http://www.neotroptree.info/), RAINBIO (http://rainbio.cesab.org/), TEAM (https://www.wildlifeinsights.org/team-network) and The Royal Botanical Garden of Sydney, Australia (https://www.rbgsyd.nsw.gov.au/) and plot data from CVS, CTFS, FIA, NVS, SALVIAS, TEAM, VEGBANK and MADIDI (https://bien.nceas.ucsb.edu/bien/data-contributors/all/). A full list of references for BIEN occurrence records used in this study can be found in Supplementary Table 2.

The R package CoordinateCleaner v.2.0–11 (ref. ^75^) was used to remove records with either zero longitude or latitude and records within a buffer of 10 km around country and province centroids, 5 km around country capitals and 200 m around biodiversity institutions (herbariums, museums or universities), respectively. In addition, we computed country centroids from the Database of Global Administrative Areas v.3.4 and removed records within a 50 km buffer to these centroids. We only retained one occurrence record per 1 km grid cell and species.

The species were grouped into 14 growth forms: evergreen broadleaf trees and shrubs, cold-deciduous and drought-deciduous broadleaf trees and shrubs, needleleaf trees, C_3_ and C_4_ grasses, forbs (excluding geophytes and therophytes), geophytes, therophytes, terrestrial succulents and climbers. All Pinales, except Gnetales and Podocarpaceae, were classified as needleleaf trees, that is all Araucariaceae, Cephalotaxaceae, Cupressaceae, Pinaceae, Sciadopityaceae and Taxaceae. All species of the Poales families Poaceae, Cyperaceae, Juncaceae, Anarthriaceae, Centrolepidaceae, Ecdeiocoleaceae, Joinvilleaceae, Restionaceae, Thurniaceae and Typhaceae were classified as grasses. The database of ref. ^76^ was then used to identify grass species with C_4_ photosynthetic pathway. We classified all species listed in the *Illustrated Handbook of Succulent Plants* as succulents^77^, with updated lists for monocotyledons^78^ and Cactaceae^79^. Woody succulents were treated as succulents and not as trees or shrubs.

For all remaining species, we extracted information on growth form and leaf phenology from BIEN and GIFT v.2.1 (ref. ^80^). We first searched for species-level information in BIEN and filled the gaps with species-level information in GIFT. If growth form and phenology data were still missing for a species, we searched for genus-level information in BIEN and filled the gaps with genus-level information in GIFT. If data were still missing after this step, we searched for family-level information in BIEN and filled the gaps with family-level information in GIFT. If several entries with contrasting information were available (for example, a species had entries as a shrub and a tree), we used the most frequent entry. We assigned species classified in BIEN and GIFT as herb or fern to the forb category. Species classified as graminoids by BIEN and GIFT were also classified as forbs by us, unless they belonged to one of the Poales mentioned above. The forbs were then split into geophytes, therophytes and other forbs using life-form information in GIFT. We assigned species classified in BIEN as climber, liana or vine to the climber category, as well as species classified in GIFT as obligatory climbers, liana or vine. Palms were treated as trees. We excluded tree and shrub species with leaf phenology entries ’variable’ (GIFT) or ‘variable or conflicting information’ (BIEN), aquatic species and all epiphytes (including succulent epiphytes) because they do not use soil moisture and thus cannot be modelled with our approach (GIFT includes a comprehensive global checklist of vascular epiphytes^81^ that was used to identify epiphytes). Cold-deciduousness and drought-deciduousness of trees and shrubs were determined by plotting the BIEN distribution records on a world map of Köppen–Geiger climates^82^. Species with >50% of records in cold climates (Köppen–Geiger zones Cf, D and E) were defined as cold-deciduous and the remaining species were assumed to be drought-deciduous. References for BIEN growth-form data can be found in Supplementary Table 3.

Name matching between data sources was accomplished with the Taxonomic Name Resolution Service^83^, which uses the Missouri Botanical Garden’s Tropicos database (https://tropicos.org), The Plant List (http://www.theplantlist.org, now inactive) and the USDA Plants Database (http://plants.usda.gov).

### Growth and species distribution modelling

We used the TTR-SDM^32^, an ecophysiological species distribution model for plants, to identify climatically suitable grid cells for each plant species. The TTR-SDM is based on Thornley’s transport resistance (TTR) model^31^, which describes in a series of dynamic equations how the biomass accumulation of an individual plant is influenced by the uptake of carbon and nitrogen, their allocation between sinks and sources and growth processes. The TTR-SDM^32^ includes a series of functions that describe how the resource uptake (carbon and nitrogen) and growth processes in the TTR model are influenced by monthly minimum, mean and maximum temperature, soil moisture, solar radiation, soil nitrogen and atmospheric CO_2_ concentration. Supplementary Fig. 10 provides a graphical representation of how these environmental forcing variables influence the resource uptake and growth rates in the TTR-SDM. The model’s equations prescribe the general shape of these relationships (trapezoidal or saturating); however, the values of the forcing variables at which the physiological rates change are species-specific and are estimated for each plant species separately; these values are the model’s parameters (*n* = 18; Supplementary Fig. 10) and we estimate them from species distribution data as described further below.

The model’s equations define that each of the forcing variables can colimit a plant’s resource uptake and growth analogous to Liebig’s law of the minimum and that allocation of the assimilated resources between resource sources and sinks is driven by transport resistance processes^32^. The model is run on a monthly time step using the ambient and the 2070 monthly climatology, respectively, which allows it to explicitly simulate how monthly fluctuations in the forcing variables colimit a plant’s resource uptake, allocation and growth. That is, the model simulates that the monthly biomass accumulation of a plant is colimited by the temperature, soil moisture, solar radiation and soil nutrients that a plant is exposed to each month of a simulation^33^.

The TTR-SDM version used here uses a Farquhar-type photosynthesis model^84^ to describe how potential carbon assimilation rates are colimited by light, temperature and atmospheric CO_2_ concentration and how this colimitation differs for C_3_ and C_4_ plants (details in ref. ^24^). We assume that each species uses either the C_3_ or the C_4_ photosynthetic pathway and use universal parameterizations of the C_3_ and C_4_ Farquhar models. Therefore, each grid cell has a universal maximum rate of monthly C_3_ or C_4_ carbon uptake that is determined by light, temperature and atmospheric CO_2_ and this universal maximum rate can be further reduced by species-specific shoot-nitrogen and soil-water dependencies of carbon uptake (Supplementary Fig. 10).

The prescribed way in which environmental factors influence physiological processes (Supplementary Fig. 10), the simulation of monthly colimitation dynamics and the explicit consideration of CO_2_ effects via a Farquhar-type carbon assimilation model are key conceptual differences to correlative species distribution models. These properties allow the model to extrapolate in physiologically plausible ways to novel data domains, thereby accommodating both novel data ranges and novel combinations of monthly values of climate variables. For example, a model comparison^33,85^ between the TTR-SDM and the widely used correlative SDM Maxent^86^ showed that, although the TTR-SDM has slightly lower ability to describe the species distribution data in the climate-data domain used to fit the models, it had a substantially better ability to describe independent species distribution data outside the climate-data domain used to fit the models. This model comparison provides confidence that the TTR-SDM can identify suitable climatic conditions.

To parameterize the model, one could measure the 18 model parameters shown in Supplementary Fig. 10 in the laboratory but this is not feasible when the goal is to parameterize the model for many species. The alternative is to infer the parameters from species distribution data and gridded climatologies. Conceptually, we achieve this as follows. First, we use the monthly climatic forcing data to simulate the biomass growth of a species at its presence and absence locations. Once the simulated biomass reaches equilibrium with the climate system forcing data, we use the natural log of this simulated biomass as the linear predictor in a logistic regression model that predicts the observed presences and absences of the species. The simulated biomass values are skewed and in such cases the complementary log-log link function is recommended. We therefore use the complementary log-log link function in this study. In practice, using logit or the complementary log-log often does not make a large difference^87^, even if there are theoretical reasons to prefer the complementary log-log. The inference process then uses an optimization algorithm to iteratively improve the likelihood of this regression model by optimizing the 18 parameters of the growth model (Supplementary Fig. 10), which are constrained by the prescribed trapezoidal and step functions defined by the model’s equations^32^. This optimization was performed using the Differential Evolution genetic algorithm^88^, a stochastic optimization method implemented in R in the DEoptim package^89,90^. We allowed the algorithm to iterate 1,000 times, which we found to be sufficient for generating stable parameterizations of the TTR-SDM.

We attempted to fit the TTR-SDM for species with at least seven presence data points. All presence points were used if less than 400 points were available. If more presence points were available, we took a random sample of presence points that conserved the proportions of the 20 environmental zones defined above (Supplementary Fig. 9) in the full set of presence points. For species with more presence records than environmental zones, we then sampled the same number of pseudo-absence points as presence points (the actual number varied slightly due to integer rounding). The probability of selecting a pseudo-absence point in an environmental zone was inversely proportional to the proportions of the zones in the presence point sample, which ensured that zones strongly represented in the presence point sample were less likely to be included in the pseudo-absence points sample. For species with less presence records than environmental zones, we used 20 pseudo-absence points to better constrain the parameter estimation. We found in a pilot study using the benchmarking dataset from ref. ^33^ that our sampling strategy for pseudo-absence points produced parameterizations of the TTR-SDM that had the highest ability to predict independent species distribution data. Other tested sampling strategies used the same stratified strategy as above but with two, five and ten times the number of pseudo-absence points as presence points, stratified sampling of pseudo-absence points without downweighting, random sampling of pseudo-absence points and the target-group approach^91^, each with two, five and ten times the number of pseudo-absence points as presence points. These alternative strategies were found to generate parameterizations of the TTR-SDM with marginally lower predictive accuracy.

Once the final parameterization is found, we use it to simulate the potential biomass of a species in the cells of a global grid using their monthly climatologies. The complementary log-log of the natural log of biomass is then used to calculate a suitability score (0–1). Last, to convert this suitability score into a binary prediction (0,1), we chose a threshold suitability score that maximizes the sum of true positives and true negatives in the presence and (pseudo-)absence data used to parameterize the model. The result is a map showing where the climate is suitable for that species.

The predictive accuracy of the model is then evaluated using the true skill statistic (TSS) calculated from a confusion matrix^92^ that was computed using the abovementioned threshold. Models with low predictive accuracy (TSS ≤ 0.7) were removed. This resulted in fitted models for 135,153 species, consisting of 24,362 evergreen trees, 3,173 dry-deciduous trees, 1,270 cold-deciduous trees, 439 needleleaf trees, 24,853 evergreen shrubs, 2,074 dry-deciduous shrubs, 1,943 cold-deciduous shrubs, 21,903 therophytes, 8,297 geophytes, 23,538 forbs, 6,888 C_3_ grasses, 2,814 C_4_ grasses, 3,609 succulents and 9,990 climbers.

The parameters of the species models were estimated using the above-mentioned environmental forcing data interpolated to a 1 km grid. For subsequent data analyses these species models were projected onto a 25 km grid.

DEoptim is a robust and efficient global optimization algorithm capable of finding optima on irregularly shaped likelihood surfaces^88^. The stochastic nature of the algorithm means that running DEoptim several times for the same species can produce different parameter estimates. This parameter uncertainty produces uncertainty about the potential ranges of individual species, which we use to calculate the proportion of species of each growth form that could grow in the grid cells (the growth-form suitability values shown Fig. 1). The large number of species used in this analysis, however, ensures that the described species-level uncertainty is a negligible source of uncertainty in the growth-form suitability values. Supplementary Fig. 11 shows that taking five random samples of 50% of the species of a growth form and calculating the suitability scores of the grid cells from each sample yields almost identical suitability scores. In most growth forms, even smaller subsets would produce identical results (Supplementary Fig. 8). The saturating curves in Supplementary Fig. 8 also show that the numbers of species used in this study were sufficient to characterize the climatic preferences of each growth form. An additional sensitivity analysis showed that the estimation of the ambient suitability surfaces was robust to excluding all species with less than 20 occurrence records (Supplementary Fig. 12).

Biases in the species distribution data and the use of modelled coarse (1 km resolution) climate forcing data may bias the parameterization of the TTR-SDM. Model comparisons, however, show that the TTR-SDM is less biased than correlative SDMs in predicting locations where the climate is suitable for a species^33,85^. Moreover, so long as these biases are not systematic, our procedure of averaging over many species models creates robust estimates of the growth-form suitability surfaces (Supplementary Figs. 8, 11 and 12).

### Data analysis

For each 25 km grid cell, we calculated the proportion of species of each growth form that can grow in the cell according to the fitted TTR-SDM. This proportion is interpreted as the climatic suitability of a grid cell for a growth form (Fig. 1).

To find groups of cells with similar suitabilities for different growth forms we used finite Gaussian mixture modelling as implemented in the R package mclust^93^. We refer to the geographic projection of these groups as phytoclimatic zones (Fig. 2a). We estimated the Bayesian information criterion (BIC) for different variations of the mclust algorithm, which revealed that the option ‘VEV’, allowing ellipsoidal, equally shaped clusters, consistently performed better than the alternatives. We used this option to model the clusters.

Using the number of (terrestrial) biomes delimited by global biome maps to guide the optimal number of clusters, one might delimit for instance 13 (ref. ^94^), 14 (ref. ^95^), 20 (refs.^96,97^), 21 (ref. ^98^) or 30 (ref. ^99^) clusters. We used 18 clusters to trade-off information content versus interpretability. The BIC of the VEV clustering models improved with the number of clusters used up to 30 clusters but indicated that the BIC of 18 clusters was not substantially lower than the maximum BIC at 30 clusters.

Our indices of novelty, disappearance and local change of phytoclimates, as well as the projections of shifts in phytoclimatic zones by 2070, are based on the multivariate Euclidean distances (ED) between ambient and future climatic suitabilities:

$${\rm{ED}}\left(i,j\right)=\sqrt{\mathop{\sum }\limits_{k=1}^{14}{\left({b}_{k,i}-{a}_{k,\,j}\right)}^{2}}$$

where *a*_*k*,*i*_ and *b*_*k*,*j*_ are the ambient and future suitabilities for growth form *k* in grid cells *i* and *j*, respectively. For within grid cell phytoclimatic change, *i* equals *j*. Note that the growth-form suitability values all range from 0 to 1. We calculated the novelty of the future phytoclimate in grid cell *x* by setting *i* = *x* and calculating ED(*i*,*j*) for *j* = 1 to *N*, where *N* is the total number of grid cells in the global 25 km grid. The minimum of these EDs is the distance of a future phytoclimate to its closest ambient analogue (ED_min_). A high ED_min_ thus indicates a high degree of novelty. Analogously, to determine phytoclimatic disappearance, we set *j* = *x* and calculate ED(*i*,*j*) for *i* = 1 to *N* and calculate the minimum of these EDs. Here, high values of ED_min_ indicate that an ambient phytoclimate has no close future analogue. Extended Data Figs. 3 and 4 show phytoclimatic change, novelty and loss for each GCM separately. Figure 3 shows the median values across the five projections per RCP.

To generate projections of shifts in phytoclimatic zones by 2070, we calculated the minimum ED of a future phytoclimate to its closest ambient analogue and extracted the ambient phytoclimatic zone of this closest ambient analogue. The future phytoclimate was then assigned to this phytoclimatic zone. Extended Data Fig. 5 shows shifts in phytoclimatic zones for each combination of RCP and GCM. Figure 2b in the main text shows the projection of zone shifts based on the median growth-form suitability values across the five projections for RCP 2.6 and Fig. 2c shows the same for RCP 8.5.

### Reporting summary

Further information on research design is available in the Nature Portfolio Reporting Summary linked to this article.

### Supplementary information

Supplementary Information Supplementary Figs. 1–12 and Tables 1–3.

Reporting Summary

## Data Availability

No new datasets were generated during the current study. The species distribution data were downloaded from the non-public version of the BIEN database v.4.1 (http://bien.nceas.ucsb.edu/bien/), which was made available to us upon request to BIEN. Plant trait and life-history data used to infer the growth forms of the species came from BIEN, GIFT^80^ (https://gift.uni-goettingen.de/home), the *Illustrated Handbook of Succulent Plants*^77–79^ and the global database of C_4_ photosynthesis in grasses^76^. Temperature and rainfall data were downloaded from CHELSA v.1.2 (https://chelsa-climate.org/), solar radiation data were downloaded from the Global Aridity and PET Database v.1 (https://csidotinfo.wordpress.com/data/global-aridity-and-pet-database/). Data on soil field capacity and wilting point came from the Global Gridded Surfaces of Selected Soil Characteristics (IGBP-DIS) dataset^62^ (10.3334/ORNLDAAC/569). Atmospheric CO_2_ concentrations were taken from ref. ^63^. Free vector data from www.naturalearthdata.com were used to create the background country maps.

## Code availability

R code to execute the TTR-SDM and a tutorial are available on Zenodo 10.5281/zenodo.10362617.
