# Supplementary material for: Reassessment of the risks of climate change for terrestrial ecosystems
Source: Nat Ecol Evol. 2024 Feb 26;8(5):888–900. doi: 10.1038/s41559-024-02333-8 (PMC11090816; doi:10.1038/s41559-024-02333-8)
Supplement: Supplementary file 1 — Supplementary Figs. 1–12 and Tables 1–3. [file 41559_2024_2333_MOESM1_ESM.pdf]

nature ecology & evolution

Article

https://doi.org/10.1038/s41559-024-02333-8

Reassessment of the risks of climate change for terrestrial ecosystems

In the format provided by the authors and unedited

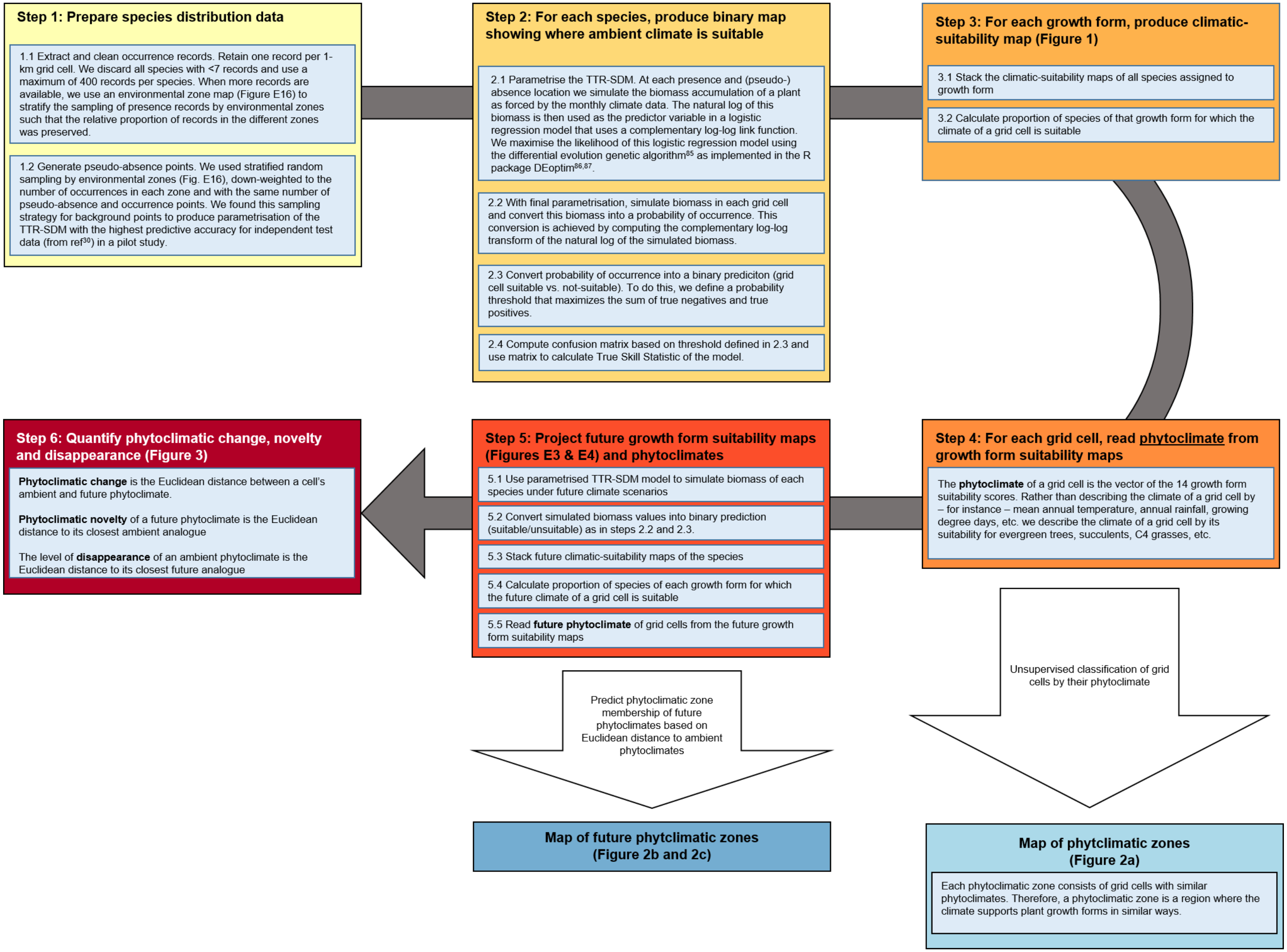

Supplementary Figure 1. Workflow for analyzing phytoclimates and their change. The original protocol is described in ref[24] of main text.

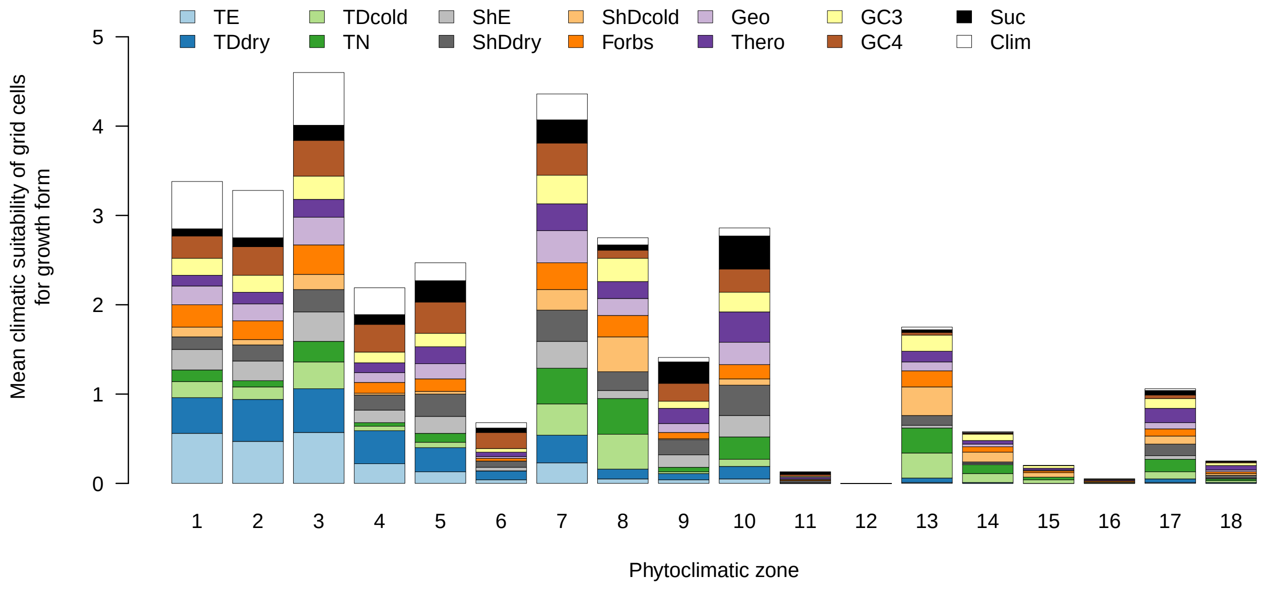

Supplementary Figure 2. Mean climatic suitability of grid cells for plant growth forms in phytoclimatic zones. The zone numbers are the same as in Fig. 2 of the main text. The values used to produce the figure are taken from Supplementary Table 1. TE=evergreen trees, TDdry=drought-deciduous trees, TDcold=cold-deciduous trees, TN=needleleaf trees, ShE=evergreen shrubs, ShDdry=drought-deciduous shrubs, ShDcold=cold-deciduous shrubs, Geo=geophytes, Thero=therophytes, GC3=C_3 grasses, GC4=C_4 grasses, Suc=succulents, Clim=climbers.

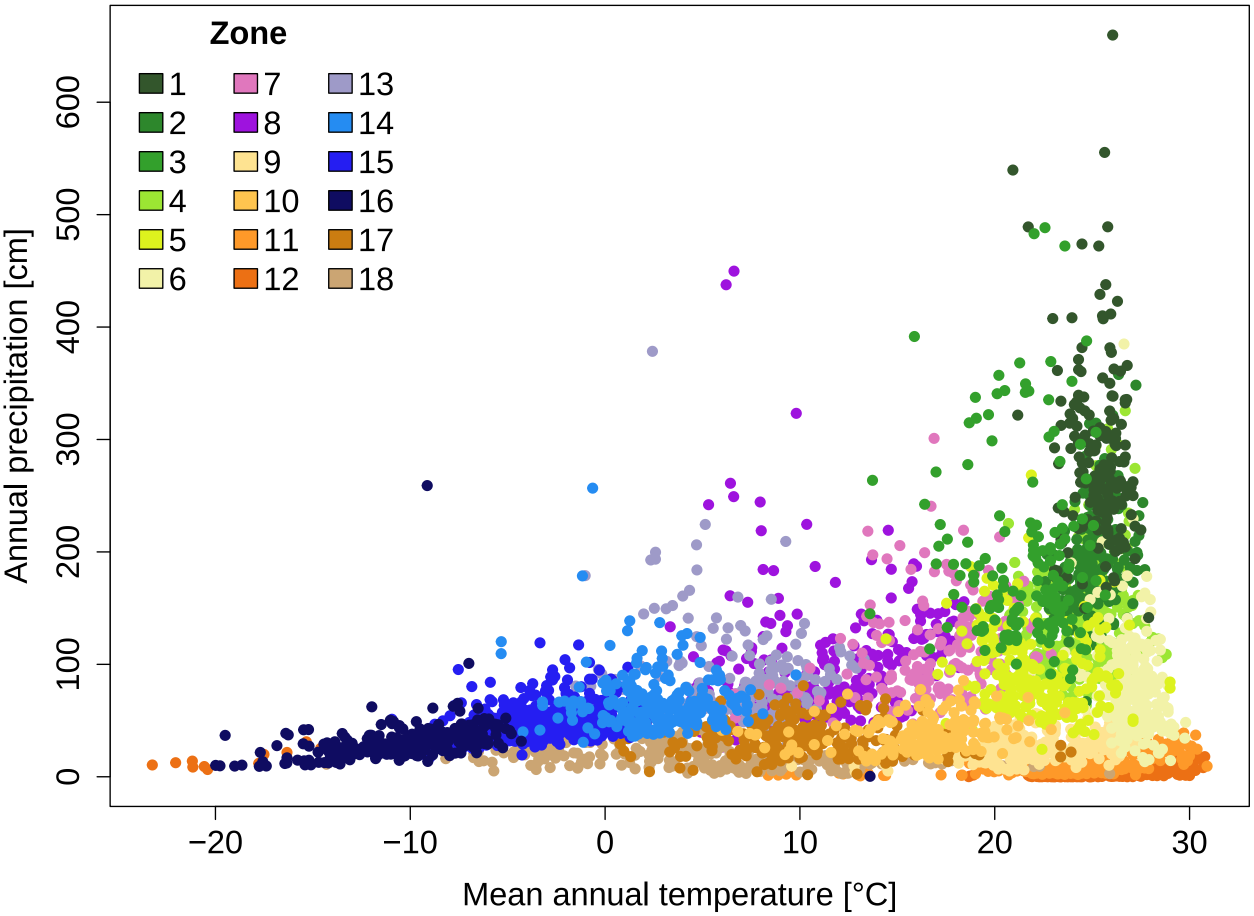

Supplementary Figure 3. Mean annual temperature and annual precipitation in the phytoclimatic zones. The figure uses a sample of 2.5% of the cells of each phytoclimatic zone. Zone 12 includes grid cells in the high arctic tundra and extreme dry and hot deserts, both of which have extremely low climatic suitability for all growth forms. Therefore, these cells are in the same phytoclimatic zone.

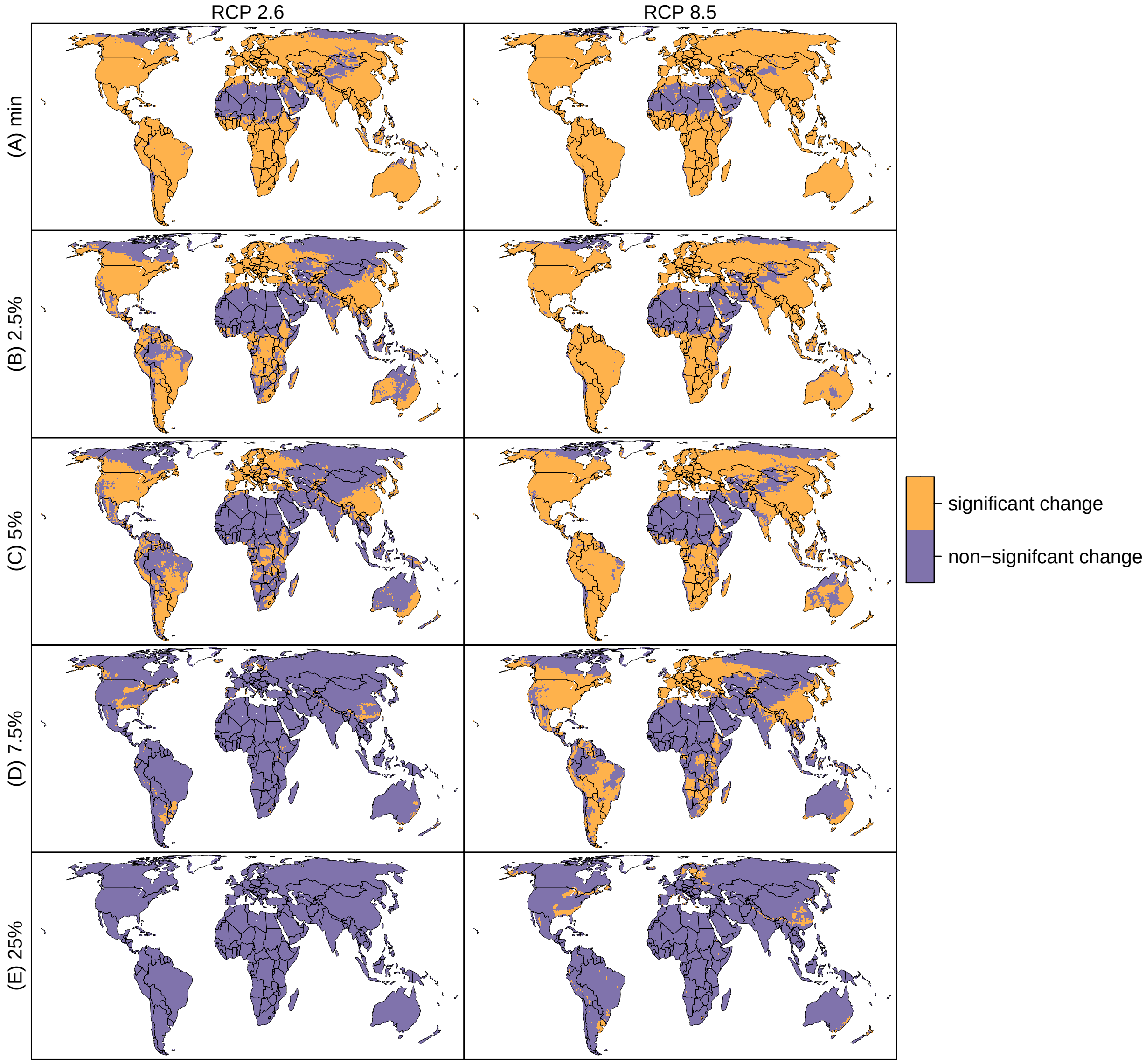

Supplementary Figure 4. Regions with significant phytoclimatic change when different threshold values for assessing significance are applied. The threshold values are (A) the distance between the centroids of the two most similar phytoclimatic zones in Euclidean growth form suitability space, (B) the 2.5th percentile, (C) the 5th percentile, (D) the 7.5th percentile, and (E) the 25th percentile of the pairwise inter-centroid distances between the phytolcimatic zones.

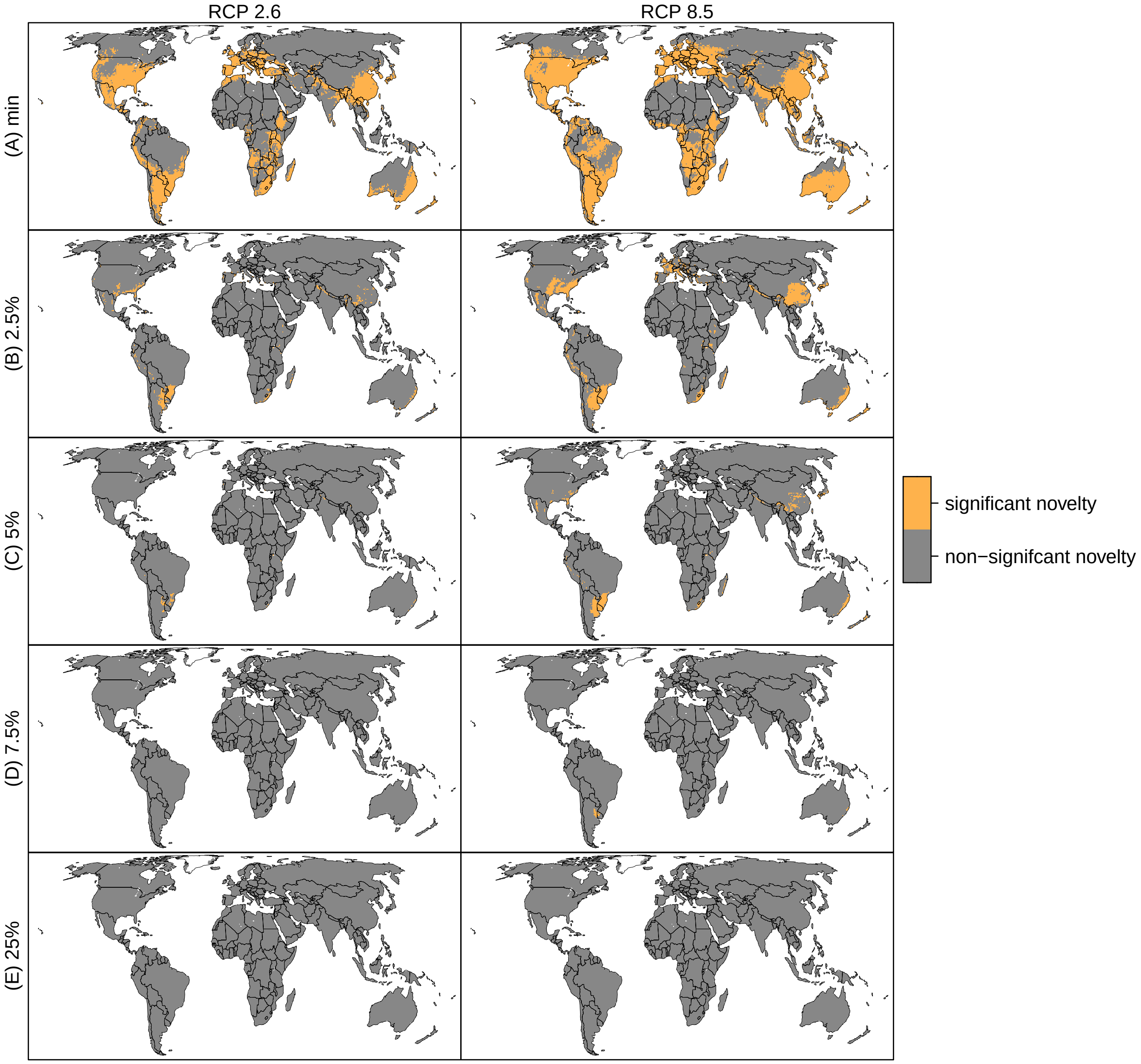

Supplementary Figure 5. Regions with significant phytoclimatic novelty when different threshold values for assessing significance are applied. The threshold values are (A) the distance between the centroids of the two most similar phytoclimatic zones in Euclidean growth form suitability space, (B) the 2.5th percentile, (C) the 5th percentile, (D) the 7.5th percentile, and (E) the 25th percentile of the pairwise inter-centroid distances between the phytolcimatic zones.

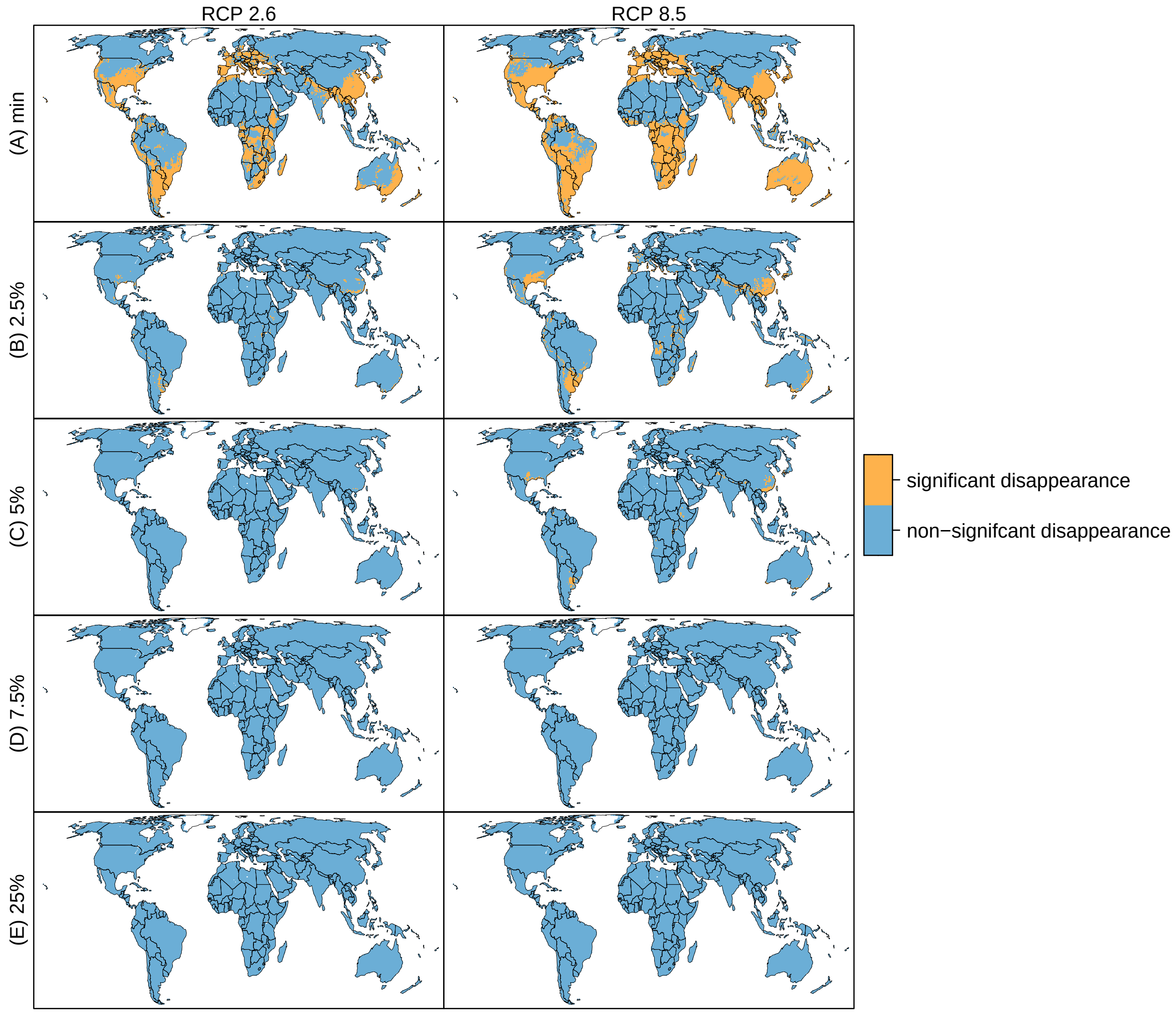

Supplementary Figure 6. Regions with significant disappearance of phytoclimate when different threshold values for assessing significance are applied. The threshold values are (A) the distance between the centroids of the two most similar phytoclimatic zones in Euclidean growth form suitability space, (B) the 2.5th percentile, (C) the 5th percentile, (D) the 7.5th percentile, and (E) the 25th percentile of the pairwise inter-centroid distances between the phytolcimatic zones.

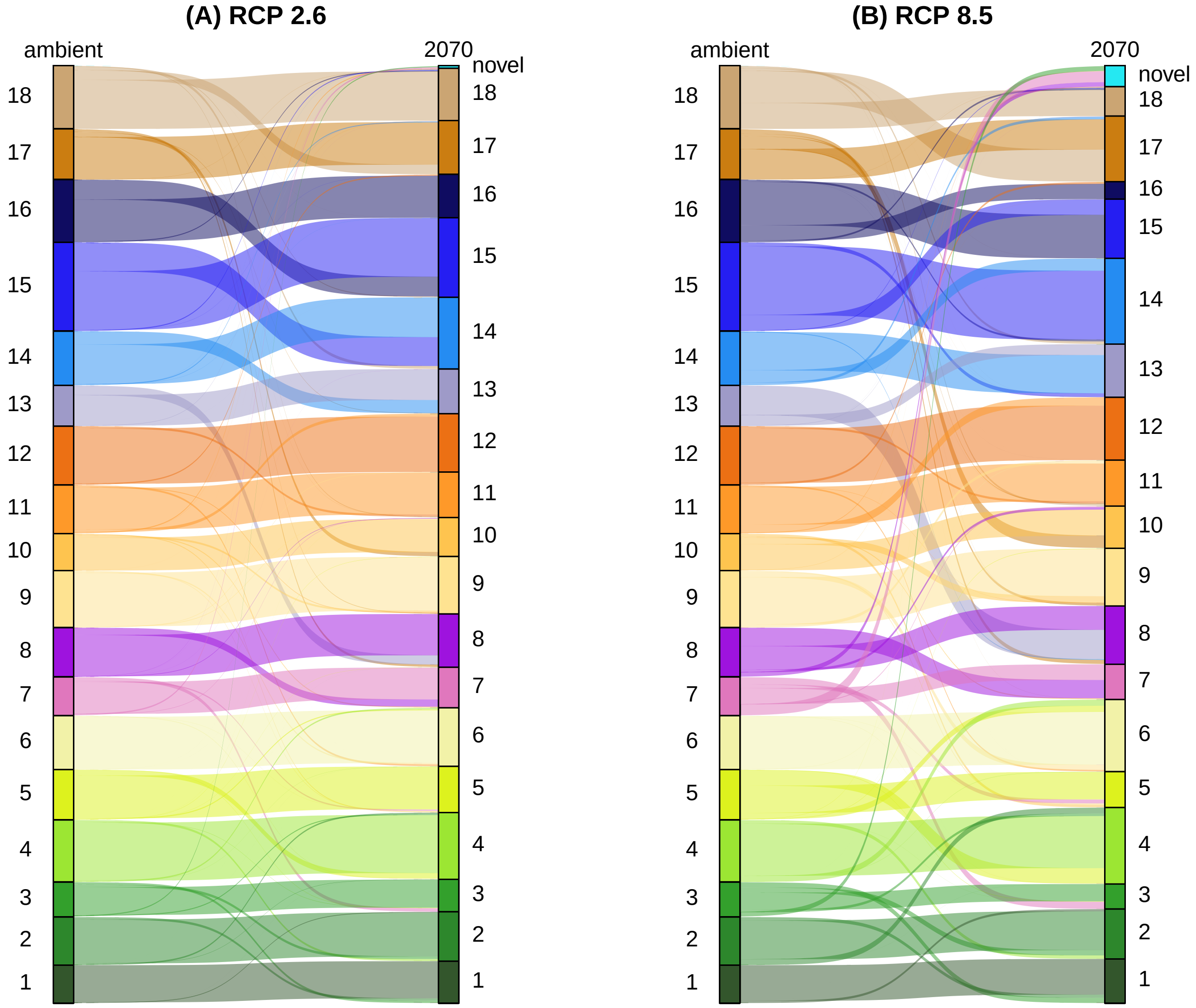

Supplementary Figure 7. Projected phytoclimatic zone transitions of grid cells by 2070 under (A) RCP 2.6 and (B) RCP 8.5. Zone numbers are the same as in Fig. 2 of the main text. The median growth form suitabilities of each grid cell in 2070 across projections from five Global Circulation were used to asses the future zone membership of each cell.

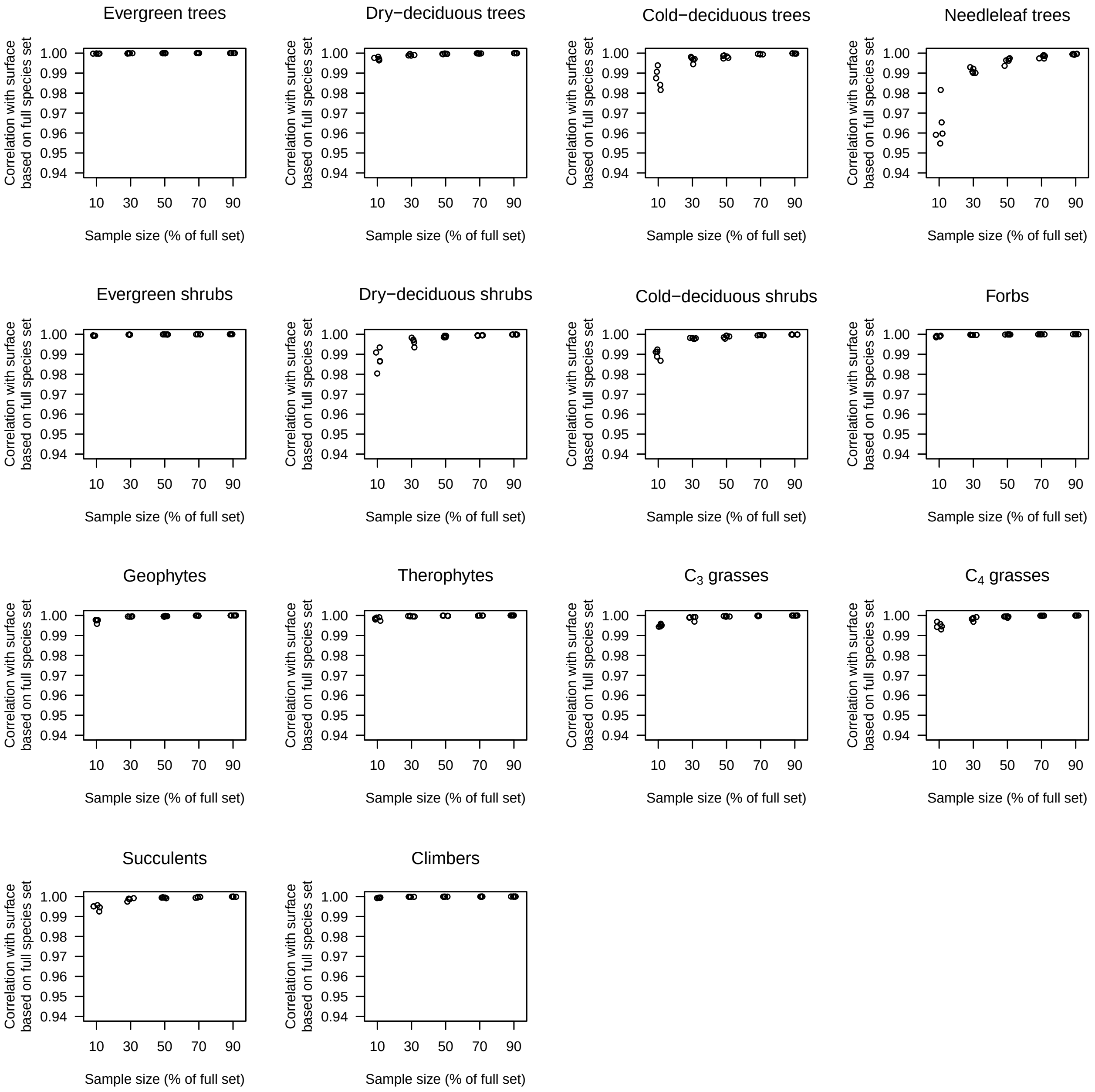

Supplementary Figure 8. Robustness of growth form suitability values to the number of species used to calculate growth form suitability. Each dot shows a Pearson correlation coefficient between the growth form suitability calculated from the full set of species of a growth form and the suitability calculated from one of five random subsets of species of that growth form. The size of the subsets is shown on the x-axis. Horizontal jitter was added to the dots to reduce overlap. The correlations with the full-set suitabilities already approximate 1 when smaller subsets are used, indicating that adding further species would not change the suitability estimates any further.

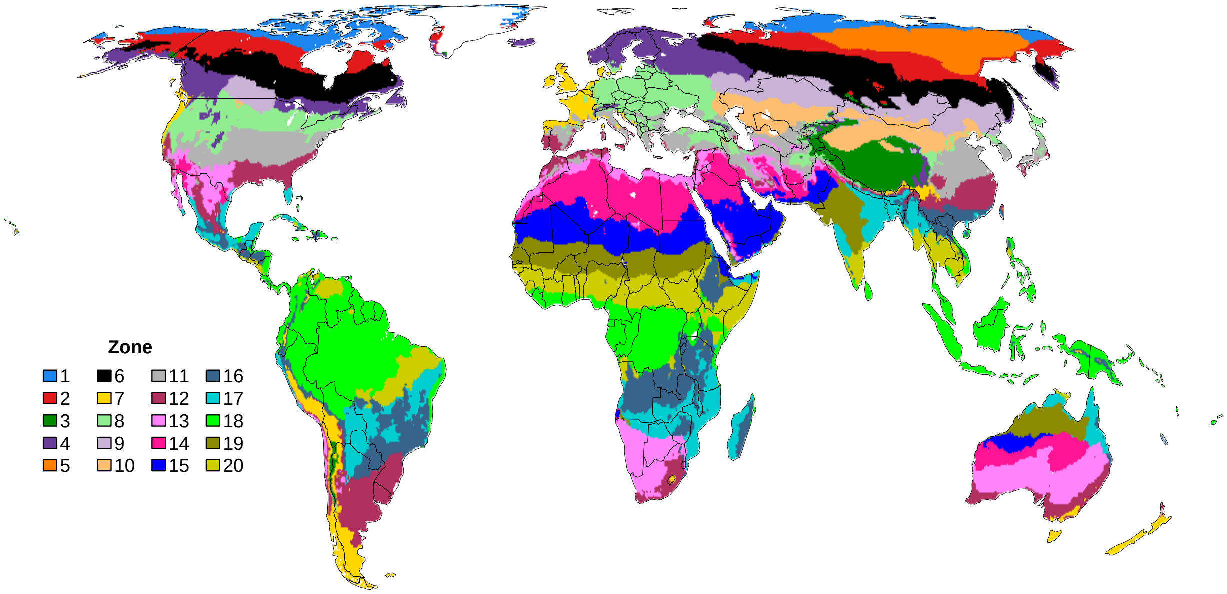

Supplementary Figure 9. Environmental zones used to stratify the sampling of presence and pseudo-absence points for species distribution modelling. The zones were obtained from a classification of grid cells based on the input variables of the TTR-SDM, i.e. monthly values of mean, maximum and minimum temperature, soil water content and solar radiation. The TTR-SDM also uses atmospheric CO_2 concentration, but CO_2 was assumed to be equally distributed globally and was thus not used in this classification of grid cells.

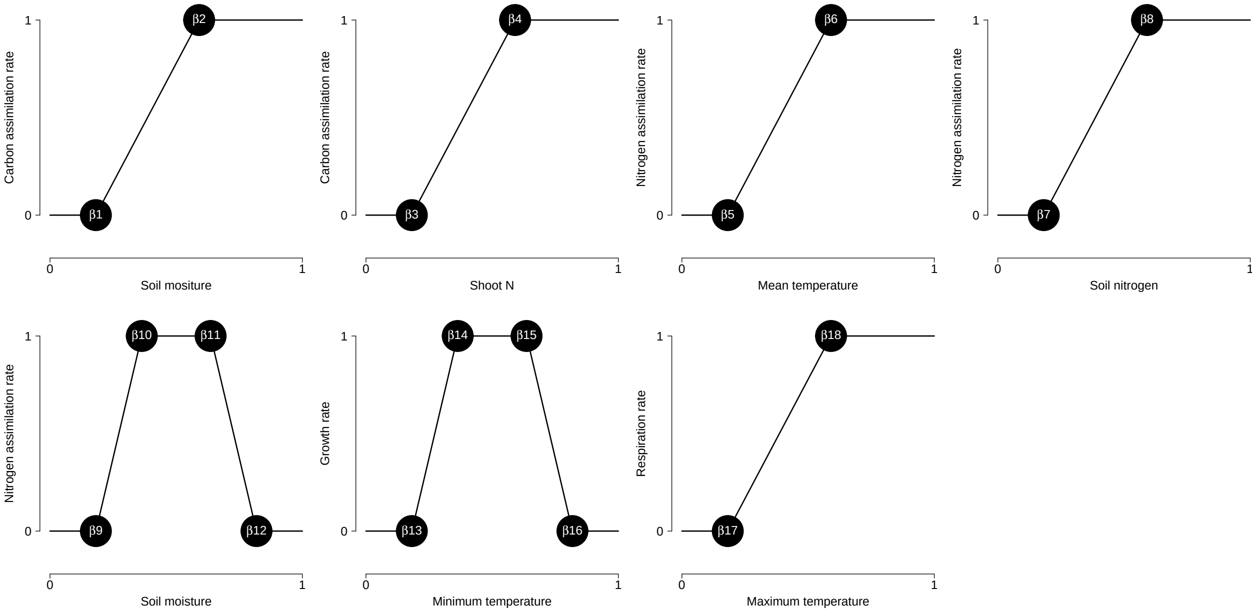

Supplementary Figure 10. Graphical representation of the parameters of TTR-SDM. The panels show the model's representation of how carbon and nitrogen uptake rates as well as growth and respiration rates are influenced by environmental variables. The x-axis coordinates of the 18 black dots are the parameters of the model. They vary among species and we estimate them from species distribution data. In addition, a Farquhar photosynthesis model is used to simulate how solar radiation, temperature and atmospheric CO_2 concentrations influence the monthly carbon uptake rates (ref[24] of main text). The Farquhar model used the same photosynthesis parameters for all C_3 and C_4 plants, respectively, and is therefore not shown. In this application of the TTR-SDM we assumed that soil nitrogen is spatially invariant, which means that nitrogen uptake rates are influenced by temperature and soil moisture only. This is appropriate when the goal is to estimate the climatic niche only. Note that carbon uptake is also influenced by shoot nitrogen (second panel), which is used as a proxy for leaf nitrogen, and that shoot nitrogen is not estimated from species distribution data, but modelled as a function of the plant's nitrogen pools as described in ref[32] of main text. Also note that the numbering of the parameters differs from ref[32] due to differences in how carbon uptake is modelled.

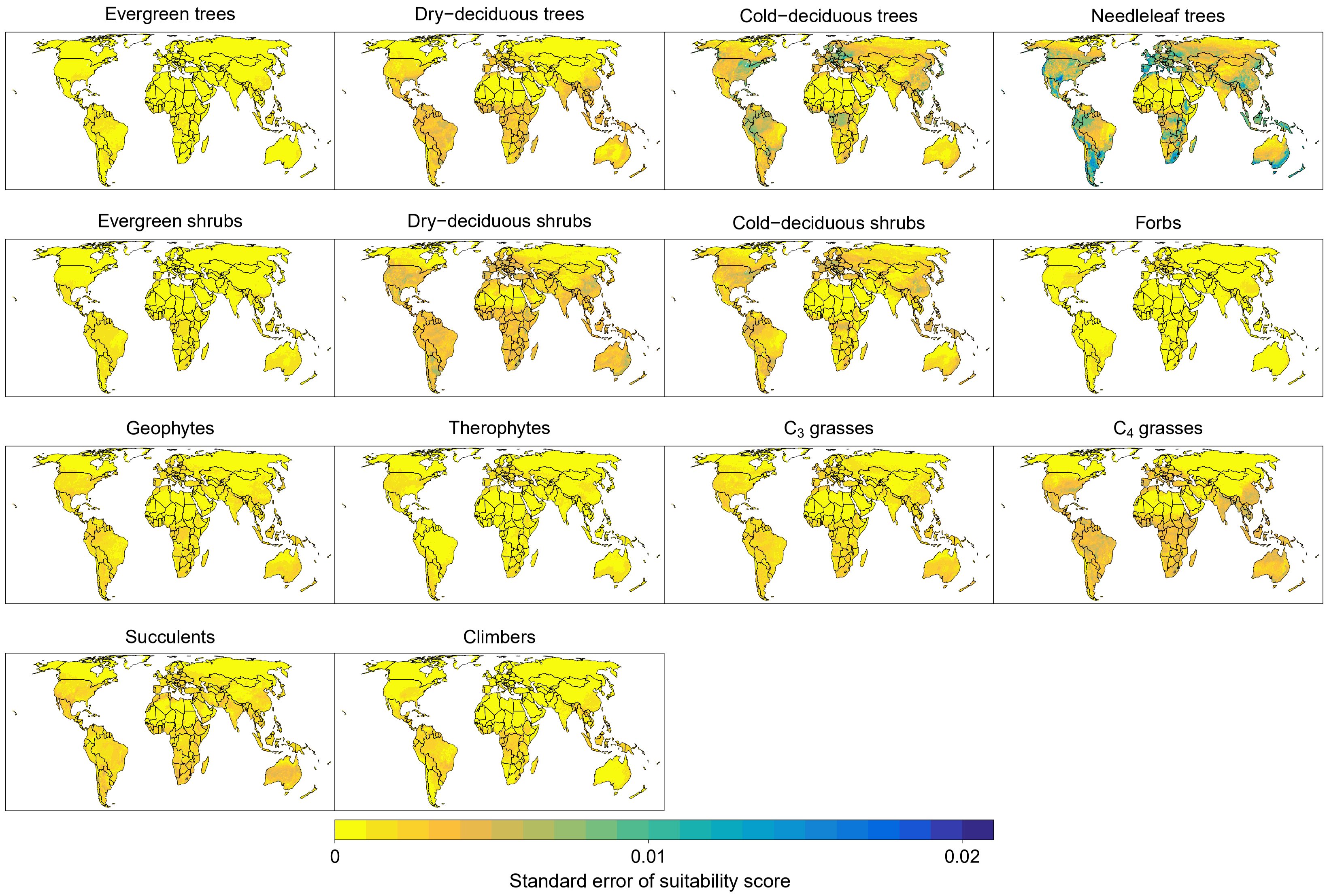

Supplementary Figure 11. Standard error of ambient suitability values calculated from five random subsets of 50% of the species from each growth form. The suitability values can range between 0 and 1. The values of the standard errors therefore indicate that the suitability estimate are accurate. The highest standard errors occur in the needleleaf trees, the total number of which was 439.

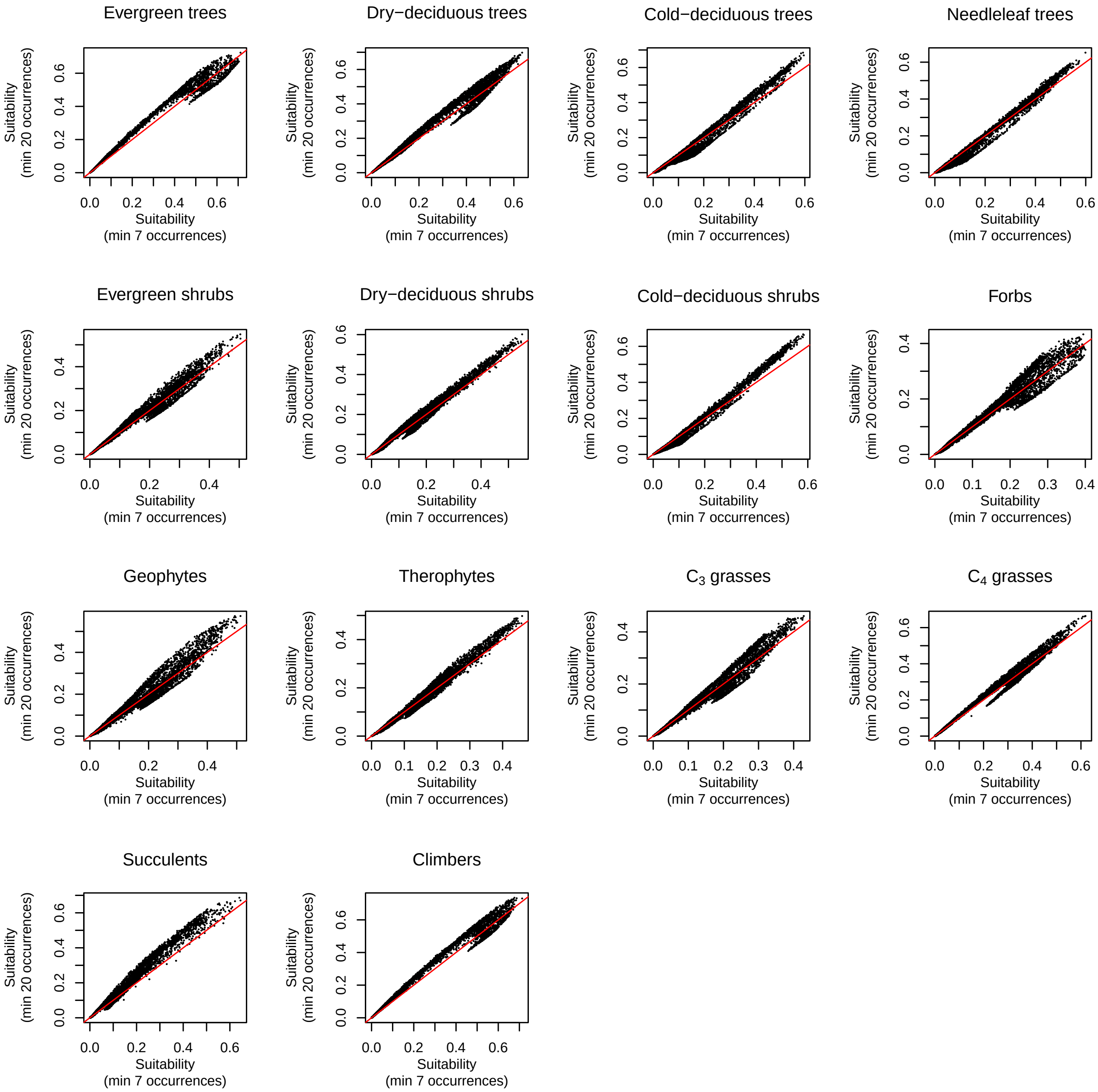

Supplementary Figure 12. **Modelled climatic suitability of ambient grid cells for plant growth forms derived from range models with a minimum of seven versus 20 occurrence records.**

Supplementary Table 1. Mean climatic suitability of grid cells for plant growth forms in phytoclimatic zones. The zone numbers are the same as in Fig. 2 of the main text. Standard errors of the means were always ≤0.002. TE=evergreen trees, TDdry=drought-deciduous trees, TDcold=cold-deciduous trees, TN=needleleaf trees, ShE=evergreen shrubs, ShDdry=drought-deciduous shrubs, ShDcold=cold-deciduous shrubs, Geo=geophytes, Thero=therophytes, GC3=C_3 grasses, GC4=C_4 grasses, Suc=succulents, Clim=climbers.

Zone	TE	TDdry	TDcold	TN	ShE	ShDdry	ShDcold	Forbs	Geo	Thero	GC3	GC4	Suc	Clim
1	0.56	0.40	0.18	0.13	0.23	0.14	0.11	0.25	0.21	0.12	0.19	0.25	0.08	0.53
2	0.47	0.47	0.14	0.07	0.22	0.18	0.06	0.21	0.19	0.13	0.19	0.32	0.10	0.53
3	0.57	0.49	0.30	0.23	0.33	0.25	0.17	0.33	0.31	0.20	0.26	0.40	0.17	0.59
4	0.22	0.37	0.05	0.04	0.14	0.17	0.02	0.12	0.11	0.11	0.12	0.31	0.11	0.30
5	0.13	0.27	0.06	0.10	0.19	0.25	0.03	0.14	0.17	0.19	0.15	0.35	0.24	0.20
6	0.04	0.10	0.00	0.00	0.04	0.07	0.00	0.03	0.02	0.05	0.04	0.18	0.05	0.06
7	0.23	0.31	0.35	0.40	0.30	0.35	0.23	0.30	0.36	0.30	0.32	0.36	0.26	0.29
8	0.05	0.11	0.39	0.40	0.09	0.21	0.39	0.24	0.19	0.19	0.26	0.09	0.06	0.08
9	0.04	0.07	0.02	0.05	0.14	0.17	0.01	0.07	0.10	0.17	0.08	0.20	0.24	0.05
10	0.05	0.14	0.08	0.25	0.24	0.34	0.07	0.16	0.25	0.34	0.22	0.26	0.37	0.09
11	0.00	0.00	0.00	0.00	0.01	0.02	0.00	0.01	0.01	0.02	0.01	0.02	0.03	0.00
12	0.00	0.00	0.00	0.00	0.00	0.00	0.00	0.00	0.00	0.00	0.00	0.00	0.00	0.00
13	0.01	0.05	0.28	0.28	0.03	0.11	0.32	0.18	0.10	0.12	0.18	0.03	0.03	0.03
14	0.00	0.01	0.10	0.10	0.01	0.02	0.11	0.06	0.03	0.04	0.07	0.01	0.01	0.01
15	0.00	0.00	0.04	0.03	0.00	0.00	0.05	0.02	0.01	0.02	0.03	0.00	0.00	0.00
16	0.00	0.00	0.01	0.00	0.00	0.00	0.01	0.01	0.00	0.01	0.01	0.00	0.00	0.00
17	0.01	0.04	0.08	0.14	0.04	0.13	0.09	0.08	0.07	0.16	0.11	0.04	0.05	0.02
18	0.00	0.01	0.02	0.02	0.01	0.03	0.02	0.02	0.02	0.05	0.03	0.01	0.01	0.00

Supplementary Table 2: Reference list for BIEN occurrence records.

Anderson, J., Eckhart, V. & Geber, M. Data from: Experimental studies of adaptation in Clarkia xantiana. III. Phenotypic selection across a subspecies border. (2015) doi:10.5061/dryad.b631d.
Anderson-Teixeira, K. J. et al. CTFS-ForestGEO: a worldwide network monitoring forests in an era of global change. Global Change Biology 21, 528-549 (2015).
Aussenac, R. et al. Data from: Intraspecific variability in growth response to environmental fluctuations modulates the stabilizing effect of species diversity on forest growth. (2016) doi:10.5061/dryad.55gb7.
Benkman, C. Data from: The natural history of the South Hills crossbill in relation to its impending extinction. (2016) doi:10.5061/dryad.cm586.
Bhattarai, G. et al. Data from: Biogeography of a plant invasion: genetic variation and plasticity in latitudinal clines for traits related to herbivory. (2016) doi:10.5061/dryad.r8d1m.
Blonder, B. et al. The leaf-area shrinkage effect can bias paleoclimate and ecology research. American Journal of Botany 99, 1756-1763 (2012).
Bonal, D., Sabatier, D., Montpied, P., Tremeaux, D. & Guehl, J.-M. Interspecific variability of d13 C among trees in rainforests of French Guiana: functional groups and canopy integration. Oecologia 124, 454-468 (2000).
Caughlin, T. et al. Data from: A hyperspectral image can predict tropical tree growth rates in single-species stands. (2016) doi:10.5061/dryad.t6md2.
Duwyn, A. & MacDougall, A. Data from: When anthropogenic-related disturbances overwhelm demographic persistence mechanisms. (2015) doi:10.5061/dryad.km53t.
Enquist, B. & Boyle, B. SALVIAS - the SALVIAS vegetation inventory database. Biodiversity & Ecology 4, 288-288 (2012).
Fegraus, E. Tropical Ecology Assessment and Monitoring Network (TEAM Network). Biodivers. Ecol. 4, 287-287 (2012).
Forest Inventory and Analysis National Program. (2013). http://www.fia.fs.fed.us/ [accessed 19 January 2013]
Forsyth, D. et al. Data from: Density-dependent effects of a widespread invasive herbivore on tree survival and biomass during reforestation. (2015) doi:10.5061/dryad.2q242.
Frainer, A. et al. Data from: Stoichiometric imbalances between detritus and detritivores are related to shifts in ecosystem functioning. (2015) doi:10.5061/dryad.dm94c.
Gapare, W. Data from: Genetic parameters in subtropical pine F1 hybrids: heritabilities, between-trait correlations and genotype-by-environment interactions. (2015) doi:10.5061/dryad.d5672.
GBIF Occurrence Download https://doi.org/10.15468/dl.yubndf [accessed 17 May 2018]
Han, W., Fang, J., Guo, D. & Zhang, Y. Leaf nitrogen and phosphorus stoichiometry across 753 terrestrial plant species in China. New Phytologist 168, 377-385 (2005).
Karagatzides, J. & Ellison, A. Construction Costs of Carnivorous Plants and Non-Carnivorous Plants. Harvard Forest Data Archive: HF112 (2008).
Kraft, N. J., Valencia, R. & Ackerly, D. D. Functional traits and niche-based tree community assembly in an Amazonian forest. Science 322, 580-582 (2008).
Kraft, T. et al. Data from: Seed size and the evolution of leaf defences. (2015) doi:10.5061/dryad.69ph0.
Lawson, J., Fryirs, K. & Leishman, M. Data from: Hydrological conditions explain wood density in riparian plants of south-eastern Australia. (2015) doi:10.5061/dryad.72h45.
Lett, S., Nilsson, M., Hegethorn, Wardle, D. & Dorrepaal, E. Data from: Bryophyte traits explain climate-warming effects on tree seedling establishment. (2016) doi:10.5061/dryad.kv145.

Li, W., Xu, F., Zheng, S., Taube, F. & Bai, Y. Data from: Patterns and thresholds of grazing-induced changes in community structure and ecosystem functioning: species-level responses and the critical role of species traits. (2016) doi:10.5061/dryad.9n859.

Li, X., Schmid, B., Wang, F. & Paine, C. Data from: Net assimilation rate determines the growth rates of 14 species of subtropical forest trees. (2016) doi:10.5061/dryad.5kb61.

Loughnan, D. & Gilbert, B. Data from: Trait-mediated community assembly: distinguishing the signatures of biotic and abiotic filters. (2017) doi:10.5061/dryad.512p5.

Meers, T. L., Kasel, S., Bell, T. L. & Enright, N. J. Conversion of native forest to exotic Pinus radiata plantation: Response of understorey plant composition using a plant functional trait approach. Forest Ecology and Management 259, 399-409 (2010).

Mitchell, N. et al. Data from: Functional traits in parallel evolutionary radiations and trait-environment associations in the Cape Floristic region of South Africa. (2014) doi:10.5061/dryad.sc286.3.

Molinari, N. & D'Antonio, C. Data from: Structural, compositional and trait differences between native and non-native dominated grassland patches. (2013) doi:10.5061/dryad.50hd2.

Mottet, M., DeBlois, J. & Perron, M. Data from: High genetic variation and moderate to high values for genetic parameters of Picea abies resistance to Pissodes strobi. (2015) doi:10.5061/dryad.pq075.

Niu, K., He, J. & Lechowicz, M. Data from: Grazing-induced shifts in community functional composition and soil nutrient availability in Tibetan alpine meadows. (2016) doi:10.5061/dryad.r5m20.

Ostevik, K., Andrew, R., Otto, S. & Rieseberg, L. Data from: Multiple reproductive barriers separate recently diverged sunflower ecotypes. (2016) doi:10.5061/dryad.223p4.

Peet, R. K. et al. Vegetation-plot database of the Carolina Vegetation Survey. Biodiversity and Ecology 4, 243-253 (2012).

Peet, R. K., Lee, M. T., Jennings, M. D. & Faber-Langendoen, D. VegBank: a permanent, open-access archive for vegetation plot data. Biodivers. Ecol. 4, 233-241 (2012).

Peppe, D. et al. Data from: Biomechanical and leaf-climate relationships: a comparison of ferns and seed plants. (2014) doi:10.5061/dryad.528td.

Pluchon, N., Gundale, M., Nilsson, M., Kardol, P. & Wardle, D. Data from: Stimulation of boreal tree seedling growth by wood-derived charcoal: effects of charcoal properties, seedling species and soil fertility. (2013) doi:10.5061/dryad.gd48h.

Poorter, L. & Bongers, F. Leaf traits are good predictors of plant performance across 53 rain forest species. Ecology 87, 1733-1743 (2006).

Poorter, L. & Rozendaal, D. M. Leaf size and leaf display of thirty-eight tropical tree species. Oecologia 158, 35-46 (2008).

Poorter, L. The Relationships of Wood-, Gas- and water fractions of tree stems to performance and life history variation in tropical trees. Annals of Botany 102, 367 (2008).

Red Mundial De Informacion Sobre Biodiversidad .(2008). URL url{http://www.conabio.gob.mx/remib_ingles/doctos/remib_ing.html} [accessed 27 March 2012]

Salgado-Luarte, C. & Gianoli, E. Data from: Herbivores modify selection on plant functional traits in a temperate rainforest understory. (2012) doi:10.5061/dryad.53tr05j2.

Schreiber, S., Hacke, U. & Hamann, A. Data from: Variation of xylem vessel diameters across a climate gradient: insight from a reciprocal transplant experiment with a widespread boreal tree. (2015) doi:10.5061/dryad.jn81h.

Sessa, E. & Givnish, T. Data from: Leaf form and photosynthetic physiology of Dryopteris species distributed along light gradients in eastern North America. (2013) doi:10.5061/dryad.38h06.

Shibata, R. et al. Data from: Relationships between resprouting ability, species traits, and resource allocation patterns in woody species in a temperate forest. (2015) doi:10.5061/dryad.rj480.
Spasojevic, M., Turner, B. & Myers, J. Data from: When does intraspecific trait variation contribute to functional beta-diversity? (2015) doi:10.5061/dryad.rr4pm.
SpeciesLink. (2012). http://www.splink.org.br/ [accessed 29 March 2012]
Steane, D. et al. Data from: Genome-wide scans reveal cryptic population structure in a dry-adapted eucalypt. (2015) doi:10.5061/dryad.h06r3.
Teegalapalli, K. & Datta, A. Data from: Field to a forest: patterns of forest recovery following shifting cultivation in the eastern Himalaya. (2016) doi:10.5061/dryad.k83h6.
Urrutia-Jalabert, R., Malhi, Y. & Lara, A. Data from: The oldest, slowest forests in the world? Exceptional biomass and slow carbon dynamics of Fitzroya cupressoides temperate rainforests in southern Chile. (2015) doi:10.5061/dryad.2kh91.
Wheelwright, N., Sinclair, J., Hochwender, C. & Janzen, F. Data from: Leaf size in three generations of a dioecious tropical tree, Ocotea tenera (Lauraceae): sexual dimorphism and changes with age. (2012) doi:10.5061/dryad.271cs.
Wigley, B. et al. Data from: Leaf traits of African woody savanna species across climate and soil fertility gradients: evidence for conservative vs. acquisitive resource use strategies. (2016) doi:10.5061/dryad.v240b.
Wright, I. J. et al. The worldwide leaf economics spectrum. Nature 428, 821-827 (2004).
Wright, S., Sun, I., Pickering, M., Fletcher, C. & Chen, Y. Data from: Long-term changes in liana loads and tree dynamics in a Malaysian forest. (2015) doi:10.5061/dryad.dh439.
Yang, X., Huang, Z., Zhang, K. & Cornelissen, J. Data from: C:N:P stoichiometry of Artemisia species and close relatives across northern China: unraveling effects of climate, soil and taxonomy. (2015) doi:10.5061/dryad.5r776.
Yoder, J. et al. Data from: Effects of gene flow on phenotype matching between two varieties of Joshua tree (Yucca brevifolia; Agavaceae) and their pollinators. (2013) doi:10.5061/dryad.369q9.
Zas, R. & Sampedro, L. Data from: Heritability of seed weight in Maritime pine, a relevant trait in the transmission of environmental maternal effects. (2014) doi:10.5061/dryad.60j76.
Zerebecki, R., Crutsinger, G. & Hughes, A. Data from: Spartina alterniflora genotypic identity affects plant and consumer responses in an experimental marsh community. (2016) doi:10.5061/dryad.h32p6.
Zheng, S. et al. Effects of grazing on leaf traits and ecosystem functioning in Inner Mongolia grasslands: scaling from species to community. Biogeosciences 7, 1117-1132 (2010).
Zhu, H. et al. Data from: Reducing soil erosion by improving community functional diversity in semi-arid grasslands. (2015) doi:10.5061/dryad.b5tr9.

Supplementary Table 3: Reference list for BIEN trait data.

Abbasi, A. M., Shah, M. H. & Khan, M. A. Wild edible vegetables of lesser Himalayas. (Springer, 2015).
Abera, B. Plants used in material culture in Oromo community, Jimma, Southwest Oromia, Ethiopia. African Journal of Plant Science 7, 285–299 (2013).
Addo-Fordjour, P., Obeng, S., Anning, A. & Addo, M. Floristic composition, structure and natural regeneration in a moist semi-deciduous forest following anthropogenic disturbances and plant invasion. International Journal of Biodiversity and Conservation 1, 021–037 (2009).
Afolayan, A. J., Grierson, D. S. & Mbeng, W. O. Ethnobotanical survey of medicinal plants used in the management of skin disorders among the Xhosa communities of the Amathole District, Eastern Cape, South Africa. Journal of Ethnopharmacology 153, 220–232 (2014).
Agarwal, S., Madan, U., Chinnamani, S. & Rege, N. Ecological studies in the Nilgiris. Indian Forester 87, 376–389 (1961).
Agenbag, L., Elser, K., Midgley, G. & Boucher, C. Diversity and species turnover on an altitudinal gradient in Western Cape, South Africa: baseline data for monitoring range shifts in response to climate change. Bothalia 38, (2008).
Agoramoorthy, G., Chen, F.-A., Venkatesalu, V., Kuo, D.-H. & Shea, P.-C. Evaluation of antioxidant polyphenols from selected mangrove plants of India. Asian Journal of Chemistry 20, 1311 (2008).
Ahluwalia, K. Contribution to the flora of Mount Abu. Indian Forester 91, 824–828 (1965).
Ahmad, L. et al. Ethnopharmacological documentation of medicinal plants used for hypertension among the local communities of DIR Lower, Pakistan. Journal of Ethnopharmacology 175, 138–146 (2015).
Aizen, M. A. & Feinsinger, P. Forest fragmentation, pollination, and plant reproduction in a Chaco dry forest, Argentina. Ecology 75, 330–351 (1994).
Al-Adhroey, A. H., Nor, Z. M., Al-Mekhlafi, H. M. & Mahmud, R. Ethnobotanical study on some Malaysian anti-malarial plants: A community based survey. Journal of Ethnopharmacology 132, 362–364 (2010).
Aldhebiani, A. & Howladar, S. Floristic diversity and environmental relations in two valleys, south west Saudi Arabia. International Journal of Science and Research 4, 1916–1925 (2013).
Alemayehu, G., Asfaw, Z. & Kelbessa, E. Ethnobotanical study of medicinal plants used by local communities of Minjar-Shenkora District, North Shewa Zone of Amhara Region, Ethiopia. Journal of Medicinal Plants Studies 3, 01–11 (2015).
Aljaro, M. & Montenegro, G. Growth of dominant Chilean shrubs in the Andean cordillera. Mountain Research and Development 287–291 (1981).
Allen, R., Partridge, T., Lee, W. & Efford, M. Ecology of Kunzea ericoides (A. Rich.) J. Thompson (kanuka) in east Otago, New Zealand. New Zealand Journal of Botany 30, 135–149 (1992).
Amenu, E. Use and management of medicinal plants by indigenous people of Ejaji area (chelya woreda) west shoa, Ethiopia: An ethnobotanical approach. School of Graduate Studies, Addis Araba Universiti, Ethiopia (2007).
Anning, A. & Yeboah-Gyan, K. Diversity and distribution of invasive weeds in Ashanti Region, Ghana. African Journal of Ecology 45, 355–360 (2007).
Anning, A. et al. Floristic composition and vegetation structure of the KNUST Botanic Garden, Kumasi, Ghana. Journal of Science and Technology (Ghana) 28, 103–122 (2008).
Antos, J. A. & Zobel, D. B. Habitat relationships of Chamaecyparis nootkatensis in southern Washington, Oregon, and California. Canadian Journal of Botany 64, 1898–1909 (1986).

Antos, J. A. & Zobel, D. B. Plant form, developmental plasticity, and survival following burial by volcanic tephra. Canadian Journal of Botany 63, 2083–2090 (1985).

Araújo, F. S. de et al. Floristics and life-forms along a topographic gradient, central-western Ceará, Brazil. Rodriguésia 62, 341–366 (2011).

Arianoutsou, M. Aspects of demography in post-fire Mediterranean plant communities of Greece. in Landscape disturbance and biodiversity in Mediterranean-Type ecosystems 273–295 (Springer, 1998).

Asase, A. & Asafo-Agyei, T. Plants used for treatment of malaria in communities around the Bobiri forest reserve in Ghana. Journal of Herbs, Spices & Medicinal Plants 17, 85–106 (2011).

Asase, A. & Oppong-Mensah, G. Traditional antimalarial phytotherapy remedies in herbal markets in southern Ghana. Journal of Ethnopharmacology 126, 492–499 (2009).

Asase, A., Akwetey, G. A. & Achel, D. G. Ethnopharmacological use of herbal remedies for the treatment of malaria in the Dangme West District of Ghana. Journal of Ethnopharmacology 129, 367–376 (2010).

Asensio, N., Cristobal-Azkarate, J., Dias, P. A. D., Vea, J. J. & Rodríguez-Luna, E. Foraging habits of Alouatta palliata mexicana in three forest fragments. Folia Primatologica 78, 141–153 (2007).

Asfaw, Z. & Tadesse, M. Prospects for sustainable use and development of wild food plants in Ethiopia. Economic Botany 55, 47–62 (2001).

Astaras, C. & Waltert, M. What does seed handling by the drill tell us about the ecological services of terrestrial cercopithecines in African forests? Animal Conservation 13, 568–578 (2010).

Au, A. Y., Corlett, R. T. & Hau, B. C. Seed rain into upland plant communities in Hong Kong, China. Plant Ecology 186, 13–22 (2006).

Austrheim, G. et al. Large scale experimental effects of three levels of sheep densities on an alpine ecosystem. Oikos 117, 837–846 (2008).

Ayodele, M. Karyomorphological studies in some Nigerian species of Vernonia Schreb.(Asteraceae) with different growth forms. Feddes Repertorium 110, 541–553 (1999).

Baard, J. & Kraaij, T. Alien flora of the Garden Route National Park, South Africa. South African Journal of Botany 94, 51–63 (2014).

Baird, K. J. & Rieger, J. P. A restoration design for least Bell's vireo habitat in San Diego County. (1989).

Bamidele, J., Dania, O. & Oyomoare, L. Plant genetic resources of home gardens in three ecological zones of Nigeria. African Scientist 12, 89–98 (2011).

Bannister, P. Godley review: a touch of frost? Cold hardiness of plants in the southern hemisphere. New Zealand Journal of Botany 45, 1–33 (2007).

Baruch, Z. & Goldstein, G. Leaf construction cost, nutrient concentration, and net CO_2 assimilation of native and invasive species in Hawaii. Oecologia 121, 183–192 (1999).

Basabose, A. K. Diet composition of chimpanzees inhabiting the montane forest of Kahuzi, Democratic Republic of Congo. American Journal of Primatology 58, 1–21 (2002).

Batanouny, K., Stichler, W. & Ziegler, H. Photosynthetic pathways and ecological distribution of Euphorbia species in Egypt. Oecologia 87, 565–569 (1991).

Batanouny, K., Stichler, W. & Ziegler, H. Photosynthetic pathways and ecological distribution ofEuphorbia species in Egypt. Oecologia 87, 565–569 (1991).

Beaulieu, J. M., Leitch, I. J., Patel, S., Pendharkar, A. & Knight, C. A. Genome size is a strong predictor of cell size and stomatal density in angiosperms. New Phytologist 179, 975–986 (2008).

Beccaloni, G. W. Vertical stratification of ithomiine butterfly (Nymphalidae: Ithomiinae) mimicry complexes: the relationship between adult flight height and larval host-plant height. Biological Journal of the Linnean Society 62, 313–341 (1997).

Becerra, A., Cabello, M. & Chiarini, F. Arbuscular mycorrhizal colonization of vascular plants from the Yungas forests, Argentina. Annals of Forest Science 64, 765–772 (2007).

Becker, T. & Müller, J. V. Floristic affinities, life-form spectra and habitat preferences of the vegetation of two semi-arid regions in Sahelian West and Southern Africa. Basic and Applied Dryland Research 1, 33–50 (2007).

Beentje, H. Botanical assessment of Ngezi forest, Pemba. (Finnish Forest and Park Service, 1993).

Behailu, E. Ethnobotanical study of traditional medicinal plants of Goma Wereda, Jima zone of Oromia region, Ethiopia. (2010).

Bein, E., Habte, B., Jaber, A., Birnie, A. & Tengnäs, B. Useful trees and shrubs in Eritrea: identification, propagation and management for agricultural and pastoral communities. Technical handbook 422 (1996).

Belayneh, A. & Bussa, N. F. Ethnomedicinal plants used to treat human ailments in the prehistoric place of Harla and Dengego valleys, eastern Ethiopia. Journal of ethnobiology and ethnomedicine 10, 18 (2014).

Bell, T. & Adams, M. Attack on all fronts: functional relationships between aerial and root parasitic plants and their woody hosts and consequences for ecosystems. Tree Physiology 31, 3–15 (2011).

Bellingham, P. J. & Sparrow, A. D. Resprouting as a life history strategy in woody plant communities. Oikos 89, 409–416 (2000).

Benzing, D. H. Vascular epiphytes: general biology and related biota. (Cambridge University Press, 2008).

Bergh, N. G. Systematics of the Relhaniinae (Asteraceae-Gnaphalieae) in southern Africa: geography and evolution in an endemic Cape plant lineage. (University of Cape Town, 2009).

Beukes, P. & Ellis, F. Soil and vegetation changes across a Succulent Karoo grazing gradient. African Journal of Range and Forage Science 20, 11–19 (2003).

Bharali, S., Paul, A., Khan, M. L. & Singha, L. B. Species diversity and community structure of a temperate mixed Rhododendron forest along an altitudinal gradient in West Siang District of Arunachal Pradesh, India. Nature and Science 9, 125–140 (2011).

Bhatnagar, H. & others. Plant communities in some Sal (Shorea robusta) forests of UP. Indian Forester 86, 139–51 (1960).

Bilbrough, C. J., Welker, J. M. & Bowman, W. D. Early spring nitrogen uptake by snow-covered plants: a comparison of arctic and alpine plant function under the snowpack. Arctic, Antarctic, and Alpine Research 404–411 (2000).

Biswas, S. et al. Compositions, distributions and status of economic plants among invasive floras of Uttarpara, West Bengal, India. International Journal of Pharmacognosy 1, 800–809 (2014).

Bliss, B. J. Investigating evolution of plant development in basal angiosperms. (The Pennsylvania State University, 2008).

Bliss, L. & Cantlon, J. Succession on river alluvium in northern Alaska. American Midland Naturalist 452–469 (1957).

Boakye, M., Little, I., Panagos, M., Jansen, R., & others. Effects of burning and grazing on plant species percentage cover and habitat condition in the highland grassland of Mpumalanga Province, South Africa. Journal of Animal & Plant Sciences 23, 603–610 (2013).

Böhle, U.-R., Hilger, H. H. & Martin, W. F. Island colonization and evolution of the insular woody habit in Echium L.(Boraginaceae). Proceedings of the National Academy of Sciences 93, 11740–11745 (1996).

Bollen, A. & Elsacker, L. V. Feeding ecology of Pteropus rufus (Pteropodidae) in the littoral forest of Sainte Luce, SE Madagascar. Acta Chiropterologica 4, 33–47 (2002).

Bongers, F., Parren, M. P. & Traoré, D. Forest climbing plants of West Africa: diversity, ecology and management. (CABI, 2005).

Bork, E. W., Hudson, R. J. & Bailey, A. W. Upland plant community classification in Elk Island National Park, Alberta, Canada, using disturbance history and physical site factors. Plant Ecology 130, 171–190 (1997).

Boulter, S., McDonald, W. J., Kitching, R., Zalucki, J. & Jessup, L. W. Plant reproductive phenology and floral resources of an Australian subtropical rainforest. (2011).

Brundrett, M. C. & Abbott, L. Roots of jarrah forest plants. I. Mycorrhizal associations of shrubs and herbaceous plants. Australian Journal of Botany 39, 445–457 (1991).

Brundu, G., Aksoy, N., Brunel, S., Eliáš, P. & Fried, G. Rapid surveys for inventorying alien plants in the Black Sea region of Turkey. EPPO Bulletin 41, 208–216 (2011).

Bruyns, P. V., Mapaya, R. J. & Hedderson, T. J. A new subgeneric classification for Euphorbia (Euphorbiaceae) in southern Africa based on ITS and psbA-trnH sequence data. Taxon 55, 397–420 (2006).

Burke, A. Floristic relationships between inselbergs and mountain habitats in the central Namib. Dinteria 28, 19–38 (2003).

Burke, A. From plains to inselbergs: species in special habitats as indicators for climate change? Journal of Biogeography 31, 831–841 (2004).

Burke, A. Plant endemism in the central Namib Desert. Evolutionary Ecology Research 9, 283–297 (2007).

Burke, A. Recovery in naturally dynamic environments: a case study from the Sperrgebiet, Southern African arid succulent karoo. Environmental management 40, 635–648 (2007).

Burrows, C. J. Processes of vegetation change. in Processes of vegetation change 359–419 (Springer, 1990).

Callaghan, T. & Emanuelsson, U. Population structure and processes of tundra plants and vegetation. in The population structure of vegetation 399–439 (Springer, 1985).

Carbutt, C. The emerging invasive alien plants of the Drakensberg Alpine Centre, southern Africa. Bothalia 42, 71–85 (2012).

Carnegie, A. J., Bi, H., Arnold, S., Li, Y. & Binns, D. Distribution, host preference, and impact of parasitic mistletoes (Loranthaceae) in young eucalypt plantations in New South Wales, Australia This article is one of a collection of papers based on a presentation from the Stem and Shoot Fungal Pathogens and Parasitic Plants: the Values of Biological Diversity session of the XXII International Union of Forestry Research Organization World Congress meeting held in Brisbane, Queensland, Australia, in 2005. Botany 87, 49–63 (2009).

Carrick, P. J. & Ezcurra, E. Competitive and facilitative relationships among three shrub species, and the role of browsing intensity and rooting depth in the Succulent Karoo, South Africa. Journal of Vegetation Science 14, 761–772 (2003).

Casper, B. The application of sex allocation theory to heterostylous plants. in Evolution and function of heterostyly 209–223 (Springer, 1992).

ÇATAV, Ş. S. et al. Germination response of five eastern Mediterranean woody species to smoke solutions derived from various plants. Turkish Journal of Botany 36, 480–487 (2012).

Cavieres, L. A., Badano, E. I., Sierra-Almeida, A., Gómez-González, S. & Molina-Montenegro, M. A. Positive interactions between alpine plant species and the nurse cushion plant Laretia acaulis do not increase with elevation in the Andes of central Chile. New Phytologist 169, 59–69 (2006).

Cebrian, J. & Duarte, C. The dependence of herbivory on growth rate in natural plant communities. Functional Ecology 518–525 (1994).

Cernusak, L. A., Pate, J. S. & Farquhar, G. D. Oxygen and carbon isotope composition of parasitic plants and their hosts in southwestern Australia. Oecologia 139, 199–213 (2004).

Chandrasekharan, C. Forest types of Kerala state. Indian Forester 88, 660–674 (1962).

Chapin III, F. S., Johnson, D. A. & McKendrick, J. D. Seasonal movement of nutrients in plants of differing growth form in an Alaskan tundra ecosystem: implications for herbivory. The Journal of Ecology 189–209 (1980).
Charudattan, R. Ecological, practical, and political inputs into selection of weed targets: What makes a good biological control target? Biological Control 35, 183–196 (2005).
Chaudhri, A. Common Grasses and Sedges of Kurseong, Kalimpong and Darjeeling Forest Divisions, West Bengal. Indian Forester 86, 336–353 (1960).
Chaudhuri, A. A Preliminary Report on the Ferns of West Bengal,-Part Sikkim Forests and some Aspects of their Ecology. Indian Forester 90, 95–104 (1964).
Chavan, A. & Padate, S. Contribution to the Flora of Savali Taluka. Indian Forester 86, 210–219 (1960).
Chen, S., Bai, Y., Lin, G., Liang, Y. & Han, X. Effects of grazing on photosynthetic characteristics of major steppe species in the Xilin River Basin, Inner Mongolia, China. Photosynthetica 43, 559–565 (2005).
Cheng, X. et al. Summer rain pulse size and rainwater uptake by three dominant desert plants in a desertified grassland ecosystem in northwestern China. Plant Ecology 184, 1–12 (2006).
Chettri, A., Barik, S. K., Pandey, H. N. & Lyngdoh, M. K. Liana diversity and abundance as related to microenvironment in three forest types located in different elevational ranges of the Eastern Himalayas. Plant Ecology & Diversity 3, 175–185 (2010).
Chinsembu, K., Hijarunguru, A. & Mbangu, A. Ethnomedicinal plants used by traditional healers in the management of HIV/AIDS opportunistic diseases in Rundu, Kavango East Region, Namibia. South African Journal of Botany 100, 33–42 (2015).
Chowdhury, M. S. H. & Koike, M. Towards exploration of plant-based ethno-medicinal knowledge of rural community: basis for biodiversity conservation in Bangladesh. New Forests 40, 243–260 (2010).
Cole, D. N. Experimental trampling of vegetation. II. Predictors of resistance and resilience. Journal of Applied Ecology 215–224 (1995).
Coley, P. Effects of plant growth rate and leaf lifetime on the amount and type of anti-herbivore defense. Oecologia 74, 531–536 (1988).
Collier, M. H., Vankat, J. L. & Hughes, M. R. Diminished plant richness and abundance below Lonicera maackii, an invasive shrub. The American Midland Naturalist 147, 60–71 (2002).
Comerford, S. C. Medicinal plants of two mayan healers from San Andrés, Petén, Guatemala. Economic Botany 50, 327–336 (1996).
Cornelissen, J. H. C., Diez, P. C. & Hunt, R. Seedling Growth, Allocation and Leaf Attributes in a Wide Range of Woody Plant Species and Types. Journal of Ecology 84, 755–765 (1996).
Corns, I. G. & Roi, G. H. L. A comparison of mature with recently clear-cut and scarified lodgepole pine forests in the Lower Foothills of Alberta. Canadian Journal of Forest Research 6, 20–32 (1976).
Cortés-Flores, J., Andresen, E., Cornejo-Tenorio, G. & Ibarra-Manríquez, G. Fruiting phenology of seed dispersal syndromes in a Mexican Neotropical temperate forest. Forest Ecology and Management 289, 445–454 (2013).
Costion, C. M., Kitalong, A. H. & Holm, T. Plant endemism, rarity, and threat in Palau, Micronesia: a geographical checklist and preliminary Red List assessment. Micronesica 41, 131–164 (2009).
Cowling, R. & Witkowski, E. Convergence and non-convergence of plant traits in climatically and edaphically matched sites in Mediterranean Australia and South Africa. Austral Ecology 19, 220–232 (1994).

Cowling, R., Mustart, P., Laurie, H. & Richards, M. Species diversity; functional diversity and functional redundancy in fynbos communities. South African Journal of Science 90, 333–337 (1994).
Cowling, R., Pierce, S., Stock, W. & Cocks, M. Why are there so many myrmecochorous species in the Cape fynbos? in Plant-animal interactions in Mediterranean-type ecosystems 159–168 (Springer, 1994).
Cronk, Q. C. & Fuller, J. L. Plant invaders: the threat to natural ecosystems. (Routledge, 2014).
Cruz-Garcia, G. S. & Price, L. L. Ethnobotanical investigation of'wild'food plants used by rice farmers in Kalasin, Northeast Thailand. Journal of ethnobiology and ethnomedicine 7, 33 (2011).
Csurhes, S. & Edwards, R. National weeds program, potential environmental weeds in Australia, Candidate species for preventative control. National Parks and Wildlife Biodiversity Group, Environment Australia, Canberra: 208pp (1998).
De, J. Further observations on the ethnobotany of Purulia District in West Bengal. Indian Forester 95, 551–559 (1969).
Deb, D. & Dutta, R. A new species of Mycetia Reinw. from Assam. Indian Forester 91, 272–274 (1965).
Deb, D. Plants new to India. Indian Forester 91, 193–194 (1965).
den Dubbelden, K. C. & Verburg, R. W. Inherent allocation patterns and potential growth rates of herbaceous climbing plants. Plant and Soil 184, 341–347 (1996).
Deng, F., Zang, R. & Chen, B. Identification of functional groups in an old-growth tropical montane rain forest on Hainan Island, China. Forest Ecology and Management 255, 1820–1830 (2008).
Devar, A. T. Eucalyptus macrorrhyncha in the Nilgiri and the Palani Hills of South India-an Important Source for Rutin. Indian Forester 95, 473–474 (1969).
Dey, A., Saxena, H. & Uniyal, M. Botanical exploration in the Bhagirathi valley with particular reference to the medicinal plants. Indian Forester 95, 190–207 (1969).
Diame, G. Ethnobotany and ecological studies of plants used for reproductive health: a case study at Bia biosphere reserve in the western region of Ghana. Department of Environmental Science, University of Cape Coast, Ghana (2010).
Dickie, I. A. et al. Belowground legacies of Pinus contorta invasion and removal result in multiple mechanisms of invasional meltdown. AoB Plants 6, plu056 (2014).
Diemer, M. Microclimatic convergence of high-elevation tropical páramo and temperate-zone alpine environments. Journal of Vegetation Science 7, 821–830 (1996).
Dorrepaal, E., Cornelissen, J. H., Aerts, R., Wallen, B. & Van Logtestijn, R. S. Are growth forms consistent predictors of leaf litter quality and decomposability across peatlands along a latitudinal gradient? Journal of Ecology 93, 817–828 (2005).
Downey, P. O. An inventory of host species for each aerial mistletoe species (Loranthaceae and Viscaceae) in Australia. Cunninghamia 5, 685–720 (1998).
Downey, P. O. et al. Does the tolerance of weeds to herbicide change with elevated levels of CO2. in Proceedings of the 18th Australasian Weeds Conference" Developing Solutions to Evolving Weed Problems", Melbourne, Victoria, Australia 273–274 (2012).
Du Rietz, G. E. Life-forms of terrestrial flowering plants. (Almqvist & Wiksell, 1931).
du Toit, J. C., van den Berg, L. & O'Connor, T. G. Fire effects on vegetation in a grassy dwarf shrubland at a site in the eastern Karoo, South Africa. African Journal of Range & Forage Science 32, 13–20 (2015).
Dulin, M. W. & Kirchoff, B. K. Paedomorphosis, secondary woodiness, and insular woodiness in plants. The Botanical Review 76, 405–490 (2010).
Duncan, R. S. & Chapman, C. A. Seed dispersal and potential forest succession in abandoned agriculture in tropical Africa. Ecological Applications 9, 998–1008 (1999).

Edlund, S. A. & Alt, B. T. Regional congruence of vegetation and summer climate patterns in the Queen Elizabeth Islands, Northwest Territories, Canada. Arctic 3–23 (1989).

Ehleringer, J. & Marshall, J. Water relations. Parasitic plants 125–140 (1995).

Ehleringer, J. et al. Xylem-tapping mistletoes: water or nutrient parasites. Science 227, 1479–1481 (1985).

Ehrenfeld, J. G. Wetlands of the New Jersey Pine Barrens: the role of species composition in community function. American Midland Naturalist 301–313 (1986).

Ehrlén, J. & Eriksson, O. Toxicity in Fleshy Fruits: A Non-Adaptive Trait? Oikos 107–113 (1993).

El-Ghani, M. A., El-Kheir, M. A., Abdel-Dayem, M. & El-Hamid, M. A. Vegetation analysis and soil characteristics of five common desert climbing plants in Egypt. Turkish Journal of Botany 35, 561–580 (2011).

Eliáš, P. Some ecophysiological features in leaves of plants in an oak-hornbeam forest. Folia Geobotanica et Phytotaxonomica 14, 29–42 (1979).

El-Sheikh, M. Plant succession on abandoned fields after 25 years of shifting cultivation in Assuit, Egypt. Journal of Arid Environments 61, 461–481 (2005).

Emms, J., Virtue, J. G., Preston, C. & Bellotti, W. D. Legumes in temperate Australia: A survey of naturalisation and impact in natural ecosystems. Biological Conservation 125, 323–333 (2005).

Ewers, F. W., Fisher, J. B. & Chiu, S.-T. A survey of vessel dimensions in stems of tropical lianas and other growth forms. Oecologia 84, 544–552 (1990).

Fang, Z., Bao, W., Yan, X. & Liu, X. Understory structure and vascular plant diversity in naturally regenerated deciduous forests and spruce plantations on similar clear-cuts: implications for forest regeneration strategy selection. Forests 5, 715–743 (2014).

Farnsworth, E., Nunez-Farfan, J., Careaga, S. & Bazzaz, F. Phenology and growth of three temperate forest life forms in response to artificial soil warming. Journal of Ecology 967–977 (1995).

Feinsinger, P., Beach, J. H., Linhart, Y. B., Busby, W. H. & Murray, K. G. Disturbance, pollinator predictability, and pollination success among Costa Rican cloud forest plants. Ecology 68, 1294–1305 (1987).

Field, C. Climate and the food habits of ungulates on Galana Ranch. African Journal of Ecology 13, 203–220 (1975).

Filgueiras, T. S. Herbaceous plant communities. The Cerrados of Brazil: ecology and natural history of a neotropical savanna 121–139 (2002).

Finlayson, C. M., Bailey, B. & Cowie, I. Macrophyte vegetation of the Magela Creek flood plain, Alligator Rivers Region, Northern Territory. (AGPS, 1989).

Fioeoen, E. Medicinal plants in China containing pyrrolizidine alkaloids. Pharmazie 55, I0 (2000).

Fleishman, E. et al. Effects of floristics, physiognomy and non-native vegetation on riparian bird communities in a Mojave Desert watershed. Journal of Animal Ecology 72, 484–490 (2003).

Flores, J. & Briones, O. Plant life-form and germination in a Mexican inter-tropical desert: effects of soil water potential and temperature. Journal of arid environments 47, 485–497 (2001).

Fonda, R. Forest succession in relation to river terrace development in Olympic National Park, Washington. Ecology 55, 927–942 (1974).

Fontenla, S., Puntieri, J. & Ocampo, J. Mycorrhizal associations in the Patagonian steppe, Argentina. Plant and Soil 233, 13–29 (2001).

Fonty, É., Sarthou, C., Larpin, D. & PONGE, J.-F. A 10- year decrease in plant species richness on a neotropical inselberg: detrimental effects of global warming? Global Change Biology 15, 2360–2374 (2009).

Forbis, T. A. Seedling demography in an alpine ecosystem. American Journal of Botany 90, 1197–1206 (2003).

Foster, S. & Janson, C. H. The relationship between seed size and establishment conditions in tropical woody plants. Ecology 66, 773–780 (1985).
Fox, J. F. Responses of diversity and growth-form dominance to fertility in Alaskan tundra fellfield communities. Arctic and Alpine Research 233–237 (1992).
Frantzen, N. & Bouman, F. Dispersal and growth form patterns of some zonal páramo vegetation types. Acta botanica neerlandica 38, 449–465 (1989).
Freitag, H. & Maier-Stolte, M. The genus Ephedra in NE tropical Africa. Kew Bulletin 415–426 (2003).
Frelich, L. E., Machado, J.-L. & Reich, P. B. Fine-scale environmental variation and structure of understorey plant communities in two old-growth pine forests. Journal of Ecology 91, 283–293 (2003).
Fu, H., Pei, S., Chen, Y. & Wan, C. Influence of shrubs on soil chemical properties in Alxa desert steppe, China. (2007).
Funk, J. L. & Throop, H. L. Enemy release and plant invasion: patterns of defensive traits and leaf damage in Hawaii. Oecologia 162, 815–823 (2010).
Gaertner, M., Den Breeyen, A., Hui, C. & Richardson, D. M. Impacts of alien plant invasions on species richness in Mediterranean-type ecosystems: a meta-analysis. Progress in Physical Geography 33, 319–338 (2009).
Gajaseni, J. & Gajaseni, N. Ecological rationalities of the traditional homegarden system in the Chao Phraya Basin, Thailand. Agroforestry Systems 46, 3–23 (1999).
Galabuzi, C. Conservation and local utilization of key anti-malarial medicinal plants in the Sango Bay area, southern Uganda. (2008).
Galindo-González, J., Guevara, S. & Sosa, V. J. Bat-and bird-generated seed rains at isolated trees in pastures in a tropical rainforest. Conservation biology 14, 1693–1703 (2000).
Gallagher, R. V., Beaumont, L. J., Hughes, L. & Leishman, M. R. Evidence for climatic niche and biome shifts between native and novel ranges in plant species introduced to Australia. Journal of Ecology 98, 790–799 (2010).
Gallego-Fernández, J. B. & Martínez, M. L. Environmental filtering and plant functional types on Mexican foredunes along the Gulf of Mexico. (2011).
Gallenmüller, F., Rowe, N. & Speck, T. Development and growth form of the neotropical liana Croton nuntians: the effect of light and mode of attachment on the biomechanics of the stem. Journal of plant growth regulation 23, 83–97 (2004).
Galmés, J. G. Ecophysiological traits and their responses to drought in species from the Balearic Islands with different growth forms. (Universitat de les Illes Balears, 2006).
Galmés, J., Abadía, A., Cifre, J., Medrano, H. & Flexas, J. Photoprotection processes under water stress and recovery in Mediterranean plants with different growth forms and leaf habits. Physiologia Plantarum 130, 495–510 (2007).
Galmés, J., Flexas, J., Savé, R. & Medrano, H. Water relations and stomatal characteristics of Mediterranean plants with different growth forms and leaf habits: responses to water stress and recovery. Plant and Soil 290, 139–155 (2007).
Ganapathy, P. & Rangarajan, M. A Study of Phenology and Nursery Behaviour of Andaman Timber Species. Indian Forester 90, 758–766 (1964).
Gashaw, M. et al. Post-fire regeneration strategies and tree bark resistance to heating in frequently burning tropical savanna woodlands and grasslands in Ethiopia. Nordic Journal of Botany 22, 19–33 (2002).
Gashaw, M., Michelsen, A., Jensen, M. & Friis, I. Soil seed bank dynamics of fire-prone wooded grassland, woodland and dry forest ecosystems in Ethiopia. Nordic Journal of Botany 22, 5–17 (2002).
Gatsuk, L., Smirnova, O., Vorontzova, L., Zaugolnova, L. & Zhukova, L. Age states of plants of various growth forms: a review. The Journal of Ecology 675–696 (1980).

Gehrke, B., Kandziora, M. & Pirie, M. D. The evolution of dwarf shrubs in alpine environments: a case study of Alchemilla in Africa. Annals of Botany mcv159 (2015).

Geldenhuys, C. J. Native forest regeneration in pine and eucalypt plantations in Northern Province, South Africa. Forest Ecology and Management 99, 101–115 (1997).

Gemedo-Dalle, T., Maass, B. L. & Isselstein, J. Plant biodiversity and ethnobotany of Borana pastoralists in Southern Oromia, Ethiopia. Economic Botany 59, 43–65 (2005).

Ghaderian, S. & Baker, A. Geobotanical and biogeochemical reconnaissance of the ultramafics of Central Iran. Journal of Geochemical Exploration 92, 34–42 (2007).

Gholz, H. L., Grier, C., Campbell, A., Brown, A., & others. Equations for estimating biomass and leaf area of plants in the Pacific Northwest. (1979).

Ghorbani, A., Langenberger, G., Feng, L. & Sauerborn, J. Ethnobotanical study of medicinal plants utilised by Hani ethnicity in Naban river watershed national nature reserve, Yunnan, China. Journal of Ethnopharmacology 134, 651–667 (2011).

Ghosh, R. Teak Plantations of North Bengal. Indian Forester 91, 83–92 (1965).

Giday, M., Asfaw, Z. & Woldu, Z. Medicinal plants of the Meinit ethnic group of Ethiopia: an ethnobotanical study. Journal of Ethnopharmacology 124, 513–521 (2009).

Giday, M., Teklehaymanot, T., Animut, A. & Mekonnen, Y. Medicinal plants of the Shinasha, Agew-awi and Amhara peoples in northwest Ethiopia. Journal of Ethnopharmacology 110, 516–525 (2007).

Ginocchio, R. & Montenegro, G. Interpretation of metameric architecture in dominant shrubs of the Chilean matorral. Oecologia 90, 451–456 (1992).

Givnish, T. Leaf mottling: relation to growth form and leaf phenology and possible role as camouflage. Functional Ecology 463–474 (1990).

Glasgow, L. S. & Matlack, G. R. Prescribed burning and understory composition in a temperate deciduous forest, Ohio, USA. Forest Ecology and Management 238, 54–64 (2007).

Glatzel, G. & Geils, B. Mistletoe ecophysiology: host–parasite interactions This review is one of a collection of papers based on a presentation from the Stem and Shoot Fungal Pathogens and Parasitic Plants: the Values of Biological Diversity session of the XXII International Union of Forestry Research Organization World Congress meeting held in Brisbane, Queensland, Australia, in 2005. Botany 87, 10–15 (2008).

Gnanasekaran, G., Nehru, P. & Narasimhan, D. Angiosperms of Sendirakillai Sacred Grove (SSG), Cuddalore District, Tamil Nadu, India. Check List 8, 113–129 (2012).

Gómez-González, S., Sierra-Almeida, A. & Cavieres, L. Does plant-derived smoke affect seed germination in dominant woody species of the Mediterranean matorral of central Chile? Forest Ecology and Management 255, 1510–1515 (2008).

Gonzalez, S. & Ghermandi, L. Comparison of methods to estimate soil seed banks: the role of seed size and mass. Community Ecology 13, 238–242 (2012).

Gooden, B., French, K. & Turner, P. J. Invasion and management of a woody plant, Lantana camara L., alters vegetation diversity within wet sclerophyll forest in southeastern Australia. Forest Ecology and Management 257, 960–967 (2009).

Gooden, B., French, K., Turner, P. J. & Downey, P. O. Impact threshold for an alien plant invader, Lantana camara L., on native plant communities. Biological conservation 142, 2631–2641 (2009).

Gorchov, D. L. Pattern, adaptation, and constraint in fruiting synchrony within vertebrate-dispersed woody plants. Oikos 169–180 (1990).

Graham, E. H. Legumes for erosion control and wildlife. (US Department of Agriculture, 1941).

Greenway, P. J. & FGDF, V. Annotated check-list of plants occurring in Lake Manyara National Park. (1972).

Grieve, M. A modern herbal: the medicinal, culinary, cosmetic and economic properties, cultivation and folk-lore of herbs, grasses, fungi, shrubs, & trees with all their modern scientific uses. vol. 2 (Courier Corporation, 1971).
Grubb, P. J. Interpreting some outstanding features of the flora and vegetation of Madagascar. Perspectives in Plant Ecology, Evolution and Systematics 6, 125–146 (2003).
Grubb, P. J., Lee, W. G., Kollmann, J. & Wilson, J. B. Interaction of irradiance and soil nutrient supply on growth of seedlings of ten European tall-shrub species and Fagus sylvatica. Journal of Ecology 827–840 (1996).
Guevara, S. & Laborde, J. Monitoring seed dispersal at isolated standing trees in tropical pastures: consequences for local species availability. in Frugivory and seed dispersal: ecological and evolutionary aspects 319–338 (Springer, 1993).
Guillaumet, J.-L. The vegetation: an extraordinary diversity. Key Environments: Madagascar 27, 54 (1984).
Gupta, R. Flora of Lam Dal. Indian Forester 87, 316–324 (1961).
Gupta, R. Survey record of medicinal and aromatic plants of Chamba forest division, Himachal Pradesh. Indian Forester 90, 454–468 (1964).
Gurvich, D. E., Enrico, L. & Cingolani, A. M. Linking plant functional traits with post-fire sprouting vigour in woody species in central Argentina. Austral Ecology 30, 789–796 (2005).
Gutiérrez, J., Arancio, G. & Jaksic, F. Variation in vegetation and seed bank in a Chilean semi-arid community affected by ENSO 1997. Journal of Vegetation Science 11, 641–648 (2000).
Halvorson, W. L. & Patten, D. T. Productivity and flowering of winter ephemerals in relation to Sonoran Desert shrubs. American Midland Naturalist 311–319 (1975).
Hamann, O. Demographic studies of three indigenous stand-forming plant taxa (Scalesia, Opuntia, and Bursera) in the Galápagos Islands, Ecuador. Biodiversity and Conservation 10, 223–250 (2001).
Hampe, A. Large-scale geographical trends in fruit traits of vertebrate-dispersed temperate plants. Journal of Biogeography 30, 487–496 (2003).
Hanley, T. A. Potential management of young-growth stands for understory vegetation and wildlife habitat in southeastern Alaska. Landscape and Urban Planning 72, 95–112 (2005).
Harper, Jl. & White, J. The demography of plants. Annual review of ecology and systematics 5, 419–463 (1974).
Hartshorn, G. S. & Hammel, B. E. Vegetation types and floristic patterns. La Selva: ecology and natural history of a neotropical rain forest 73–89 (1994).
Hearn, D. J. Adenia (Passifloraceae) and its adaptive radiation: phylogeny and growth form diversification. Systematic Botany 31, 805–821 (2006).
Heenan, P. & Molloy, B. Taxonomy, ecology, and conservation of Olearia adenocarpa (Asteraceae), a new species from braided riverbeds in Canterbury, New Zealand. New Zealand Journal of Botany 42, 21–36 (2004).
Heide-Jørgensen, H. Parasitic flowering plants. (Brill, 2008).
Hemadri, K. New and interesting plant records. Indian Forester 94, 808–811 (1968).
Hemp, A. The banana forests of Kilimanjaro: biodiversity and conservation of the Chagga homegardens. Biodiversity & Conservation 15, 133–155 (Springer, 2006).
Henderson, L. Invasive, naturalized and casual alien plants in southern Africa: a summary based on the Southern African Plant Invaders Atlas (SAPIA). Bothalia 37, 215–248 (2007).
Hendricks, B. J. & Rieger, J. P. Description of nesting habitat for least Bell's vireo in San Diego County. (1989).

Hendricks, H., Novellie, P., Bond, W. & Midgley, J. Diet selection of goats in the communally grazed Richtersveld National Park. African Journal of Range and Forage Science 19, 1–11 (2002).
Hernandez-Stefanoni, J. L., Pineda, J. B. & Valdes-Valadez, G. Comparing the use of indigenous knowledge with classification and ordination techniques for assessing the species composition and structure of vegetation in a tropical forest. Environmental management 37, 686–702 (2006).
Herrel, A., Speck, T. & Rowe, N. P. Ecology and biomechanics: a mechanical approach to the ecology of animals and plants. (CRC Press, 2006).
Herrera, C. M. Breeding systems and dispersal-related maternal reproductive effort of Southern Spanish bird-dispersed plants. Evolution 36, 1299–1314 (1982).
Herrera, C. M. Vertebrate-dispersed plants of the Iberian peninsula: a study of fruit characteristics. Ecological monographs 57, 305–331 (1987).
Hochstedler, W. W., Slaughter, B. S., Gorchov, D. L., Saunders, L. P. & Stevens, M. H. H. Forest floor plant community response to experimental control of the invasive biennial, Alliaria petiolata (garlic mustard) 1. The Journal of the Torrey Botanical Society 134, 155–165 (2007).
Hoffmann, W. A. Post-burn reproduction of woody plants in a neotropical savanna: the relative importance of sexual and vegetative reproduction. Journal of Applied Ecology 35, 422–433 (1998).
Höft, R. & Höft, M. The differential effects of elephants on rain forest communities in the Shimba Hills, Kenya. Biological Conservation 73, 67–79 (1995).
Holm, A. M., Watson, I. W., Loneragan, W. A. & Adams, M. A. Loss of patch-scale heterogeneity on primary productivity and rainfall-use efficiency in Western Australia. Basic and Applied Ecology 4, 569–578 (2003).
Holmes, P. M. & Cowling, 6RM. Diversity, composition and guild structure relationships between soil-stored seed banks and mature vegetation in alien plant-invaded South African fynbos shrublands. Plant Ecology 133, 107–122 (1997).
Holmes, P., Esler, K., Richardson, D. & Witkowski, E. Guidelines for improved management of riparian zones invaded by alien plants in South Africa. South African Journal of Botany 74, 538–552 (2008).
Hong, L. et al. Ethnobotanical study on medicinal plants used by Maonan people in China. Journal of ethnobiology and ethnomedicine 11, 32 (2015).
Hou, H.-Y. & Xue-Yu, H. Vegetation of China with reference to its geographical distribution. Annals of the Missouri Botanical Garden 509–549 (1983).
Hovestadt, T., Yao, P. & Linsenmair, K. E. Seed dispersal mechanisms and the vegetation of forest islands in a West African forest-savanna mosaic (Comoé National Park, Ivory Coast). Plant ecology 144, 1–25 (1999).
Huang, J.-Y. et al. Changes in nitrogen resorption traits of six temperate grassland species along a multi-level N addition gradient. Plant and Soil 306, 149–158 (2008).
Hubbell, S. P., Howard, J. J. & Wiemer, D. F. Chemical leaf repellency to an attine ant: seasonal distribution among potential host plant species. Ecology 65, 1067–1076 (1984).
Hughes, L. et al. Predicting dispersal spectra: a minimal set of hypotheses based on plant attributes. Journal of Ecology 933–950 (1994).
Husain, W. Some New Records for Plants in the Upper Gangetic Plain. Indian Forester 93, 582–585 (1967).
Ibarra-Manríquez, G., Ricker, M., Angeles, G., Colín, S. S. & Colín, M. A. S. Useful plants of the Los Tuxtlas rain forest (Veracruz, Mexico): considerations of their market potential. Economic Botany 51, 362–376 (1997).
Irving, L. J. & Cameron, D. D. You are what you eat: interactions between root parasitic plants and their hosts. Advances in Botanical Research 50, 87–138 (2009).
Issar, R. & Uniyal, M. Orchids of Uttrakhand Himalayas. Indian Forester 93, 713–716 (1967).

Jain, S., Kotwal, N. N., & others. On the vegetation of Shahabad in Rajasthan. Indian Forester 86, 602–8 (1960).
Jaksic, F. M. Spatiotemporal variation patterns of plants and animals in San Carlos de Apoquindo, central Chile. Revista Chilena de Historia Natural 74, 477–502 (2001).
James, G. & Courtin, G. Stand structure and growth form of the birch transition community in an industrially damaged ecosystem, Sudbury, Ontario. Canadian Journal of Forest Research 15, 809–817 (1985).
Jamir, S. A. & Pandey, H. Vascular plant diversity in the sacred groves of Jaintia Hills in northeast India. Biodiversity and Conservation 12, 1497–1510 (2003).
Jiang, G., Tang, H., Yu, M., Dong, M. & Zhang, X. Response of photosynthesis of different plant functional types to environmental changes along Northeast China Transect. Trees-structure and Function 14, 72–82 (1999).
Jiang, M., Deng, H., Cai, Q. & Wu, G. Species richness in a riparian plant community along the banks of the Xiangxi River, the Three Gorges region. The International Journal of Sustainable Development & World Ecology 12, 60–67 (2005).
Jonsson, B. G. & Esseen, P.-A. Plant colonisation in small forest–floor patches: importance of plant group and disturbance traits. Ecography 21, 518–526 (1998).
Joubert, E. Habitat preference, distribution and status of the Hartmann Zebra Equus zebra hartmannae in South West Africa. Madoqua 1, 5–15 (1973).
Joubert, E. The physiographic, edaphic and vegetative characteristics found in the western Etosha National Park. Madoqua 1, 5–32 (1971).
Jung, H.-J. G., Batzli, G. O. & Seigler, D. S. Patterns in the phytochemistry of arctic plants. Biochemical Systematics and Ecology 7, 203–209 (1979).
Juvany, M. & Munné-Bosch, S. Sex-related differences in stress tolerance in dioecious plants: a critical appraisal in a physiological context. Journal of Experimental Botany 66, 6083–6092 (2015).
Kamatenesi-Mugisha, M. & Oryem-Origa, H. Medicinal plants used in some gynaecological morbidity ailments in western Uganda. African Journal of Ecology 45, 34–40 (2007).
Kamatenesi-Mugisha, M., Oryem-Origa, H., Odyek, O. & Makawiti, D. W. Medicinal plants used in the treatment of fungal and bacterial infections in and around Queen Elizabeth Biosphere Reserve, western Uganda. African Journal of Ecology 46, 90–97 (2008).
Kanodia, K. & Malick, K. New Plant Records and Critical Notes on some Plants from South Bihar. Indian Forester 92, 707–708 (1966).
Karnik, C. Some medicinal plants from Satpura mountains. Indian Forester 92, 173–183 (1966).
Kataki, S. & Panigrahi, G. Ranunculaceae in Assam and North East Frontier Agency. Indian Forester 90, 394–400 (1964).
Kazanis, D. & Arianoutsou, M. Long-term post-fire vegetation dynamics in Pinus halepensis forests of Central Greece: a functional group approach. Plant Ecology 171, 101–121 (2004).
Keating, P. L. Chronically disturbed páramo vegetation at a site in southern Ecuador. Journal of the Torrey Botanical Society 162–171 (2000).
Keeley, J. E. & Bond, W. J. Convergent seed germination in South African fynbos and Californian chaparral. Plant Ecology 133, 153–167 (1997).
Keeley, J. E. Reproductive cycles and fire regimes. in Proceedings of the Symposium on Fire Regimes and Ecosystem Properties, General Technical Report WO-26. USDA Forest Service, Washington, DC, USA 231–277 (1981).
Kelly, C. & Beerling, D. Plant life form, stomatal density and taxonomic relatedness: a reanalysis of Salisbury (1927). Functional Ecology 422–431 (1995).
Khan, K. U. et al. Investigation of traditional veterinary phytomedicines used in Deosai Plateau, Pakistan. Global Vet 15, 381–388 (2015).

Kharkwal, G. & Rawat, Y. S. Structure and composition of vegetation in subtropical forest of Kumaun Himalaya. African Journal of Plant Science 4, 116–121 (2010).

Kharkwal, G., Mehrotra, P., Rawat, Y. & Pangtey, Y. Phytodiversity and growth form in relation to altitudinal gradient in the Central Himalayan (Kumaun) region of India. CURRENT SCIENCE-BANGALORE- 89, 873 (2005).

Khuroo, A. A., Rashid, I., Reshi, Z., Dar, G. & Wafai, B. The alien flora of Kashmir Himalaya. Biological Invasions 9, 269–292 (2007).

Kidane, B., van Andel, T., van der Maesen, L. J. G. & Asfaw, Z. Use and management of traditional medicinal plants by Maale and Ari ethnic communities in southern Ethiopia. Journal of ethnobiology and ethnomedicine 10, 46 (2014).

Kikuzawa, K. Leaf survival of woody plants in deciduous broad-leaved forests. 2. Small trees and shrubs. Canadian Journal of Botany 62, 2551–2556 (1984).

Kinsman, S. Regeneration by fragmentation in tropical montane forest shrubs. American Journal of Botany 1626–1633 (1990).

Klinger, L. et al. Assessment of volatile organic compound emissions from ecosystems of China. Journal of Geophysical Research: Atmospheres 107, (2002).

Knapp, A. K. et al. Shrub encroachment in North American grasslands: shifts in growth form dominance rapidly alters control of ecosystem carbon inputs. Global Change Biology 14, 615–623 (2008).

Knapp, A. K., Hamerlynck, E. P., Ham, J. M. & Owensby, C. E. Responses in stomatal conductance to elevated CO2 in 12 grassland species that differ in growth form. Vegetatio 125, 31–41 (1996).

Kok, O., Fourie, L. & Barkhuizen, L. Diet of free ranging Angora goats in a False Upper Karoo veld type. South African Journal of Animal Science 24, 80–86 (1994).

Kostel-Hughes, F., Young, T. P. & McDonnell, M. J. The soil seed bank and its relationship to the aboveground vegetation in deciduous forests in New York City. Urban Ecosystems 2, 43–59 (1998).

Kpodar, M. S. et al. Ethnopharmacological survey of plants used in the treatment of diabetes mellitus in south of Togo (Maritime Region). Journal of Herbal Medicine 5, 147–152 (2015).

Kraaij, T. The flora of the Bontebok National Park in regional perspective. South African Journal of Botany 77, 455–473 (2011).

Kubitzki, K., Rohwer, J. & Bittrich, V. The families and genera of vascular plants. vol. 1 (Springer, 1990).

Kudo, G. & Ito, K. Plant distribution in relation to the length of the growing season in a snow-bed in the Taisetsu Mountains, northern Japan. Plant Ecology 98, 165–174 (1992).

Kummerow, J. Comparative phenology of Mediterranean-type plant communities. in Mediterranean-type ecosystems 300–317 (Springer, 1983).

Kuria, S., Wanyoike, M., Gachuiri, C. & Wahome, R. Nutritive value of important range forage species for camels in Marsabit district, Kenya. Tropical and Subtropical Agroecosystems 5, (2005).

La Roi, G. H., Strong, W. L. & Pluth, D. J. Understory plant community classifications as predictors of forest site quality for lodgepole pine and white spruce in west-central Alberta. Canadian Journal of Forest Research 18, 875–887 (1988).

Ladinig, U., Hacker, J., Neuner, G. & Wagner, J. How endangered is sexual reproduction of high-mountain plants by summer frosts? Frost resistance, frequency of frost events and risk assessment. Oecologia 171, 743–760 (2013).

Leimu, R. & Koricheva, J. A meta-analysis of genetic correlations between plant resistances to multiple enemies. The American Naturalist 168, E15–E37 (2006).

Leishman, M. R. & Thomson, V. P. Experimental evidence for the effects of additional water, nutrients and physical disturbance on invasive plants in low fertility Hawkesbury Sandstone soils, Sydney, Australia. Journal of Ecology 93, 38–49 (2005).

Leishman, M. R., Thomson, V. P. & Cooke, J. Native and exotic invasive plants have fundamentally similar carbon capture strategies. Journal of Ecology 98, 28–42 (2010).

Lemmens, R. & Lemmens, R. Plant resources of South-East Asia. (Pudoc, 1989).

Li, X., Liu, W. & Tang, C. Q. The role of the soil seed and seedling bank in the regeneration of diverse plant communities in the subtropical Ailao Mountains, Southwest China. Ecological research 25, 1171–1182 (2010).

Li, X., Zhang, Z. S., Zhang, J., Wang, X. & Jia, X. Association between vegetation patterns and soil properties in the southeastern Tengger Desert, China. Arid Land Research and Management 18, 369–383 (2004).

Li, Y., Ding, P., Huang, C. & Lu, S. Total tannin content of foods of François' Langur in Fusui, Guangxi, China: Preliminary study. Acta Ecologica Sinica 35, 16–22 (2015).

Liang, Y., Li, J., Li, J. & Valimaki, S. K. Impact of urbanization on plant diversity: a case study in built-up areas of Beijing. Forestry Studies in China 10, 179–188 (2008).

Litton, C. M. & Santelices, R. Early post-fire succession in a Nothofagus glauca forest in the Coastal Cordillera of south-central Chile. International Journal of Wildland Fire 11, 115–125 (2002).

Liu, C., Liu, Y., Guo, K., Wang, S. & Yang, Y. Concentrations and resorption patterns of 13 nutrients in different plant functional types in the karst region of south-western China. Annals of Botany 113, 873–885 (2014).

Liu, C.-C., Liu, Y.-G., Fan, D.-Y. & Guo, K. Plant drought tolerance assessment for re-vegetation in heterogeneous karst landscapes of southwestern China. Flora-Morphology, Distribution, Functional Ecology of Plants 207, 30–38 (2012).

López, R. P. & Valdivia, S. The importance of shrub cover for four cactus species differing in growth form in an Andean semi-desert. Journal of Vegetation Science 18, 263–270 (2007).

Lötter, M., Mucina, L. & Witkowski, E. Classification of the indigenous forests of Mpumalanga Province, South Africa. South African Journal of Botany 90, 37–51 (2014).

Louhaichi, M., Salkini, A., Estita, H. & Belkhir, S. Initial assessment of medicinal plants across the Libyan Mediterranean coast. Adv Environ Biol 5, 359–370 (2011).

Lucrezi, S., Saayman, M. & van der Merwe, P. Influence of infrastructure development on the vegetation community structure of coastal dunes: Jeffreys Bay, South Africa. Journal of coastal conservation 18, 193–211 (2014).

Lulekal, E., Asfaw, Z., Kelbessa, E. & Van Damme, P. Ethnoveterinary plants of Ankober District, North Shewa Zone, Amhara Region, Ethiopia. Journal of ethnobiology and ethnomedicine 10, 21 (2014).

Machado, I. C., Lopes, A. V. & Sazima, M. Plant sexual systems and a review of the breeding system studies in the Caatinga, a Brazilian tropical dry forest. Annals of Botany 97, 277–287 (2006).

MacLean Jr, S. & Jensen, T. Food plant selection by insect herbivores in Alaskan arctic tundra: the role of plant life form. Oikos 211–221 (1985).

Maheshwari, J. Notes on the flora of black cotton soils in East Nimar, Madhya Pradesh. Indian Forester 88, 115–135 (1962).

Mandle, L. Balancing biodiversity and human land use: effects of fire, grazing and harvest on plant individuals, populations and communities in the Western Ghats, India. (University of Hawai'i at Manoa, 2012).

Manea, A. & Leishman, M. R. Competitive interactions between native and invasive exotic plant species are altered under elevated carbon dioxide. Oecologia 165, 735–744 (2011).

Mark, A., Scott, G., Sanderson, F. & James, P. Forest succession on landslides above Lake Thomson, Fiordland. New Zealand Journal of Botany 2, 60–89 (1964).

Maroyi, A. & Mosina, G. K. Medicinal plants and traditional practices in peri-urban domestic gardens of the Limpopo province, South Africa. (2014).

Maroyi, A. Alternative Medicines for HIV/AIDS in Resource-Poor Settings: Insight from Traditional Medicines Use in Sub-Saharan Africa. Tropical Journal of Pharmaceutical Research 13, 1527–1536 (2014).

Maroyi, A. Traditional use of medicinal plants in south-central Zimbabwe: review and perspectives. Journal of Ethnobiology and Ethnomedicine 9, 31 (2013).

Marshall, J., Ehleringer, J. R., Schulze, E.-D. & Farquhar, G. Carbon isotope composition, gas exchange and heterotrophy in Australian mistletoes. Functional Ecology 237–241 (1994).

Martin, P. H., Sherman, R. E. & Fahey, T. J. Forty years of tropical forest recovery from agriculture: structure and floristics of secondary and old-growth riparian forests in the Dominican Republic. Biotropica 36, 297–317 (2004).

Martinez, M. L., Moreno-Casasola, P. & Vazquez, G. Effects of disturbance by sand movement and inundation by water on tropical dune vegetation dynamics. Canadian Journal of Botany 75, 2005–2014 (1997).

Mason, T. J., French, K. & Russell, K. Are competitive effects of native species on an invader mediated by water availability? Journal of Vegetation Science 23, 657–666 (2012).

Mason, T., French, K. & Russell, K. Moderate impacts of plant invasion and management regimes in coastal hind dune seed banks. Biological Conservation 134, 428–439 (2007).

Matesanz, S. & Valladares, F. Ecological and evolutionary responses of Mediterranean plants to global change. Environmental and Experimental Botany 103, 53–67 (2014).

Mathibela, K. M. An investigation into aspects of medicinal plant use by traditional healers from Blouberg Mountain, Limpopo Province, South Africa. (University of Limpopo (Turfloop Campus), 2013).

Mathiesen, S., Haga, Ø., Kaino, T. & Tyler, N. Diet composition, rumen papillation and maintenance of carcass mass in female Norwegian reindeer (Rangifer tarandus tarandus) in winter. Journal of Zoology 251, 129–138 (2000).

Mathur, C. & Verma, S. Vegetation types of Sirohi forest division. Indian Forester 90, 413–430 (1964).

Matimele, H. A. An assessment of the distribution and conservation status of endemic and near endemic plant species in Maputaland. (University of Cape Town, 2016).

Matlack, G. R. Plant Species Migration in a Mixed-History Forest Landscape in Eastern North America. Ecology 75, 1491–1502 (1994).

Maza-Villalobos, S., Lemus-Herrera, C. & Martínez-Ramos, M. Successional trends in soil seed banks of abandoned pastures of a Neotropical dry region. Journal of Tropical Ecology 27, 35–49 (2011).

McArthur, E. D. Breeding systems in shrubs. Biology and Utilization of Shrubs 341–361 (1989).

McKell, C. The biology and utilization of shrubs. (Elsevier, 2012).

Medrano, H., Flexas, J. & Galmés, J. Variability in water use efficiency at the leaf level among Mediterranean plants with different growth forms. Plant and Soil 317, 17–29 (2009).

Megersa, M., Beyene, A., Ambelu, A. & Woldeab, B. The use of indigenous plant species for drinking water treatment in developing countries: a review. J Bio Env Sci 5, 269–281 (2014).

Mehra, B. & Gokulpure, R. Recorded and Unrecorded Lac-hosts from Madhya Pradesh. Indian Forester 93, 694–707 (1967).

Meir, P., Levy, P. E., Grace, J. & Jarvis, P. G. Photosynthetic parameters from two contrasting woody vegetation types in West Africa. Plant Ecology 192, 277–287 (2007).

Michelsen, A., Quarmby, C., Sleep, D. & Jonasson, S. Vascular plant 15 N natural abundance in heath and forest tundra ecosystems is closely correlated with presence and type of mycorrhizal fungi in roots. Oecologia 115, 406–418 (1998).
Mills, K. The conservation reserve system in the South Coast Region of NSW looks impressive, but does it adequately conserve threatened plant species? Cunninghamia 12, 325–337 (2012).
Mishra, B., Tripathi, O., Tripathi, R. & Pandey, H. Effects of anthropogenic disturbance on plant diversity and community structure of a sacred grove in Meghalaya, northeast India. Biodiversity and Conservation 13, 421–436 (2004).
Mohandass, D. & Davidar, P. Floristic structure and diversity of a tropical montane evergreen forest (shola) of the Nilgiri Mountains, southern India. Tropical Ecology 50, 219 (2009).
Mooney, H. Carbon-gaining capacity and allocation patterns of mediterranean-climate plants. in Mediterranean-Type Ecosystems 103–119 (Springer, 1983).
MOORE, J. Floral Lists from Five Study Sites of Apes in the African Tropical Forests. Plants of the Tongwe East Forest Reserve (Ugalla), Tanzania. Tropics 3, 333–340 (1994).
Mort, M. E., Levsen, N., Randle, C. P., Van Jaarsveld, E. & Palmer, A. Phylogenetics and diversification of Cotyledon (Crassulaceae) inferred from nuclear and chloroplast DNA sequence data. American Journal of Botany 92, 1170–1176 (2005).
Morton, J. F. & others. Pestiferous spread of many ornamental and fruit species in south Florida. in Proc. Fla. State Hort. Soc vol. 89 348–353 (1976).
Moshi, M. J., Otieno, D. F., Mbabazi, P. K. & Weisheit, A. The Ethnomedicine of the Haya people of Bugabo ward, Kagera Region, north western Tanzania. Journal of ethnobiology and ethnomedicine 5, 24 (2009).
Moxham, C. & Turner, V. The effect of fragmentation on the threatened plant community Coastal Moonah Woodland in Victoria, Australia. Urban Ecosystems 14, 569–583 (2011).
Mucina, L. et al. Succulent karoo biome. the vegetation of south Africa, lesotho and swaziland. strelitzia 19, 220–299 (2006).
MUKINYA, J. G. Feeding and drinking habits of the black rhinoceros in Masai Mara Game Reserve. African Journal of Ecology 15, 125–138 (1977).
Mullah, C. J., Totland, Ø. & Klanderud, K. Recovery of plant species richness and composition in an abandoned forest settlement area in Kenya. Restoration Ecology 20, 462–474 (2012).
Murray, B. R. & Phillips, M. L. Investment in seed dispersal structures is linked to invasiveness in exotic plant species of south-eastern Australia. Biological Invasions 12, 2265–2275 (2010).
Murray-Hudson, M., Combs, F., Wolski, P. & Brown, M. A vegetation-based hierarchical classification for seasonally pulsed floodplains in the Okavango Delta, Botswana. African Journal of Aquatic Science 36, 223–234 (2011).
Murthy, A. K. Economics on Railway Sleeper Supplies in Karimnager (East) DIVN. Andhra Pradesh. Indian Forester 93, 455–468 (1967).
MUSIL, C. F., CHIMPHANGO, S. B. & DAKORA, F. D. Effects of Elevated Ultraviolet-B Radiation on Native and Cultivated Plants of Southern Africa. Annals of Botany 90, 127–137 (2002).
Mussarat, S. et al. Use of ethnomedicinal plants by the people living around Indus River. Evidence-Based Complementary and Alternative Medicine 2014, (2014).
Musselman, L. J. The biology of Striga, Orobanche, and other root-parasitic weeds. Annual review of phytopathology 18, 463–489 (1980).
Muthaura, C., Rukunga, G., Chhabra, S., Mungai, G. & Njagi, E. Traditional antimalarial phytotherapy remedies used by the Kwale community of the Kenyan Coast. Journal of Ethnopharmacology 114, 377–386 (2007).

Muthaura, C., Rukunga, G., Chhabra, S., Mungai, G. & Njagi, E. Traditional phytotherapy of some remedies used in treatment of malaria in Meru district of Kenya. South African Journal of Botany 73, 402–411 (2007).
Muthee, J. et al. Ethnobotanical study of anthelmintic and other medicinal plants traditionally used in Loitoktok district of Kenya. Journal of Ethnopharmacology 135, 15–21 (2011).
Muthukumar, T., Senthilkumar, M., Rajangam, M. & Udaiyan, K. Arbuscular mycorrhizal morphology and dark septate fungal associations in medicinal and aromatic plants of Western Ghats, Southern India. Mycorrhiza 17, 11–24 (2006).
Nadembega, P., Boussim, J. I., Nikiema, J. B., Poli, F. & Antognoni, F. Medicinal plants in baskoure, kourittenga province, Burkina Faso: an ethnobotanical study. Journal of Ethnopharmacology 133, 378–395 (2011).
Naidu, K. & Rao, R. A contribution to the flora of Tirupati Hills. Indian Forester 93, 123–135 (1967).
Nampanzira, D. K., Kabasa, J. D., Nalule, S. A., Nakalembe, I. & Tabuti, J. R. S. Characterization of the goat feeding system among rural small holder farmers in the semi-arid regions of Uganda. SpringerPlus 4, 188 (2015).
Namsa, N. D., Mandal, M., Tangjang, S. & Mandal, S. C. Ethnobotany of the Monpa ethnic group at Arunachal Pradesh, India. Journal of Ethnobiology and Ethnomedicine 7, 31 (2011).
Nanyingi, M. O. et al. Ethnopharmacological survey of Samburu district, Kenya. Journal of Ethnobiology and Ethnomedicine 4, 14 (2008).
Navarro, T., Alados, C. L. & Cabezudo, B. Changes in plant functional types in response to goat and sheep grazing in two semi-arid shrublands of SE Spain. Journal of Arid Environments 64, 298–322 (2006).
Navarro, T., El Oualidi, J., Taleb, M. S., Pascual, V. & Cabezudo, B. Dispersal traits and dispersal patterns in an oro-Mediterranean thorn cushion plant formation of the eastern High Atlas, Morocco. Flora-Morphology, Distribution, Functional Ecology of Plants 204, 658–672 (2009).
Navas, M.-L., Roumet, C., Bellmann, A., Laurent, G. & Garnier, E. Suites of plant traits in species from different stages of a Mediterranean secondary succession. Plant Biology 12, 183–196 (2010).
Naveh, Z. Degradation and rehabilitation of Mediterranean land-scapes: Neotechnological degradation of Mediterranean landscapes and their restoration with drought resistant plants. Landscape Planning 2, 133–146 (1975).
Ndawula, J., Tweheyo, M., Tumusiime, D. M. & Eilu, G. Understanding sitatunga (Tragelaphus spekii) habitats through diet analysis in Rushebeya-Kanyabaha wetland, Uganda. African Journal of Ecology 49, 481–489 (2011).
Negi, G., Rikhari, H. & Singh, S. Phenological features in relation to growth forms and biomass accumulation in an alpine meadow of the Central Himalaya. Plant Ecology 101, 161–170 (1992).
Ng, S. & Corlett, R. The bad biodiversity: alien plant species in Hong Kong. (2002).
Nguta, J., Mbaria, J., Gakuya, D., Gathumbi, P. & Kiama, S. Antimalarial herbal remedies of Msambweni, Kenya. Journal of Ethnopharmacology 128, 424–432 (2010).
Nickrent, D. L. & Musselman, L. J. Introduction to parasitic flowering plants. The Plant health instructor 13, 300–315 (2004).
Nickrent, D. L. Phylogenetic origins of parasitic plants. Parasitic plants of the Iberian Peninsula and Balearic Islands 29–56 (2002).
Nilsson, C., Ekblad, A., Gardfjell, M. & Carlberg, B. Long-term effects of river regulation on river margin vegetation. Journal of Applied Ecology 963–987 (1991).
Norton, D. A. & Reid, N. Lessons in ecosystem management from management of threatened and pest loranthaceous mistletoes in New Zealand and Australia. Conservation biology 11, 759–769 (1997).

Odhiambo, J. A., Lukhoba, C. W. & Dossaji, S. F. Evaluation of herbs as potential drugs/medicines. African Journal of Traditional, Complementary and Alternative Medicines 8, (2011).
Oechel, W. C., Lawrence, W., Mustafa, J. & Martínez, J. Energy and carbon acquisition. in Resource use by chaparral and matorral 151–183 (Springer, 1981).
Ogbuewu, I. et al. Studies on the diversity of medicinal plant species utilized for goat reproduction in Abia State Nigeria. Journal of Livestock Science (ISSN online 2277-6214) 7, 1–12 (2016).
Oloo, T. W., Brett, R. & Young, T. P. Seasonal variation in the feeding ecology of black rhinoceros (Diceros bicornis L.) in Laikipia, Kenya. African Journal of Ecology 32, 142–157 (1994).
Onaindia, M., Dominguez, I., Albizu, I., Garbisu, C. & Amezaga, I. Vegetation diversity and vertical structure as indicators of forest disturbance. Forest Ecology and Management 195, 341–354 (2004).
Orsham, G. Plant pheno-morphological studies in Mediterranean type ecosystems. vol. 12 (Springer Science & Business Media, 2012).
Orshan, G. Approaches to the definition of Mediterranean growth forms. in Mediterranean-type Ecosystems 86–100 (Springer, 1983).
OTANG, W. M., GRIERSON, D. S., Ndip, N., & others. Ethnobotanical survey of medicinal plants used in the management of opportunistic fungal infections in HIV/AIDS patients in the Amathole District of the Eastern Cape Province, South Africa. Journal of Medicinal Plants Research 6, 2071–2080 (2012).
Otani, T. Seed dispersal and predation of fleshy-fruited plants by Japanese macaques in the cool temperate zone of northern Japan. Mammal study 28, 153–156 (2003).
Padayachee, B. & Baijnath, H. An overview of the medicinal importance of Moringaceae. Journal of Medicinal Plants Research 6, 5831–5839 (2012).
Parker, J. L. & Parsons, B. New plant records from the Big Island for 2010-2011. Bishop Museum Occasional Papers 113, 65–74 (2012).
Pate, J., Stewart, G. & Unkovich, M. 15N natural abundance of plant and soil components of a Banksia woodland ecosystem in relation to nitrate utilization, life form, mycorrhizal status and N2-fixing abilities of component species. Plant, Cell & Environment 16, 365–373 (1993).
Patel, R. G., Patel, Y. B., Mankad, A. & Jasrai, Y. Climbers in urban setup-Ahmedabad and Gandhinagar. Lifesci Leafl 2, 1–8 (2013).
Pei, S., Fu, H. & Wan, C. Changes in soil properties and vegetation following exclosure and grazing in degraded Alxa desert steppe of Inner Mongolia, China. Agriculture, Ecosystems & Environment 124, 33–39 (2008).
Pickard, J. Rare or threatened vascular plants of Lord Howe Island. Biological conservation 27, 125–139 (1983).
Pickett, C. H. Enhancing biological control: habitat management to promote natural enemies of agricultural pests. (Univ of California Press, 1998).
Pignone, D. & Hammer, K. Parasitic angiosperms as cultivated plants? Genetic resources and crop evolution 63, 1273–1284 (2016).
Pitt-Schenkel, C. Some important communities of warm temperate rain forest at Magamba, West Usambara, Tanganyika Territory. The Journal of Ecology 50–81 (1938).
Platts, P. J. et al. Can distribution models help refine inventory-based estimates of conservation priority? A case study in the Eastern Arc forests of Tanzania and Kenya. Diversity and Distributions 16, 628–642 (2010).
Pohl, M., Stroude, R., Buttler, A. & Rixen, C. Functional traits and root morphology of alpine plants. Annals of Botany mcr169 (2011).
Porembski, S. West African inselberg vegetation. in Inselbergs 177–211 (Springer, 2000).

Prasad, U. A Phylogenetic-cum-artificial Analysis of the Host-Plants of the Lac Insect, Laccifer lacca (Kerr), with a View to Determine Host-preferences. Indian Forester 91, 297–308 (1965).

Press, M. & Graves, J. Parasitic plants. (Springer Science & Business Media, 1995).

Press, M. C. & Phoenix, G. K. Impacts of parasitic plants on natural communities. New phytologist 166, 737–751 (2005).

Press, M., Potter, J., Burke, M., Callaghan, T. & Lee, J. Responses of a subarctic dwarf shrub heath community to simulated environmental change. Journal of Ecology 86, 315–327 (1998).

Puri, G. & Arora, R. Some medicinal ferns from Western India. Indian Forester 87, 179–183 (1961).

Pyke, D. A. Invasive exotic plants in sagebrush ecosystems of the intermountain west. in Proceedings: sagebrush steppe ecosystems symposium: 2000 43–54 (1999).

Quiroz, D. et al. Quantifying the domestic market in herbal medicine in Benin, West Africa. Journal of Ethnopharmacology 151, 1100–1108 (2014).

Rahlao, S., Hoffman, M., Todd, S. & McGrath, K. Long-term vegetation change in the Succulent Karoo, South Africa following 67 years of rest from grazing. Journal of Arid Environments 72, 808–819 (2008).

Rahman, M. & others. A study on exploration of ethnobotanical knowledge of rural community in Bangladesh: basis for biodiversity conservation. ISRN Biodiversity 2013, (2013).

Raizada, M. & Saxena, H. New Plant Records for North and North-West India. Indian Forester 88, 702–704 (1962).

Raizada, M. & Sharma, V. New Plant Records for the Upper Gangetic Plain from Ajmer-Merwara. Indian Forester 88, 356–369 (1962).

Raizada, M. Mussoorie and its Plants: a Preliminary Survey. Indian Forester 85, 668–690 (1959).

Raizada, M., Hingorani, G. & Gales, U. M. Plants of Chin Hills (Burma). Indian Forester 88, 498–509 (1962).

Ram, J., Singh, S. & Singh, J. Community level phenology of grassland above treeline in central Himalaya, India. Arctic and Alpine Research 325–332 (1988).

Ramam, S. & others. Root development in alluvial grasslands of Varanasi. Indian Forester 96, 100–110 (1970).

Ramsay, P. & Oxley, E. The growth form composition of plant communities in the Ecuadorian páramos. Plant Ecology 131, 173–192 (1997).

Ramsay, P. M. Diurnal temperature variation in the major growth forms of an Ecuadorian páramo plant community. The Ecology of Volcan Chiles: High-Altitude Ecosystems of the Ecuador-Colombia Border. Plymouth: Pebble and Shells 101–112 (2001).

Rana, J., Singh, A., Sharma, Y., Pradheep, K. & Mendiratta, N. Dynamics of plant bioresources in Western Himalayan region of India- watershed based study. Current Science(Bangalore) 98, 192–203 (2010).

Rao, C. Plant Collection in Eastern Nepal. Indian Forester 93, 41–60 (1967).

Rao, G. S. & Kumari, G. New records of plants from South India. Nelumbo 9, 185–189 (1967).

Rao, R. S., Wadhwa, B. & Ansari, M. Comparative Studies on the Distribution of some Useful Trees of Tropical Evergreen forests in the Western and the Eastern Parts of India. Indian Forester 87, 220–241 (1961).

Regassa, R. Manuscript Info Abstract. International Journal 1, 308–328 (2013).

Reid, N. & Shamoun, S. F. Contrasting research approaches to managing mistletoes in commercial forests and wooded pastures This minireview is one of a collection of papers based on a presentation from the Stem and Shoot Fungal Pathogens and Parasitic Plants: the Values of Biological Diversity session of the XXII International

Union of Forestry Research Organization World Congress meeting held in Brisbane, Queensland, Australia, in 2005. Botany 87, 1–9 (2008).

Rejmánek, M. Species richness and resistance to invasions. in Biodiversity and ecosystem processes in tropical forests 153–172 (Springer, 1996).

Ren, H., Yang, L. & Liu, N. Nurse plant theory and its application in ecological restoration in lower subtropics of China. Progress in Natural Science 18, 137–142 (2008).

Revell, D. et al. Australian perennial shrub species add value to the feed base of grazing livestock in low-to medium-rainfall zones. Animal Production Science 53, 1221–1230 (2013).

Rice, P. M., Toney, C. & Sacco, R. Potential exotic plant species invading the Blackfoot Drainage. (1997).

Richards, P. Ecological notes on West African vegetation III. The upland forests of Cameroons Mountain. The Journal of Ecology 529–554 (1963).

Robert, E. M. et al. Successive cambia: a developmental oddity or an adaptive structure? PloS one 6, e16558 (2011).

Roberts, M. R. & Zhu, L. Early response of the herbaceous layer to harvesting in a mixed coniferous–deciduous forest in New Brunswick, Canada. Forest Ecology and Management 155, 17–31 (2002).

Rossatto, D. R., Kolb, R. M. & Franco, A. C. Leaf anatomy is associated with the type of growth form in Neotropical savanna plants. Botany 93, 507–518 (2015).

Roux, C. Feeding ecology, space use and habitat selection of elephants in two enclosed game reserves in the Eastern Cape Province, South Africa. (Rhodes University, 2006).

Rowe, N. & Speck, T. Plant growth forms: an ecological and evolutionary perspective. New phytologist 166, 61–72 (2005).

Rowe, N. P. & Speck, T. Biomechanics of plant growth forms: the trouble with fossil plants. Review of Palaeobotany and Palynology 102, 43–62 (1998).

Rowe, N. P., Isnard, S., Gallenmüller, F. & Speck, T. Diversity of mechanical architectures in climbing plants: an ecological perspective. Ecology and biomechanics: a mechanical approach to the ecology of animals and plants. Boca Raton, Florida, USA: Taylor & Francis 35–59 (2006).

Royer, D. L. & Wilf, P. Why do toothed leaves correlate with cold climates? Gas exchange at leaf margins provides new insights into a classic paleotemperature proxy. International Journal of Plant Sciences 167, 11–18 (2006).

Ruan, C.-J., Chen, S.-C., Li, Q. & da Silva, J. A. T. Adaptive evolution of context-dependent style curvature in some species of the Malvaceae: a molecular phylogenetic approach. Plant systematics and evolution 297, 57 (2011).

Rubiales, D. et al. Screening techniques and sources of resistance against parasitic weeds in grain legumes. Euphytica 147, 187–199 (2006).

Rundel, P. W. & Mahu, M. Community structure and diversity in a coastal fog desert in northern Chile. Flora 165, 493–505 (1976).

Ruwanza, S., Gaertner, M., Esler, K. & Richardson, D. The effectiveness of active and passive restoration on recovery of indigenous vegetation in riparian zones in the Western Cape, South Africa: A preliminary assessment. South African Journal of Botany 88, 132–141 (2013).

Ruwanza, S., Gaertner, M., Esler, K. J. & Richardson, D. M. Both complete clearing and thinning of invasive trees lead to short-term recovery of native riparian vegetation in the Western Cape, South Africa. Applied Vegetation Science 16, 193–204 (2013).

Sahai, R. & Sinha, A. A supplement to the aquatic and swampy vegetation of Gorakhpur. Indian Forester 94, 819–821 (1968).

Sahni, K. A contribution to the Flora of Kameng and Subansiri Districts, NEFA. Indian Forester 95, 330–352 (1969).

Sahni, K. Forest tree introduction in India, its scope and importance. Indian Forester 91, 43–57 (1965).

Sakai, A., Sato, S., Sakai, T., Kuramoto, S. & Tabuchi, R. A soil seed bank in a mature conifer plantation and establishment of seedlings after clear-cutting in southwest Japan. Journal of Forest Research 10, 295–304 (2005).

Sanaiotti, T. M. & Magnusson, W. E. Effects of annual fires on the production of fleshy fruits eaten by birds in a Brazilian Amazonian savanna. Journal of Tropical Ecology 11, 53–65 (1995).

Sanchez, P. A. Pasture production in acid soils of the tropics. vol. 5 (CIAT, 1979).

Santapau, H. Preservation of the Natural Flora in the National Parks of India. Indian Forester 95, 711–714 (1969).

Santiago, L. & Wright, S. Leaf functional traits of tropical forest plants in relation to growth form. Functional Ecology 21, 19–27 (2007).

Sarin, Y. Medicinal, Quasi-Medicinal and Economic Plants of Bhadarwah Forest Division. Indian Forester 91, 559–572 (1965).

Sarthou, C. & Villiers, J.-F. Epilithic plant communities on inselbergs in French Guiana. Journal of Vegetation Science 9, 847–860 (1998).

Schenk, H. J. & Jackson, R. B. Rooting depths, lateral root spreads and below-ground/above-ground allometries of plants in water-limited ecosystems. Journal of Ecology 90, 480–494 (2002).

Schenk, H. J. Clonal splitting in desert shrubs. Plant Ecology 141, 41–52 (1999).

Schmidt, E., Lotter, M. & McCleland, W. Trees and shrubs of Mpumalanga and Kruger national park. (Jacana Media, 2002).

Schmiedel, U. The quartz fields of Southern Africa-flora, phytogeography, vegetation, and habitat ecology. (Universität zu Köln, 2002).

Schmitt, C. B., Senbeta, F., Denich, M., Preisinger, H. & Boehmer, H. J. Wild coffee management and plant diversity in the montane rainforest of southwestern Ethiopia. African Journal of Ecology 48, 78–86 (2010).

Schoonmaker, P. & McKee, A. Species composition and diversity during secondary succession of coniferous forests in the western Cascade Mountains of Oregon. Forest Science 34, 960–979 (1988).

Schradin, C. & Pillay, N. Female striped mice (Rhabdomys pumilio) change their home ranges in response to seasonal variation in food availability. Behavioral Ecology 17, 452–458 (2006).

Sebastine, K. & Balakrishnan, N. A contribution to the flora of North Eastern Madhya Pradesh. Indian Forester 89, 487–491 (1963).

Senbeta, F. & Denich, M. Effects of wild coffee management on species diversity in the Afromontane rainforests of Ethiopia. Forest Ecology and Management 232, 68–74 (2006).

Seth, S., Desarker, B., & others. Poplar cultivation. Indian Forester 86, 21–7 (1960).

Shah, G. Further Cotributions to the Vegetation of Baroda. Indian Forester 89, 286–290 (1963).

Shaheen, H., Qureshi, R., Iqbal, S. & Qasem, M. F. Seasonal availability and palatability of native flora of Santh Saroola Kotli Sattian, Rawalpindi, Pakistan. African Journal of Plant Science 8, 92–102 (2014).

Shankarnarayan, K. & Gupta, R. The Vegetation of Coimbatore District. Indian Forester 85, 533–541 (1959).

Sharma, B. Root systems of some desert plants at Churu, Rajasthan. Indian Forester 94, 240–246 (1968).

Sharma, P., Rana, J., Devi, U., Randhawa, S. & Kumar, R. Floristic diversity and distribution pattern of plant communities along altitudinal gradient in Sangla Valley, Northwest Himalaya. The Scientific World Journal 2014, (2014).

Shi, J. & Zhu, H. Tree species composition and diversity of tropical mountain cloud forest in the Yunnan, southwestern China. Ecological Research 24, 83–92 (2009).

Shibayama, T. et al. Effects of fire on the recruitment of rain forest vegetation beneath Pinus caribaea plantations, Sri Lanka. Forest ecology and management 226, 357–363 (2006).
Shmida, A. & Whittaker, R. Pattern and biological microsite effects in two shrub communities, southern California. Ecology 62, 234–251 (1981).
Siccama, T., Bormann, F. & Likens, G. The Hubbard Brook ecosystem study: productivity, nutrients, and phytosociology of the herbaceous layer. Ecological Monographs 40, 389–402 (1970).
Sidhu, S. Chromosomal studies of some mangrove species. Indian Forester 88, 585–592 (1962).
Sidhu, S. Studies on the mangroves of India 1. East Godavari region. Indian Forester 89, 337–351 (1963).
Siebert, F. & Siebert, S. Dolomitic vegetation of the Sterkfontein Caves World Heritage Site and its importance in the conservation of Rocky Highveld Grassland. Koedoe 48, 17–31 (2005).
Sierra-Almeida, A., Cavieres, L. A. & Bravo, L. A. Freezing resistance of high-elevation plant species is not related to their height or growth-form in the Central Chilean Andes. Environmental and Experimental Botany 69, 273–278 (2010).
Sierra-Almeida, A., Cavieres, L. A. & Bravo, L. A. Freezing resistance varies within the growing season and with elevation in high-Andean species of central Chile. New Phytologist 182, 461–469 (2009).
Simon, M. F. et al. Recent assembly of the Cerrado, a neotropical plant diversity hotspot, by in situ evolution of adaptations to fire. Proc Natl Acad Sci USA 106, 20359–20364 (2009).
Singh, H., Agnihotri, P., Pande, P. & Husain, T. Biodiversity conservation through a traditional beliefs system in Indian Himalaya: a case study from Nakuleshwar sacred grove. The Environmentalist 31, 246 (2011).
Singhakumara, B. M. P., Uduporuwa, R. S. P. & Ashton, P. M. S. Soil Seed Banks in Relation to Light and Topographic Position of a Hill Dipterocarp Forest in Sri Lanka 1. Biotropica 32, 190–196 (2000).
Smith, C. W. Impact of alien plants on Hawaii's native biota. Hawaii's terrestrial ecosystems: preservation and management 180–250 (1985).
Songachan, L., Kayang, H. & Lyngdoh, I. Colonization of arbuscular mycorrhizal fungi in moderately degraded sub-tropical forest stands of Meghalaya, Northeast India. (2014).
Speed, J. D., Cooper, E. J., Jónsdóttir, I. S., Van Der Wal, R. & Woodin, S. J. Plant community properties predict vegetation resilience to herbivore disturbance in the Arctic. Journal of Ecology 98, 1002–1013 (2010).
Spencer, R. Horticultural flora of south-eastern Australia. vol. 3 (UNSW press, 1995).
Staver, C. The role of weeds in the productivity of Amazonian bush fallow agriculture. Experimental Agriculture 27, 287–304 (1991).
Stewart, G. R. & Press, M. C. The physiology and biochemistry of parasitic angiosperms. Annual review of plant biology 41, 127–151 (1990).
Stylinski, C. D. & Allen, E. B. Lack of native species recovery following severe exotic disturbance in southern Californian shrublands. Journal of Applied Ecology 36, 544–554 (1999).
Subramanian, K. Further Contribution to the Flora of Boluvampatti Valley Forests, Coimbatore District, Madras State. Indian Forester 92, 39–50 (1966).
Swanborough, P. & Westoby, M. Seedling relative growth rate and its components in relation to seed size: phylogenetically independent contrasts. Functional Ecology 176–184 (1996).
Swearingen, J., Reshetiloff, K., Slattery, B. & Zwicker, S. Plant invaders of mid-Atlantic natural areas. National Parks Services and US Fish and Wildlife Service. Washington, DC. 168pp (2010).

Tabuti, J., Dhillion, S. & Lye, K. The status of wild food plants in Bulamogi County, Uganda. International Journal of Food Sciences and Nutrition 55, 485–498 (2004).
Tariq, A. et al. Ethnomedicinal evaluation of medicinal plants used against gastrointestinal complaints. BioMed research international 2015, (2015).
Tariq, A., Mussarat, S. & Adnan, M. Review on ethnomedicinal, phytochemical and pharmacological evidence of Himalayan anticancer plants. Journal of Ethnopharmacology 164, 96–119 (2015).
Taschler, D. & Neuner, G. Summer frost resistance and freezing patterns measured in situ in leaves of major alpine plant growth forms in relation to their upper distribution boundary. Plant, Cell & Environment 27, 737–746 (2004).
Tchouto, M. G. P. Plant diversity in a Central African rain forest, implications for biodiversity conservation in Cameroon. (2004).
Tekle, Y. An ethno-veterinary botanical survey of medicinal plants in Kochore district of Gedeo Zone, Southern Nations Nationalities and Peoples Regional State (SNNPRs). Ethiopia. J. Sci. Innov. Res 3, 433–445 (2014).
Teklehaymanot, T. & Giday, M. Quantitative ethnobotany of medicinal plants used by Kara and Kwego semi-pastoralist people in lower Omo River Valley, Debub Omo Zone, Southern Nations, Nationalities and Peoples Regional State, Ethiopia. Journal of Ethnopharmacology 130, 76–84 (2010).
Tennakoon, K., Pate, J. & Arthur, D. Ecophysiological Aspects of the Woody Root HemiparasiteSantalum acuminatum (R. Br.) A. DC and its Common Hosts in South Western Australia. Annals of Botany 80, 245–256 (1997).
Thilenius, J. F. The Quercus garryana forests of the Willamette valley, Oregon. Ecology 49, 1124–1133 (1968).
Thomas, E. & Van Damme, P. Plant use and management in homegardens and swiddens: evidence from the Bolivian Amazon. Agroforestry Systems 80, 131–152 (2010).
Thomas, E., Vandebroek, I. & Van Damme, P. Valuation of forests and plant species in indigenous territory and national park Isiboro-Sécure, Bolivia. Economic Botany 63, 229–241 (2009).
Thomas, S. C., Halpern, C. B., Falk, D. A., Liguori, D. A. & Austin, K. A. Plant diversity in managed forests: understory responses to thinning and fertilization. Ecological Applications 9, 864–879 (1999).
Thothathri, K. A Contribution on some Plants from Champaran District, North Bihar. Indian Forester 91, 743–746 (1965).
Tiner, R. W. Field guide to coastal wetland plants of the southeastern United States. (Univ of Massachusetts Press, 1993).
Tinley, K. Dikdik Madoqua kirki in South West Africa: notes on distribution, ecology, and behaviour. Madoqua 1, 7–33 (1969).
Titus, J. H., Titus, P. J., Nowak, R. S. & Smith, S. D. Arbuscular mycorrhizae of Mojave Desert plants. Western North American Naturalist 327–334 (2002).
Tiwari, S. & Maheshwari, J. The Commelinaceae of Madhya Pradesh. Indian Forester 91, 580–590 (1965).
Tiwari, S. & Maheshwari, J. The Orchids of Madhya Pradesh. Indian Forester 89, 426–444 (1963).
Todd, S. & Hoffman, M. A fence-line contrast reveals effects of heavy grazing on plant diversity and community composition in Namaqualand, South Africa. Plant Ecology 142, 169–178 (1999).
Townsend, C. C. Amaranthaceae. in Flowering Plants - Dicotyledons: Magnoliid, Hamamelid and Caryophyllid Families (eds. Kubitzki, K., Rohwer, J. G. & Bittrich, V.) 70–91 (Springer Berlin Heidelberg, 1993). doi:10.1007/978-3-662-02899-5-7.
Tucker, N. I. & Murphy, T. M. The effects of ecological rehabilitation on vegetation recruitment: some observations from the Wet Tropics of North Queensland. Forest ecology and management 99, 133–152 (1997).

Uddin, M. B., Steinbauer, M. J., Jentsch, A., Mukul, S. A. & Beierkuhnlein, C. Do environmental attributes, disturbances and protection regimes determine the distribution of exotic plant species in Bangladesh forest ecosystem? Forest Ecology and Management 303, 72–80 (2013).

Uniyal, M. Medicinal plants of Bhagirathi valley lying in Uttarkashi Forest Division. (1968).

Uprety, Y., Poudel, R. C., Asselin, H. & Boon, E. Plant biodiversity and ethnobotany inside the projected impact area of the Upper Seti Hydropower Project, Western Nepal. Environment, Development and Sustainability 13, 463–492 (2011).

Urgenson, L. S. Ecological and social aspects of riparian restoration and non-native plant invasions: Studies from the Pacific Northwest, US and the Western Cape, South Africa. (University of Washington, 2011).

Valladares, F., Skillman, J. B. & Pearcy, R. W. Convergence in light capture efficiencies among tropical forest understory plants with contrasting crown architectures: a case of morphological compensation. American Journal of Botany 89, 1275–1284 (2002).

van Andel, T. & Havinga, R. Sustainability aspects of commercial medicinal plant harvesting in Suriname. Forest Ecology and Management 256, 1540–1545 (2008).

Van Andel, T. et al. Prioritizing West African medicinal plants for conservation and sustainable extraction studies based on market surveys and species distribution models. Biological Conservation 181, 173–181 (2015).

Van Andel, T., Myren, B. & Van Onselen, S. Ghana's herbal market. Journal of Ethnopharmacology 140, 368–378 (2012).

Van Cleve, K. & Viereck, L. A. Forest succession in relation to nutrient cycling in the boreal forest of Alaska. in Forest succession 185–211 (Springer, 1981).

Van Gemerden, B. S., Shu, G. N. & Olff, H. Recovery of conservation values in Central African rain forest after logging and shifting cultivation. Biodiversity & Conservation 12, 1553–1570 (2003).

van Wilgen, B. W. et al. An assessment of the effectiveness of a large, national-scale invasive alien plant control strategy in South Africa. Biological Conservation 148, 28–38 (2012).

van Wilgen, B. W., Le Maitre, D. & Forsyth, G. The prioritization of species and primary catchments for the purposes of guiding invasive alien plant control operations in the terrestrial biomes of South Africa. Unpublished report. CSIR Natural Resources and the Environment, Stellenbosch (2008).

Van Wyk, B. & Van Wyk, P. Field guide to trees of southern Africa. (Struik, 1997).

Van Wyk, B. A photographic guide to wild flowers of South Africa. (Struik, 2000).

Vanderpoorten, A., Delescaille, L.-M. & Jacquemart, A.-L. The bryophyte layer in a calcareous grassland after a decade of contrasting mowing regimes. Biological Conservation 117, 11–18 (2004).

Verma, B., Singh, B., Saraf, N. & Monappa, K. Suitability and economics of grasses for reclamation and stabilisation of Mahi ravines in Gujarat. Indian Forester 95, 33–44 (1969).

Vesey-Fitzgerald, D. F. The vegetation of central and eastern Arabia. The Journal of Ecology 779–798 (1957).

Vesey-Fitzgerald, D. F. The vegetation of the Red Sea coast north of Jedda, Saudi Arabia. The Journal of Ecology 547–562 (1957).

Vila, M. & Weiner, J. Are invasive plant species better competitors than native plant species?–evidence from pair-wise experiments. Oikos 105, 229–238 (2004).

Villagrán, C., Armesto, J. & Kalin Arroyo, M. Vegetation in a high Andean transect between Turi and Cerro León in northern Chile. Plant Ecology 48, 3–16 (1981).

Vogts, A. Plant wax alkanes and alkan-1-ols in ocean sediments as indicators of continental climate change–validation of a molecular proxy. (Universität Oldenburg, 2011).

Vogts, A., Moossen, H., Rommerskirchen, F. & Rullkötter, J. Distribution patterns and stable carbon isotopic composition of alkanes and alkan-1-ols from plant waxes of African rain forest and savanna C 3 species. Organic Geochemistry 40, 1037–1054 (2009).

von Oheimb, G., Friedel, A., Bertsch, A. & Härdtle, W. The effects of windthrow on plant species richness in a Central European beech forest. Plant Ecology 191, 47–65 (2007).

Vyas, L. & Ramdeo, K. Contribution to the Flora of Rajasthan from Udaipur. Indian Forester 91, 672–675 (1965).

Wace, N. The botany of the southern oceanic islands. Proceedings of the Royal Society of London. Series B, Biological Sciences 152, 475–490 (1960).

Wagner, S. T. et al. Major trends in stem anatomy and growth forms in the perianth-bearing Piperales, with special focus on Aristolochia. Annals of Botany mcu044 (2014).

Walton, B. A. Vegetation patterns and dynamics of renosterveld at Agter-Groeneberg conservancy, Western Cape, South Africa. (Stellenbosch: University of Stellenbosch, 2006).

Wana, D. & Woldu, Z. Vegetation of Chencha highlands in southern Ethiopia. SINET: Ethiopian Journal of Science 28, 109–118 (2005).

Wang, G. The western Ordos plateau as a biodiversity center of relic shrubs in arid areas of China. Biodiversity and Conservation 14, 3187–3200 (2005).

Wang, H. & Chen, H. Plant functional groups based on vegetative and reproductive traits in a subtropical forest community. Journal of forest research 18, 482–490 (2013).

Wang, X.-H., Kent, M. & Fang, X.-F. Evergreen broad-leaved forest in Eastern China: its ecology and conservation and the importance of resprouting in forest restoration. Forest Ecology and Management 245, 76–87 (2007).

Wardle, P. Plant communities of Westland National Park (New Zealand) and neighbouring lowland and coastal areas. New Zealand Journal of Botany 15, 323–398 (1977).

Watkins, G. & others. Trees and shrubs for planting in Tanganyika. Trees and shrubs for planting in Tanganyika. (1960).

Watson, D. M. Determinants of parasitic plant distribution: the role of host quality This article is one of a collection of papers based on a presentation from the Stem and Shoot Fungal Pathogens and Parasitic Plants: the Values of Biological Diversity session of the XXII International Union of Forestry Research Organization World Congress meeting held in Brisbane, Queensland, Australia, in 2005. Botany 87, 16–21 (2008).

Weber, E. Invasive plant species of the world: a reference guide to environmental weeds. (CABI, 2017).

Weber, E., Sun, S.-G. & Li, B. Invasive alien plants in China: diversity and ecological insights. Biological Invasions 10, 1411–1429 (2008).

Welling, P. & Laine, K. Characteristics of the seedling flora in alpine vegetation, subarctic Finland, I. Seedling densities in 15 plant communities. in Annales Botanici Fennici 69–76 (JSTOR, 2000).

Werger, M. & Coetzee, B. A phytosociological and phytogeographical study of Augrabies Falls National Park, Republic of South Africa. Koedoe 20, 11–51 (1977).

White, K. L. Shrub-Carrs of Southeastern Wisconsin. Ecology 46, 286–304 (1965).

Whitehead, F. A study of the relation between growth form and exposure on Monte Maiella, Italy. Journal of Ecology 42, 180–186 (1954).

Whittaker, R. & Niering, W. Vegetation of the Santa Catalina Mountains, Arizona. I. Ecological classification and distribution of species. Journal of the Arizona Academy of Science 3, 9–34 (1964).

Whittaker, R. H. Vegetation of the great smoky mountains. Ecological Monographs 26, 1–80 (1956).

Whittaker, R. H. Vegetation of the Siskiyou mountains, Oregon and California. Ecological monographs 30, 279–338 (1960).
Whittaker, R. J. & Jones, S. H. The role of frugivorous bats and birds in the rebuilding of a tropical forest ecosystem, Krakatau, Indonesia. Journal of Biogeography 245–258 (1994).
Whittaker, R. J., Bush, M., Partomihardjo, T., Asquith, N. & Richards, K. Ecological aspects of plant colonisation of the Krakatau Islands. GeoJournal 28, 201–211 (1992).
Whittaker, R., Niering, W. & Crisp, M. Structure, pattern, and diversity of a mallee community in New South Wales. Plant Ecology 39, 65–76 (1979).
Willems, J. H. Growth form spectra and species diversity in permanent grassland plots with different management. Sukzession auf Grünlandbrachen 35–43 (1985).
Williams, P., Ogle, C., Timmins, S., La Cock, G. & Reid, V. Biology and ecology of Senecio glastifolius and its spread and impacts in New Zealand. Science for Conservation 112, 23 (1999).
Williams, V. L., Witkowski, E. T. & Balkwill, K. Volume and financial value of species traded in the medicinal plant markets of Gauteng, South Africa. International Journal of sustainable development & world ecology 14, 584–603 (2007).
Wilson, B. F. Shrub stems: form and function. Plant stems: physiology and functional morphology 91–102 (1995).
Wilson, G. W., Hartnett, D. C., Smith, M. D. & Kobbeman, K. Effects of mycorrhizae on growth and demography of tallgrass prairie forbs. American Journal of Botany 88, 1452–1457 (2001).
Wilson, J. B. & Agnew, A. D. Positive-feedback switches in plant communities. Advances in ecological research 23, 263–336 (1992).
Wiser, S. K., Bellingham, P. J. & Burrows, L. E. Managing biodiversity information: development of New Zealand's National Vegetation Survey databank. N. Z. J. Ecol. 25, 1–17 (2001).
Woodward, F. & Kelly, C. The influence of CO2 concentration on stomatal density. New Phytologist 131, 311–327 (1995).
Wright, I. J. et al. The worldwide leaf economics spectrum. Nature 428, 821–827 (2004).
Xia, J. & Wan, S. Global response patterns of terrestrial plant species to nitrogen addition. New Phytologist 179, 428–439 (2008).
Xin-Rong, L. Study on shrub community diversity of Ordos Plateau, Inner Mongolia, northern China. Journal of Arid Environments 47, 271–279 (2001).
Yang, X. & Xu, M. Biodiversity conservation in Changbai Mountain Biosphere Reserve, northeastern China: status, problem, and strategy. Biodiversity and Conservation 12, 883–903 (2003).
Yang, Y., Niu, Y., Cavieres, L. A. & Sun, H. Positive associations between the cushion plant Arenaria polytrichoides (Caryophyllaceae) and other alpine plant species increase with altitude in the Sino-Himalayas. Journal of Vegetation Science 21, 1048–1057 (2010).
Yineger, H. A study on the ethnobotany of medicinal plants and floristic composition of the dry Afromontane forest at Bale Mountains National Park, Ethiopia. Addis Ababa University (2005).
Yoccoz, N. et al. DNA from soil mirrors plant taxonomic and growth form diversity. Molecular ecology 21, 3647–3655 (2012).
Yong-Zhong, S., Yu-Lin, L., Jian-Yuan, C. & Wen-Zhi, Z. Influences of continuous grazing and livestock exclusion on soil properties in a degraded sandy grassland, Inner Mongolia, northern China. Catena 59, 267–278 (2005).
Yuan, Z.-Y. et al. Nitrogen resorption from senescing leaves in 28 plant species in a semi-arid region of northern China. Journal of Arid Environments 63, 191–202 (2005).
Zanne, A. et al. Data from: Three keys to the radiation of angiosperms into freezing environments. Nature (2013) doi:10.5061/dryad.63q27.2.

Zeleke, A. A. Bush Encroachme Plant Biodivers. (Addis Ababa University, 2009).
Zhang, J. & Cao, M. Tropical forest vegetation of Xishuangbanna, SW China and its secondary changes, with special reference to some problems in local nature conservation. Biological Conservation 73, 229–238 (1995).
Zhang, J.-T. & Zhang, F. Diversity and composition of plant functional groups in mountain forests of the Lishan Nature Reserve, North China. Botanical Studies 48, 339–348 (2007).
Zhang, Y., Chen, Y. & Pan, B. Distribution and floristics of desert plant communities in the lower reaches of Tarim River, southern Xinjiang, People's Republic of China. Journal of Arid Environments 63, 772–784 (2005).
Zhao, M. et al. Data from: Structure of the epiphyte community in a tropical montane forest in SW China. PLOS ONE (2015) doi:10.5061/dryad.r9j7b.
Zimmerman, J. C., DeWald, L. E. & Rowlands, P. G. Vegetation diversity in an interconnected ephemeral riparian system of north-central Arizona, USA. Biological Conservation 90, 217–228 (1999).
